# Studies on *Xenopus laevis *intestine reveal biological pathways underlying vertebrate gut adaptation from embryo to adult

**DOI:** 10.1186/gb-2010-11-5-r55

**Published:** 2010-05-19

**Authors:** Rachel A Heimeier, Biswajit Das, Daniel R Buchholz, Maria Fiorentino, Yun-Bo Shi

**Affiliations:** 1 Section on Molecular Morphogenesis, Laboratory of Gene Regulation and Development, Program in Cellular Regulation and Metabolism (PCRM), Eunice Kennedy Shriver National Institute of Child Health and Human Development (NICHD), National Institutes of Health (NIH), 18 Library Dr., Bethesda, MD 20892, USA; 2 Institute of Environmental Medicine (IMM), Karolinska Institutet (KI), Nobels väg 13, S-171 77, Stockholm, Sweden; 3 Department of Biological Sciences, University of Cincinnati, 832 Rieveschl Hall, Cincinnati, OH 45221-0006, USA; 4 Current address: Mucosal Biology Research Centre, University of Maryland School of Medicine, 20 Penn Street, HSFII, Rm S303, Baltimore, MD 2120-1192, USA

## Abstract

**Background:**

To adapt to its changing dietary environment, the digestive tract is extensively remodeled from the embryo to the adult during vertebrate development. *Xenopus laevis *metamorphosis is an excellent model system for studying mammalian gastrointestinal development and is used to determine the genes and signaling programs essential for intestinal development and maturation.

**Results:**

The metamorphosing intestine can be divided into four distinct developmental time points and these were analyzed with *X. laevis *microarrays. Due to the high level of conservation in developmental signaling programs and homology to mammalian genes, annotations and bioinformatics analysis were based on human orthologs. Clustering of the expression patterns revealed co-expressed genes involved in essential cell processes such as apoptosis and proliferation. The two largest clusters of genes have expression peaks and troughs at the climax of metamorphosis, respectively. Novel conserved gene ontology categories regulated during this period include transcriptional activity, signal transduction, and metabolic processes. Additionally, we identified larval/embryo- and adult-specific genes. Detailed analysis revealed 17 larval specific genes that may represent molecular markers for human colonic cancers, while many adult specific genes are associated with dietary enzymes.

**Conclusions:**

This global developmental expression study provides the first detailed molecular description of intestinal remodeling and maturation during postembryonic development, which should help improve our understanding of intestinal organogenesis and human diseases. This study significantly contributes towards our understanding of the dynamics of molecular regulation during development and tissue renewal, which is important for future basic and clinical research and for medicinal applications.

## Abstract 2

The developmental transcriptome of the Xenopus laevis intestine, from embryo to adult, reveals insights into the regulation of gut development in all vertebrates.

## Introduction

In mammals, intestinal remodeling is essential for adaptation of infants to their new environment upon birth, and for the development of the complex adult gastrointestinal (GI) tract, which begins as they start to eat solid food. Morphologically, the mammalian embryonic intestine is a simple tubular structure consisting of epithelial cells derived from the endoderm [1,2]. During development, the gut endoderm forms a monolayer of rapidly renewing columnar epithelial cells. The absorptive surface of the GI tract increases dramatically as the epithelium folds into the crypts and finger-shaped villi that characterize the mammalian adult small intestine. The development of the mature, self-renewing GI tract is complete in the first few weeks after birth (around weaning) in mice or up to one year after birth (transition to solid food) in humans [1,3-6]. Throughout postnatal life, the epithelium of the GI tract is in a constant state of self-renewal. This process is a result of intestinal stem cells, which reside in the epithelium of the base of each intestinal crypt, and requires continuous coordination of the proliferation, differentiation, and death programs [1,2]. Thus, the intestine represents a good model to study both tissue development and cell renewal. Despite intensive studies and interest, the factors that mediate maturation of the intestine and cell renewal remain poorly understood, in part due to the difficulty of accessing and manipulating postembryonic development in mammals.

Amphibian metamorphosis shares strong similarities with postembryonic development in mammals, a period spanning several months prior to birth to several months after birth in humans when intestinal maturation takes place [7,8]. It offers a unique opportunity to study the complexities involved during organogenesis and cell regeneration in vertebrate development. Morphologically, tadpole intestine (comparable to the mammalian embryonic intestine) is a simple tubular structure mainly consisting of a single layer of primary/larval epithelium [9]. As the diet of the tadpole (herbivore) changes during metamorphosis to that of a frog (carnivore), the intestine undergoes morphogenetic transformations to form the complex adult intestine. More specifically, the larval epithelial cells undergo degeneration through programmed cell death or apoptosis [9]. Concurrently, stem cells of the adult epithelium develop *de novo *and proliferate. Eventually, they differentiate to form a multi-folded epithelium surrounded by well-developed connective tissue and muscles, producing an organ that resembles and functions like adult mammalian intestine. Even though mammals do not undergo metamorphosis *per se*, the mammalian intestine progresses through homologous fetal and postnatal developmental processes.

A major advantage of metamorphosis in amphibians such as *Xenopus laevis *is that all the changes described above are initiated and controlled by a single hormone, thyroid hormone (T3), through gene regulation via the T3 receptor (TR) [8,10]. Interestingly, endogenous T3 peaks at the climax of metamorphosis when the most metamorphic changes and organ maturation are occurring. Likewise, high levels of T3 are present in human fetal plasma during the several months around birth, the postembryonic period of extensive organ development and maturation [7]. As in amphibians, T3 is an important regulator of intestinal mucosal development and differentiation, including during weaning in mice and rats when adult-type digestive enzymes begin to be produced [11].

Despite numerous studies describing the cellular mechanisms for intestinal remodeling in amphibians and mammals during development, little is known regarding the molecular mechanisms that regulate embryonic-to-adult intestinal transformation. In addition, distinction between embryonic- and adult-specific genes has remained essentially unexplored. This latter point is of critical importance as we are now aware that changes in gene expression early in development can have significant consequences later in life. Toward addressing these issues, we performed genome-wide microarray analyses of *X. laevis *intestinal tissue to systematically determine the changes in signaling pathways during natural metamorphosis. To represent the spectrum of genetic programs associated with the remodeling process, intestines of *X. laevis *tadpoles from pre-metamorphosis (stage 53), pro-metamorphosis (stage 58, when larval cell death begins), metamorphic climax (stage 61/62, when cell death is near completion and cell proliferation as well as adult epithelial cell differentiation take place), and the end of metamorphosis (stage 66, when adult epithelium is formed) were isolated and analyzed. Our bioinformatics analysis on the developmentally regulated functional gene categories provides an understanding of their potential roles during metamorphosis, and thus likely during postembryonic vertebrate GI tract transformation in general. Furthermore, we identified a number of embryonic- and adult-specific genes and pathways in the intestine, which likely have conserved roles in amphibians and mammals in either GI developmental remodeling or the physiological functioning of the embryonic and adult intestine.

## Results and discussion

### Morphological assessment of intestinal remodeling during spontaneous metamorphosis

**Figure 1:**
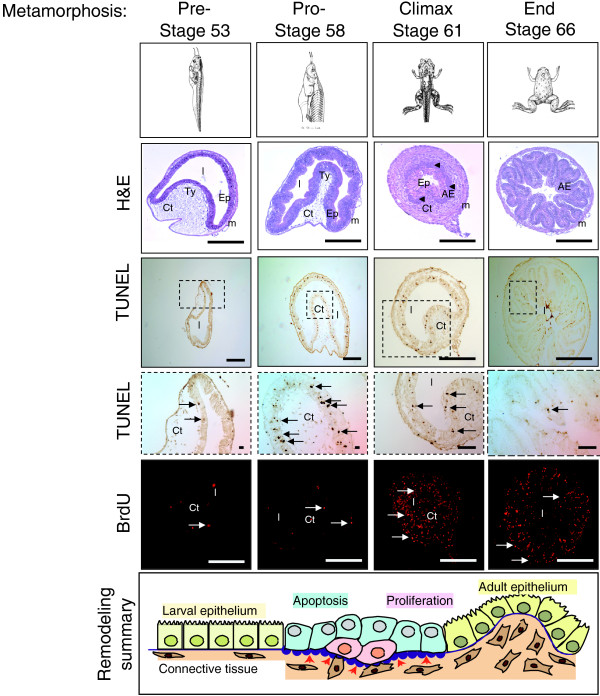
**Morphological, histological and gene expression changes associated with *X. laevis *intestinal remodeling during natural metamorphosis**. Representative metamorphic stages and the corresponding intestine evaluated with H&E (arrowheads indicate the islets of proliferating cells), TUNEL assay (arrows indicate the apoptotic cells), and BrdU immunohistochemistry (arrows indicate proliferating cells). Scale bar = 100 μm. AE:adult epithelial; Ct: connective tissue; Ep: epithelium; m: muscle; Ty: typhlosole. The schematic representation at the bottom summarizes the major changes associated with the stage-dependent transition.

To determine the expression pattern of genes involved in intestinal remodeling, we isolated samples at stages during development that would represent specific time points associated with intestinal development and maturation. Four stages were selected, pre-metamorphosis (stage 53), the end of pro-metamorphosis (stage 58), metamorphic climax (stage 61), and the end of metamorphosis (stage 66) (Figure 1). At the morphological level, the samples selected represented the full spectrum of changes during metamorphosis, including adult cell proliferation and differentiation. The pre-metamorphic intestine, when there is no detectable T3 in the plasma [12], is a simple tube like structure with a single infolding, referred to as the typhlosole, and contains mostly larval epithelial cells. By stage 58, when endogenous T3 is present and metamorphosis has begun, larval epithelial cell death begins and the thin larval muscle and connective tissue layers in the intestine begin to increase in thickness. At stage 61 when plasma T3 is near peak levels, there is an evident increase in both muscle and connective tissue of the intestine and proliferating adult epithelial cells can be identified histologically. At stage 66, the typhlosole is obsolete, and an adult intestinal structure resembles mammalian mature intestines. At the cellular level, a TUNEL assay showed significant larval epithelial cell death at stage 58, while 5-bromo-2-deoxyuridine (BrdU) labeling revealed profound adult cell proliferation at stage 61. Thus, the histological analysis revealed that the stages selected for RNA collection represent the major distinct phases of intestinal remodeling.

### Gene expression profiles of the remodeling intestine during development

**Figure 2:**
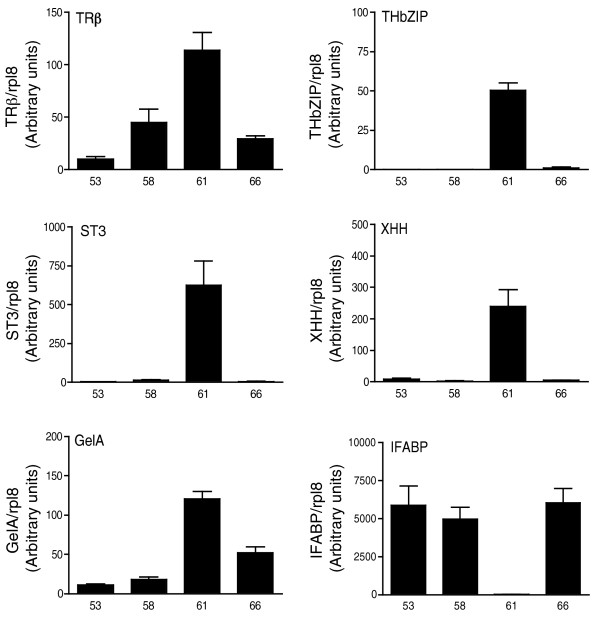
**Expression changes of *TRβ*, *THb*/*ZIP*, *ST3*, *XHH*, *GelA *and *IFABP*, which are established intestinal remodeling markers, during natural development**. The results are expressed relative to the control *rpl8*.

To ensure that the RNA samples do indeed represent signature gene expression patterns of intestinal remodeling during development, we assessed the expression of several genes known to be regulated by T3 during metamorphosis from each RNA sample prior to microarray analysis. These include five up-regulated (*TRβ*, *ST3 *(stromelysin-3), *TH*/*bZIP *(T3-responsive basic leucine zipper transcription factor), *XHH *(sonic hedgehog), *GelA *(gelatinase A)) and one down-regulated (*IFABP *(intestinal fatty acid binding protein)) gene. The expression kinetics of these genes confirmed that the RNA samples collected represented pre-metamorphosis, pro-metamorphosis, metamorphic climax and the end of metamorphosis. Their expression patterns at the isolated stages all agreed with their known profiles (Figure 2).

**Figure 3:**
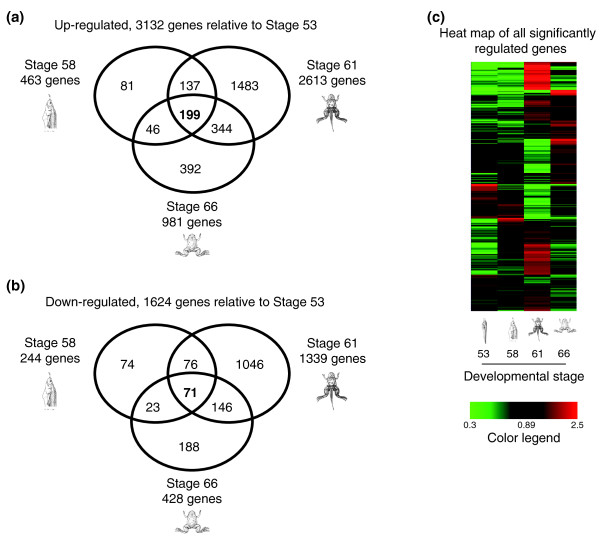
**Genes significantly up- and down-regulated in the intestine during natural metamorphosis at specific stages when compared to stage 53**. Venn diagrams showing the number of genes significantly **(a) **up-regulated and **(b) **down-regulated in the intestine during natural metamorphosis when the indicated stages were compared to stage 53 by microarray. **(c) **Temporal changes in gene expression during natural development visualized by heatmap. Normalized mean-centered expression levels for each gene are shown with black representing mean expression levels of four stages for a given gene, and green and red indicating lower or higher than the average as shown in the color legend.

**Figure 4:**
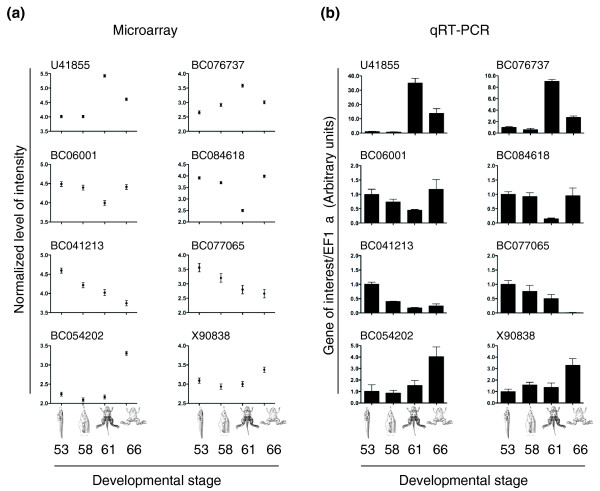
**Confirmation of gene regulation patterns identified by microarray with RT-qPCR**. **(a) **Microarray. **(b) **RT-qPCR. GenBank accession numbers are shown above the graphs. The vertical axis in (a) shows the normalized log intensity of the expression and in (b) shows the expression of the genes with stage 53 arbitrarily set to 1.

To obtain a perspective on global gene expression changes during intestinal development, we performed a pair-wise comparison of gene expression microarray data for each stage and observed that 3,132 and 1,624 genes were significantly up- and down- regulated, respectively, with a fold change ≥1.5 between at least two of the stages (Table S3A, B in Additional file 1). When the expression levels at stages 58, 61, and/or 66 were compared to those in the larval intestine at stage 53, stage 61 had the most number of genes up- and down-regulated (Figure 3a, b), which agrees with the fact that this is the climax stage, when most drastic changes are taking place. This is more clearly demonstrated by a heat map of the relative gene expression levels of each of the regulated genes during stages 53 to 66 (Figure 3c), which shows a lot more highly expressed (in red) and lowly expressed genes (in green) at stage 61 compared to the other stages. Among the regulated genes (relative to stage 53), 199 were commonly up-regulated and 71 were commonly down-regulated for all three developmental stages (58, 61 and 66). The highest number of shared regulated genes was between stages 61 and 66, suggesting that many genes up-regulated by stage 61 continue to function by the end of metamorphosis. In contrast, stages 58 and 66 shared the least number of regulated genes, indicating distinct gene expression programs at these two developmental stages, consistent with the fact that one is preparing the animal for climatic changes while the other is finishing these changes. While validation of all the genes identified was not practical, we chose a representative sample that was subsequently analyzed by quantitative reverse-transcription PCR (RT-qPCR) to verify the microarray trends (Figure 4a, b; Table S4 in Additional file 1) of genes that were significantly regulated by ≥1.5 based on the microarray analysis. We used independently isolated intestinal RNA and found that 81 of the 84 genes analyzed by RT-qPCR agreed with the microarray data (Figure 4a, b; Table S4 in Additional file 1). In addition, we also performed *in situ *hybridization on intestinal sections for representative genes and the results for all genes with detectable *in situ *signals were consistent with the microarray expression profiles (Figure S1 in Additional file 2; also see below).

### Global outlook on the temporal pattern of expression and functional classification of these genes during intestinal remodeling

**Figure 5:**
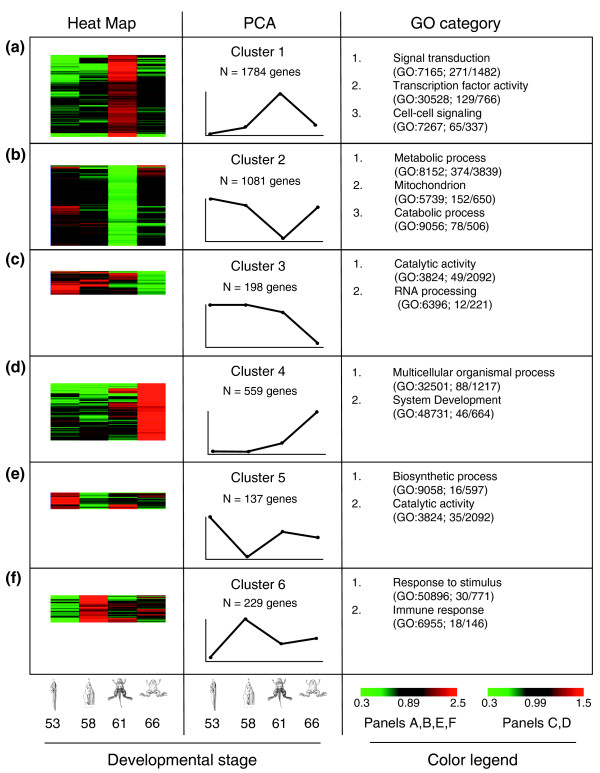
**Regulated genes can be grouped into six clusters based on developmental regulation patterns**. The number of genes in each cluster is indicated in the schematic diagram. **(a, b) **Clusters 1 and 2 represent genes that are predominantly regulated at metamorphic climax, with the former following the endogenous T3 concentration. **(c, d) **Clusters 3 and 4 include genes with higher levels of expression in tadpoles and frogs (larval- and adult-enriched genes), respectively. **(e, f) **Clusters 5 and 6 are genes up- or down-regulated mainly at stage 58. All clusters were evaluated by GO analysis and two or more examples of the significantly regulated GO categories that had >60 genes (clusters 1 and 2) and >5 genes (clusters 3 to 6) regulated during metamorphosis are listed. A complete list of GO categories associated with each cluster is listed in Table S6 in Additional file 1. PCA: principal component analysis.

To identify molecular pathways involved in GI tract development and maturation, we used principal component analysis, which quantitatively grouped the developmental changes in gene expression into six major clusters [13] (Figure 5; and Table S5 in Additional file 1), providing an overview of global expression trends during development. The six clusters were defined according to the pattern of expression they exhibited: cluster 1, up-regulated (1,784 genes); cluster 2, down-regulated (1,081 genes); cluster 3, larval enriched (198 genes); cluster 4, adult enriched (559 genes); cluster 5, early down-regulated (137 genes) and early up-regulated (229 genes).

To better understand the biological and molecular functions of the genes within the six identified expression clusters, we performed Gene Ontology (GO) classification to identify biological functional categories statistically enriched in each gene cluster based on the human RefSeq homologs [14]. The analysis revealed little or no overlap in the GO categories, suggesting that genes in different clusters have distinct biological functions during development (Figure 5; Tables S6 in Additional file 1). Cluster 1 was the largest and contained many biological pathway categories associated with cell proliferation (GO:0006950), signal transduction (GO:0007165), transcription factor activity (GO:0030528, GO:0006357, GO:0006366, GO:0003700) and cell-cell signaling (GO:0007267), suggesting that the genes in these categories are involved in the climatic remodeling processes (Figure 5a). Of particular interest was the high number of genes associated with transcription from RNA polymerase II promoter (GO:0006357) and its regulation (GO:0006366), and the transcription factor category (GO:0003700). Thus, transcriptional regulation and signaling pathways are important events needed at the climax of metamorphosis when tissue remodeling and cell proliferation takes place. The genes within the cluster 1 GO categories appear to be T3-dependent as their expression levels follow the endogenous levels of T3. Cluster 2 is the second largest cluster and contains down-regulated genes that are associated with metabolic (GO:0008152) and catabolic processes (GO:0009056) (Figure 5b). Metabolic pathways such as glycolysis, digestion and the complexes that transfer electrons and synthesize ATP in the mitochondrial inner membrane all appear to shut down at metamorphic climax and start again at the end of metamorphosis. These changes are likely important for the larval cells to undergo apoptosis and may be associated with a shunt in dietary needs, as the animal does not feed during metamorphosis [15].

The genes that belong to cluster 3, larval enriched genes, included GO categories associated with catalytic activity (GO:0003824) and RNA processing (GO:0006396), while cluster 4, adult-enriched genes, included GO categories that are involved in multicellular organismal processes (GO:0032501) and system development (GO:0048731) (Figure 5c, d). Genes belonging to catalytic activity and RNA processing GO categories were highly enriched in the larval stage of development but not at the end of metamorphosis, suggesting that they are required prior to the initiation of DNA replication during transcription to drive cell cycle progression and the other downstream processes described for cluster 1. Conversely, the enrichment of GO categories related to multicellular organismal processes and system development at the end of metamorphosis suggests that the up-regulation of these processes is required for the maturation of the adult organ and/or the physiological function of the adult organ. The remaining clusters are small but do include some interesting GO categories. For example, the transient down-regulation of the GO categories involved in either biosynthetic processes or biosynthetic catalytic activity (cluster 5; Figure 5e) is consistent with apoptosis as an early event during metamorphosis, while the increase in the expression of genes associated with immune response (cluster 6; Figure 5f) may likely be associated with apoptotic removal of larval cells.

### Using established biological processes to identify pathways that are regulated during development

**Figure 6:**
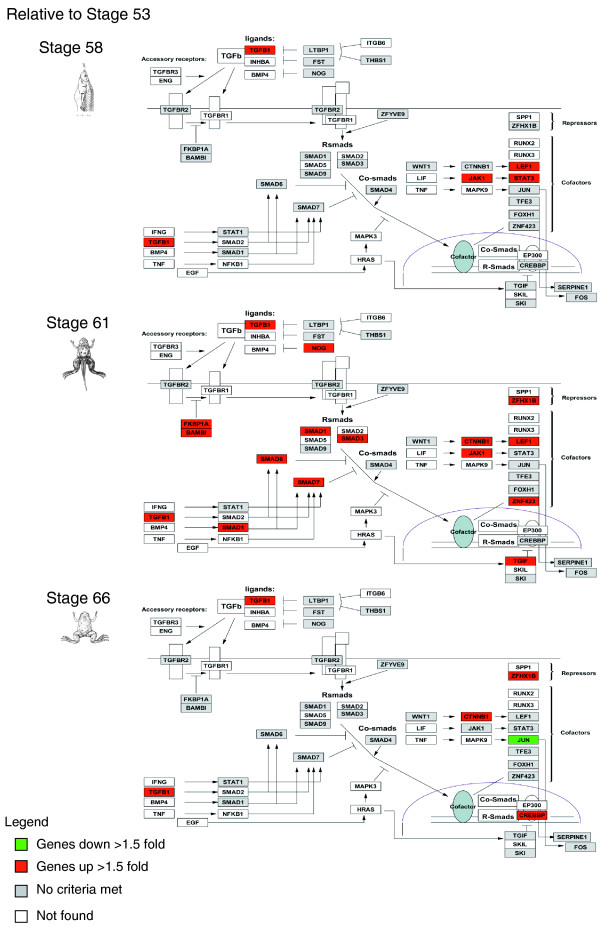
**Temporal regulation of a significantly regulated biological pathway, the TGF-β pathway, during intestinal remodeling**. Genes that are up- or down-regulated at stages 58, 61 and 66 relative to stage 53 are shown in red and green, respectively.

GenMAPP software, which categorizes genes into established pathways associated with biological processes and diseases, was used to analyze our expression data in the context of established pathway collections of biological processes and diseases to identify significantly regulated pathways. Of particular interest were the genes that were significantly up- or down- regulated at metamorphic climax (stage 61) and thus more likely to contribute to the putative developmental programs dependent on T3 regulation. Among the significantly up-regulated pathways during intestinal remodeling is the transforming growth factor-beta (TGF-β) signaling pathway (Figure 6). As the tadpole progressed from stage 53 to stage 58, four genes of the pathway were up-regulated. By stage 61, 15 genes were up-regulated, and by stage 66, the number of genes up-regulated compared to stage 53 were only 5, and one gene was now down-regulated. These results suggest that up-regulation of the TGF-β pathway is important for the remodeling taking place at the climax (stage 61) of metamorphosis. Interestingly, disruptions to TGF-β signaling have been associated with cancer [16]. This pathological effect is likely related to the mis-regulation of apoptosis and/or cell proliferation as implied from the correlation observed during intestinal remodeling.

**Figure 7:**
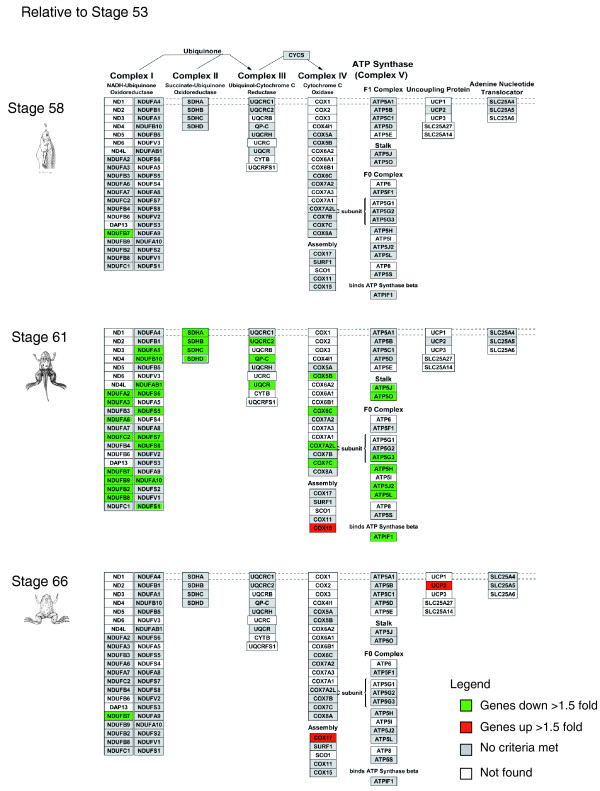
**Temporal regulation of the electron transport pathway during intestinal remodeling**. Genes that are up- or down-regulated at stages 58, 61 and 66 relative to stage 53 are shown in red and green, respectively.

Conversely, among the biological pathways significantly down-regulated during development, the electron transport chain is of particular interest (Figure 7). There was only one gene in the pathway that was down-regulated at stage 58. On the other hand, at climax (stage 61), about 30 genes were down-regulated. By the end of metamorphosis, the expression of these genes returned to pre-metamorphic levels. Thus, at climax, down-regulation of the electron transport chain is correlated with the massive apoptosis in the larval epithelium and indicates that energy synthesis via ATP rapidly halts or is inhibited. As ATP production closely matches the metabolic state of the cell, the down-regulation of this pathway may reflect the fact that most cells are apoptotic at the climax and thus relatively metabolically inactive [15].

### Meta-analysis: comparison with expression profiles during T3-induced intestinal remodeling

A unique advantage of frog metamorphosis is that it can be induced precociously by adding physiological levels of T3 in the rearing water of pre-metamorphic tadpoles. We have previously carried out a microarray analysis to search for genes that were regulated after T3 treatment of pre-metamorphic tadpoles [17]. Genes that are commonly regulated during both natural and T3-induced intestinal metamorphosis are more likely to play important roles for the transformation process and are thus of high value for future studies. Accordingly, we compared the genes that were regulated at stages 58, 61, and 66 (compared to stage 53) from the present study to those regulated after 1, 3, and 6 days of T3 treatment of stage 52/54 tadpoles (compared to untreated animals) [17]. The intestine of tadpoles treated with T3 for 3 to 6 days had extensive cell death and adult cell proliferation but no adult epithelial differentiation, and thus resembled that at stages 58 to 61 during natural metamorphosis [17]. Molecularly, 5.7, 20.9 and 33% of the genes up-regulated at stage 58 overlapped with genes up-regulated after 1, 3, and 6 days of T3 treatment, respectively (Table 1). A similar percentage overlap was observed for genes down-regulated at stage 58 with those down-regulated after 1, 3, and 6 days of T3 treatment (Table 1). Of the genes up-regulated at stage 66, 4.6, 16.9 and 17.6% overlapped with the up-regulated genes after 1, 3, and 6 days of T3 treatment, respectively, and again similar results were found for the down-regulated genes. Of particular interest is the finding that at the molecular level, stage 58 and 61 tadpole intestines resemble those from pre-metamorphic tadpoles treated with T3 for 3 and 6 days, respectively, in agreement with the histological and histochemical findings [17]. However, even though similar histological changes occur during induced and spontaneous metamorphosis, the gene expression changes associated with intestine remodeling after T3 induction do not strictly correlate with those of specific natural developmental stages, likely due to different levels of T3.

**Table 1:** Overlap of significantly regulated genes between the T3-induced and spontaneous metamorphosis microarrays

	Overlap for up-regulated genes			Overlap for down-regulated genes		
	Day 1 T3	Day 3 T3	Day 6 T3	Day 1 T3	Day 3 T3	Day 6 T3
Stage 58	*36 (*5.7%/8.3%)	*133 *(20.9%/10%)	*210 *(33%/8.8%)	*32 *(9.3%/6.3%)	*95 *(27.7%/6.2%)	*102 *(29.8%/5.9%)
Stage 61	*110 *(3.6%/25.5%)	*489 *(16.2%/36.7%)	*898 *(29.7%/37.8%)	*93 *(5.1%/18.2%)	*398 *(21.6%/25.9%)	*532 *(28.9%/30.5%)
Stage 66	*63 *(4.6%/14.6%)	*231 *(16.9%/17.3%)	*417 *(17.6%/17.6%)	*46 *(7.8%/9%)	*14 *(24.6%/9.5%)	*13 *(23.3%/7.9%)

The number of genes common between those regulated at a developmental stage and those after a particular T3 treatment is shown in italics. The percentages of those commonly regulated genes are given in parentheses after correcting for genes present in both microarray platforms used in the two studies, with the first percentage representing the commonly regulated genes as a percentage of the genes regulated at a developmental stage and the second representing the commonly regulated genes as a percentage of the genes regulated after a T3 treatment.

### Identification of larval- and adult-specific intestinal genes

Tadpole and adult frogs are both free-living animals and require a fully functional intestine. To date, little is known about any potential differences in the gene expression profiles between the two forms. A similar lack of knowledge also exists for the prenatal/neonatal intestine versus the adult intestine in mammals. Our microarray data offer an opportunity to discover larval- versus adult-specific genes. For simplicity, we arbitrarily defined larval- or adult-specific genes as those with ten-fold or more differences in the expression levels between stages 53 (larval) and 66 (adult) on the microarray. This more stringent analysis led to the discovery of 17 larval- and 52 adult-specific genes (Tables 2 and 3). Of these, we were able to find the human homologs for 11 and 30 genes, respectively. The small numbers made it impossible to analyze their functional GO categories. Thus, we evaluated genes individually. Interestingly, a number of larval-specific genes (for example, BC086270 (mucin) and BC060496 (cytochrome P450)), have been described as molecular biomarkers for colon and other human cancers [18-20], suggesting that inappropriate continued expression of larval/embryonic genes in the adult intestine leads to, or is indicative of, cancer development. Among the adult-specific genes, several genes associated with digestion were significantly up-regulated. The expression of these genes is likely important to accommodate the dietary changes.

**Table 2:** Larval specific genes with ≤0.1-fold expression at stage 66 versus stage 53

	GenBank	Gene	Gene name	Fold change
1	AY762616	*UGDH*	UDP-glucose dehydrogenase (mucin)	0.07
2	BC042305	*SLC22A6*	Solute carrier family 22 (organic anion transporter)	0.07
3	BC044073	*ISYNA1*	Inositol-3-phosphate synthase 1	0.03
4	BC044116	*KRT8*	Keratin 8	0.00
5	BC056840	*LTF*	Lactotransferrin	0.07
6	BC060496	*CYP3A4*	Cytochrome P450, family 3, subfamily A, polypeptide 4	0.07
7	BC072842	*TRIM2*	Tripartite motif-containing 2	0.07
8	BC074222	*SLC16A1*	Solute carrier family 16	0.04
9	BC081224	*TXNRD1*	Thioredoxin reductase 1	0.08
10	BC082530	*SLC34A3*	Solute carrier family 34 (sodium phosphate)	0.03
11	BC085055	*ANPEP*	Alanyl (membrane) aminopeptidase	0.02
12	BC086270	*GCNT3*	Glucosaminyl (N-acetyl) transferase 3	0.10
13	BJ057663	*AIFM2*	Apoptosis-inducing factor, mitochondrion-associated, 2	0.05
14	BX844453	*DIO1*	Deiodinase, iodothyronine, type I	0.09
15	CK799950		*Transcribed locus*	0.05
16	DQ096886	*CA14*	Carbonic anhydrase 14	0.08
17	L20816	*PLCB3*	Phospholipase C, beta 3	0.09

**Table 3:** Adult specific genes with ≥10-fold expression at stage 66 versus stage 53

	GenBank	Gene	Gene name	Fold change
1	AF170337		*Transcribed locus*	24.15
2	AY260728	*SLC5A8*	Solute carrier family 5 (iodide transporter)	58.97
3	BC043635	*ARG1*	Arginase	20.07
4	BC045220	*MATN2*	Matrilin 2	12.53
5	BC053814	*INMT*	Indolethylamine N-methyltransferase	24.27
6	BC054155		*Transcribed locus*	53.57
7	BC054202		*Transcribed locus*	18.55
8	BC054284	*HSD11B1*	Hydroxysteroid (11-beta) dehydrogenase 1	15.61
9	BC054987	*NAALADL1*	N-acetylated alpha-linked acidic dipeptidase-like 1	15.98
10	BC056841	*AMY2A*	Amylase	12.21
11	BC056856	*CPA1*	Carboxypeptidase A1	38.45
12	BC059786		*Transcribed locus*	69.65
13	BC059976		*Transcribed locus*	22.40
14	BC061680	*RDH16*	Retinol dehydrogenase 16	24.15
15	BC070669	*ADH1B*	Alcohol dehydrogenase 1B	15.66
16	BC070682	*KRT19*	Keratin 19	16.04
17	BC071004	*SULT2A1*	Sulfotransferase family 3A	11.68
18	BC072097	*ACTA1*	Actin, alpha 1	20.68
19	BC072970	*CELA1*	Chymotrypsin-like elastase family,	176.44
20	BC073555	*CTRB1*	Chymotrypsinogen B1	14.60
21	BC074179	*ABAT*	4-aminobutyrate aminotransferase	14.42
21	BC074200	*HAO2*	Hydroxyacid oxidase 2	11.21
23	BC077848	*HPGD*	Hydroxyprostaglandin dehydrogenase 15-(NAD)	17.23
24	BC078061	*ELA3*	Elastase 3	136.11
25	BC078585	*CELA1*	Chymotrypsin-like elastase family	10.86
26	BC080035	*HLA-DRA*	Major histocompatibility complex, class II, DR alpha	10.65
27	BC080096	*SULT1A1*	Sulfotransferase family, cytosolic 1A	72.69
28	BC081152	*NFIX2*	Nuclear factor I/X2	121.87
29	BC081272	*CALB1*	Calbindin 1, 28kDa	444.83
30	BC082401	*DPP4*	Dipeptidyl-peptidase 4	74.40
31	BC082713		*Transcribed locus*	11.28
32	BC082923	*TNNI3*	Troponin I type 3	22.26
33	BC084270		*Transcribed locus*	24.97
34	BC084428	*MAOB*	Monoamine oxidase B	83.83
35	BC084607		*Transcribed locus*	16.91
36	BC084832	*CPB1*	Carboxypeptidase B1	60.91
37	BC085060		*Transcribed locus*	20.23
38	BC085209	*FAM55D*	Family with sequence similarity 55	11.96
39	BE509325	*ALPI*	Alkaline phosphatase, intestinal	10.59
40	BG160459	*HBG1*	Hemoglobin, gamma A	31.96
41	BG346716		*Transcribed locus*	31.94
42	BG407150		*Transcribed locus*	16.35
43	BX843298		*Transcribed locus*	11.44
44	BX845609	*PRSS2*	Protease, serine, 2 (trypsin 1)	11.63
45	BX846064		*Transcribed locus*	32.00
46	BX846116		*Transcribed locus*	10.45
47	BX849304	*PRSS3*	Chymotrypsin-like elastase family	86.57
48	CB943692		*Transcribed locus*	31.69
49	CD300904		*Transcribed locus*	21.17
50	CF271248	*LGALS1*	Lectin, galactoside-binding, soluble1	21.19
51	CF271543		*Transcribed locus*	34.55
52	L28111	*DIO3*	Deiodinase, iodothyronine, type III	34.53

**Figure 8:**
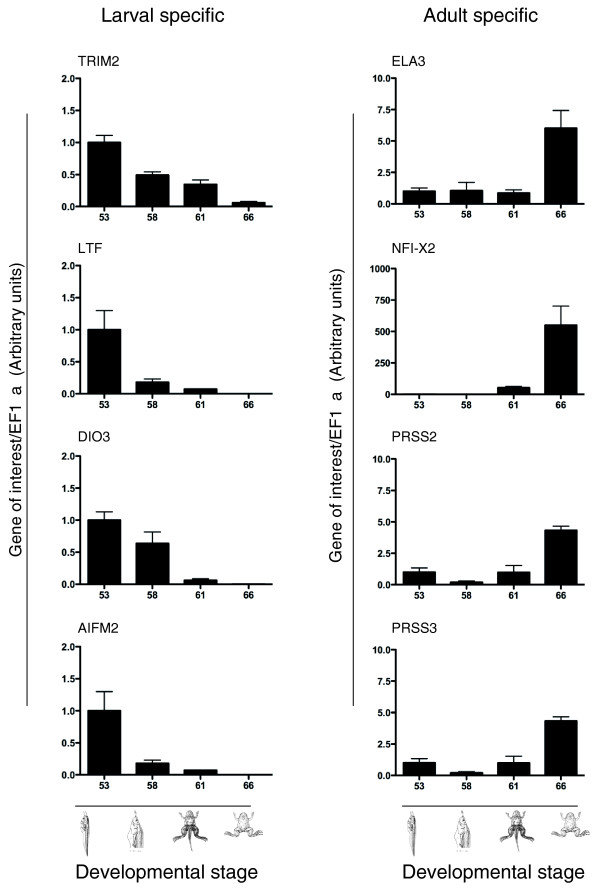
**The expression of larval- and adult-specific genes during natural metamorphosis**. Larval- and adult-specific genes analyzed by RT-qPCR, confirming the larval- and adult-specific designation based on microarray analysis.

**Figure 9:**
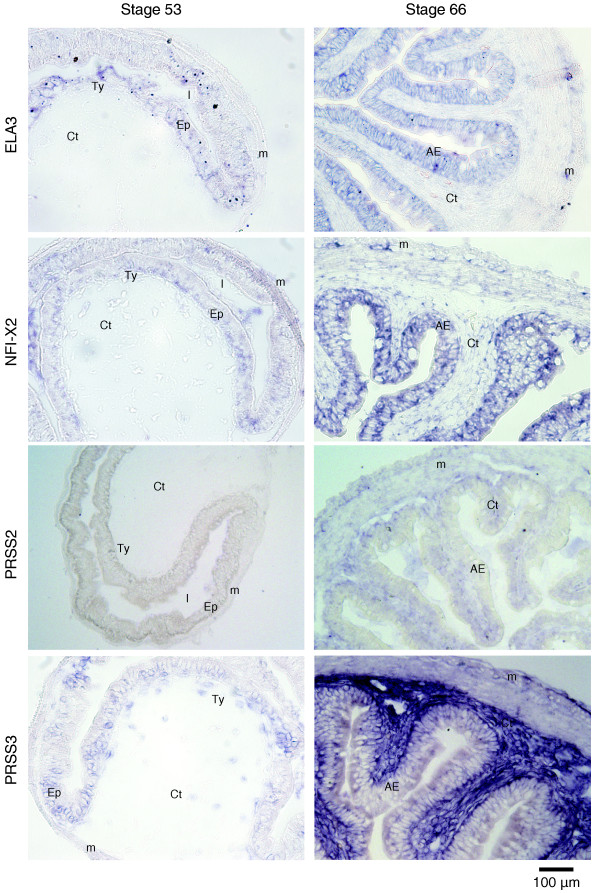
***In situ *hybridization of the four adult-specific genes with intestinal cross-sections at stages 53 and 66**. Scale bar = 100 μm. AE: adult epithelium; Ct: connective tissue; Ep: epithelium; L: lumen; M: muscle; Ty: typhlosole.

To confirm the bioinformatics and determine the cell type specificity of these larval- and adult-specific genes, four genes were randomly selected from each category and evaluated by RT-qPCR and *in situ *hybridization. The RT-qPCR results confirmed the larval and adult gene specificity within the intestine (Figure 8). *In situ *hybridization failed to detect convincing signals for the selected larval-specific genes despite trying multiple different probes, presumably due to low expression levels. On the other hand, of the four adult genes, two were expressed in the epithelium while the other two were in the connective tissue (Figure 9), indicating that the adult-specific genes are not restricted to the epithelium as one might have expected given the dominant role of the epithelium in the physiological function of the adult intestine.

### Parallels between amphibian and mouse gene expression during postembryonic intestinal development

**Figure 10:**
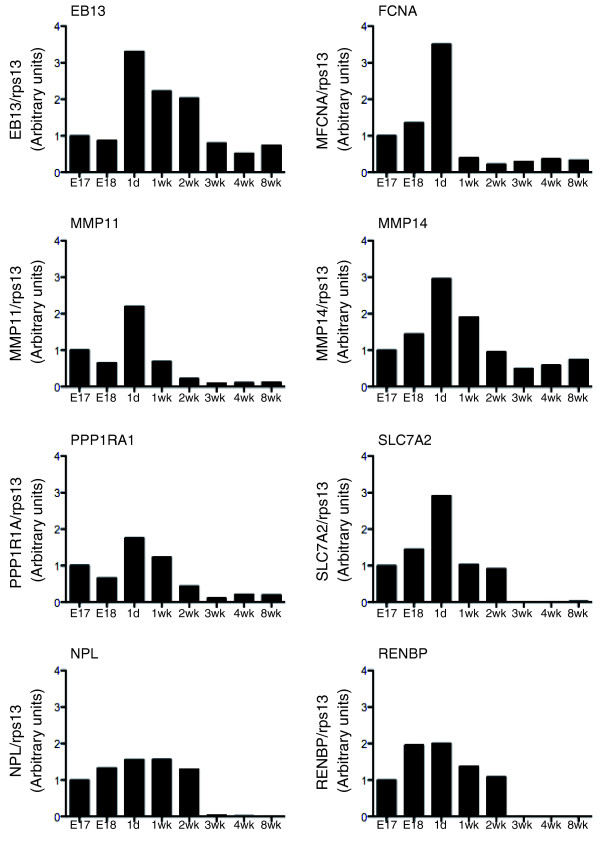
**Gene expression during mouse development**. RT-qPCR analysis was carried out for the indicated genes on mouse intestinal RNA at indicated stages. Note that the expression patterns of mouse *EB13*, *FCNA*, *MMP11*, *MMP14*, *PPP1RA1 *and *SLC7A2 *were similar to those of their homologs in *Xenopus *during metamorphosis as observed by microarray, that is, there were higher levels of expression shortly after birth in the mouse when T3 levels are high, just like at metamorphic climax. The expression of *NPL *and *RENBP *represent profiles of genes that differ from those in *Xenopus*. All RT-qPCR results are expressed relative to the control *RPS13*, with the expression of the genes at E17 arbitrarily set to 1.

To obtain a perspective on gene expression changes during intestinal metamorphosis and mammalian intestinal maturation during postembryonic development, we investigated the possible similarities in gene regulation between *X. laevis *and mouse. As both species are under the influence of increasing levels of circulating T3 during postembryonic development, we focused on genes whose expression is likely upregulated when T3 levels are high. We thus ranked the genes based on relative expression at stage 61 (climax of metamorphosis when T3 level peaks) in comparison to stages 53 (when there is little T3) and 66 (the end of metamorphosis) and identified 68 genes with ≥7-fold higher expression at stage 61 compared to that at stage 53 or 66. Among these 68 genes, we were able to find the corresponding homologs and design primers for 27 genes (the mammalian homologs for many *Xenopus *sequences could not be identified as the cDNA sequences are incomplete). To evaluate the conservation in the gene expression profiles, we obtained commercial mouse intestinal RNA samples from embryonic stages E17 and E18 (comparable to *Xenopus *stages 53 to 58 with regard to T3 levels), around birth (1 day to 2 weeks of age; comparable to amphibian stages 59 to 62), at weaning and adult (3 to 4 and 8 weeks of age, respectively; comparable to amphibian stages 64 to 66) and performed RT-qPCR for all 27 genes (Table S2B in Additional file 1). The vast majority of the genes (19 out of 27 genes) showed a similar pattern of regulation as seen in *Xenopus*, that is, with a peak in gene expression observed in the first 2 weeks after birth when T3 levels are high (Figure 10; Table S2B in Additional file 1). The remaining eight genes had similar levels of expression from E17 to 2 weeks of age (weaning), after which their expression was significantly decreased (not shown). All together, these data suggest that the molecular regulation of intestinal development in different vertebrate species is highly conserved.

## Conclusions

Most vertebrate diets change during development. To date, little is known about the underlying molecular basis responsible for the tissue remodeling needed for this diet change. Here, using *X. laevis *metamorphosis as a model, we investigated gene expression profiles of the intestine during the transition from the larval to adult form. Interestingly, despite the dependency on T3 for intestinal remodeling, meta-analysis revealed that only half of the genes regulated in the intestine during natural development were common with those regulated upon treatment of pre-metamorphic tadpoles with T3, suggesting that significant differences exist between T3-induced and natural metamorphosis. We identified six major gene clusters involved in the intestinal transformation from the embryo to adult form. The two largest clusters have peak and trough expression levels, respectively, at the metamorphic climax, equivalent to around birth in mammals. Moreover, genes induced at metamorphic climax (stage 61) parallel those with expression peaks in mouse intestinal maturation around birth. These and other known conserved genes in vertebrate postembryonic development, especially with regard to the intestine, argue that many of our findings here are likely applicable to mammalian development.

Although the larval to adult remodeling of the epithelium of the *X. laevis *intestine has been well studied at the cellular level, to date, few genes specifically expressed in the larval or adult intestine had been identified. An important contribution of this study is the identification of novel larval/embryonic- and adult-specific (enriched) genes, which provide new insights into the molecular regulation of GI development/function and are likely worthy of further investigation in GI developmental and disease models. We identified 17 larval-specific and 52 adult-specific genes. These genes are likely related to the specific physiological functions of the larval and adult organ. Many adult-specific genes are associated with digestion. For example, dietary enzymes, including the serine proteinases PRSS2 and PRSS3, are significantly increased in the adult intestine. Serine proteinases (trypsins) were only considered to be synthesized in the pancreas, although there was one study showing that they are expressed at high levels in the adult pancreas and small intestine in both humans and mice [21]. Trypsin is essential for food digestion, but is also involved in other physiological and pathological processes, such as inflammation and tumor invasion [22,23]. Aberrant expression of intestinal trypsin may be indicative of pathological processes. Both PRSS2 and PRSS3 are expressed in the connective tissue of the frog but not tadpole intestine. Two other adult-specific genes evaluated, *ELA3 *and *NFI-X2*, were predominantly expressed in the epithelium. These genes may provide good adult-specific markers to evaluate adult epithelial regeneration.

The larval-specific genes vary in their potential functions. We found that the larval-specific genes such as AY762616 (UDP-glucose dehydrogenase) and BC042305 (solute carrier 22A6) may play a role in signal transduction and cell migration, while mucin, cytochrome P450, BC081224 (thioredoxin reductase), BC044116 (keratin 8) and BC056840 (lactotransferrin) are described as potential molecular markers for colon, breast and other cancers [24,25], suggesting that inappropriate expression of larval-specific genes in adult intestine may cause or be indicative of cancer formation. As there has been a surge in human colon cancers since the mid-1980s, the identification of larval- and adult-specific genes as reported here should help to increase our understanding of their role at specific time points during development, and their expression earlier or later during development may represent specific candidate genes as colorectal cancer markers. Furthermore, as the GI is constantly self-renewed, it is important to evaluate potential genes that may act as, or be indicators for, tumor cells with stem-cell-like properties; larval- and adult-enriched categories may prove to be an important starting point in this evaluation.

Although gene expression studies have been recently reported for the digestive tract in zebrafish and mouse [26,27], these species lack the distinct, stage-dependent tissue transformation events that occur during metamorphosis, thus making it difficult to correlate gene expression profile changes with cellular transformations. Secondly, the influence of T3 in zebrafish and mouse GI development and maturation at the molecular level has not been evaluated to the same extent as in amphibians. Amphibian metamorphosis has three distinct developmental stages: pre-metamorphosis, pro-metamorphosis and metamorphic climax. During pre-metamorphosis, the tadpole lacks endogenous T3 but is competent to respond to exogenous T3, which can induce a precocious metamorphosis. Pro-metamorphosis begins (around stage 55) with the maturation of the thyroid gland and low-level secretion of T3, which initiates the first metamorphic changes, such as limb development. Endogenous T3 levels continue to rise and peak dramatically at metamorphic climax (stages 58 to 65), which is characterized by the rapid, overt transformation of the tadpole [8]. Our study reveals that for each developmental stage, many genes were regulated in comparison to the pre-metamorphic (larval) controls (stage 53). Interestingly, the number of genes coordinately/commonly regulated in the three metamorphosing stages (stages 58, 61, 66, when compared to stage 53) was strikingly low, indicative of distinct molecular processes needed for different stages of metamorphosis.

It should be reiterated that the largest cluster of genes involved in amphibian GI development includes genes that were significantly up-regulated at stage 61 (cluster 1), coinciding with the high concentration of endogenous T3. These data suggest that during natural development, most significant changes occur at metamorphic climax and that the genes in these categories are most likely T3-dependent genes that follow the gene expression profile of the established T3 response genes *ST3 *(involved in tissue breakdown) and *XHH *(involved in cell differentiation). The second largest cluster of genes is the inverse of cluster 1, where gene expression is high before climax, then drops dramatically at climax, and then rises again at the end of metamorphosis. The genes in this cluster likely require fully differentiated cells, as indicated by the expression of *IFABP*, a marker for fully differentiated intestinal epithelial cells. Moreover, our comparative analysis revealed that during mammalian intestinal development, the genes that peaked in expression at stages 61, such as *MMP11 *(mouse homolog for amphibian ST3) were also significantly up-regulated at birth in mouse. This suggests that molecular signatures during intestinal development and maturation are highly conserved in different species and that the role of T3 in regulating target genes for intestinal development is of potential importance. Future comparative studies are needed to evaluate the significance of T3 regulation in early gut development in the embryo or fetus.

Using GenMAPP databases to analyze biological pathways that play an important role in GI development, we found that the TGF-β signaling pathway and the electron transport chain are significantly up- and down-regulated at the climax of metamorphosis, respectively (clusters 1 and 2, respectively). The TGF-β signaling pathway has been implicated in both apoptosis and cell proliferation; thus, its up-regulation at metamorphic climax may allow it to participate in both larval epithelial cell death and adult cell proliferation. Clearly, future functional studies are needed to test this. Interestingly, TGF-β has been implicated as a tumor promoter in intestinal epithelial cells that have become resistant to its tumor suppressor activity [28-30]. Our finding that this pathway is involved in the transition from larval to adult intestine suggests that alteration of its normal developmental function may be important for tumor development and metastasis. Therefore, the identification and understanding of the TGF-β pathway and how it is controlled or turned off in natural development in frogs may reveal targets of intervention for treatment of human intestinal cancers. The GO categories in cluster 2 include genes for glucose metabolism and mitochondrial ATP synthesis, such as in the electron transport chain. The latter were also previously identified to be down-regulated in the intestine after T3 treatment of pre-metamorphic tadpoles. Their down-regulation is likely important for the apoptotic removal of larval epithelial cells. Interestingly, insertional mutagenesis screens with zebrafish found that mutations in mitochondrial ATP synthase genes caused both liver and intestinal defects during development. One of the genes significantly regulated in the electron transport chain, the ATP-binding cassette transporter, utilizes energy from ATP hydrolysis to carry out biological processes, including translocation, translation of RNA and DNA repair. Mutation of this gene or disruptions in its expression may lead to a number of inheritable human diseases, such as cystic fibrosis [31]. Therefore, defects in the function of genes in clusters 1 and 2 may significantly contribute to the development of specific GI and other diseases. Clearly, future analyses are required to determine the functional roles of these genes in the development of the vertebrate intestine.

## Materials and methods

### Animals

Tadpoles were purchased from NASCO (Fort Atkinson, WI, USA). All animals were maintained and used in accordance with the guidelines established by NICHD Animal Use and Care Committee.

### RNA extraction and microarray analyses

RNA was isolated and subjected to microarray (Agilent slides AMADID 013665) analysis using a two-color reference design system [14,17,32]. Five tadpoles were pooled for each of three biological replicates per developmental stage. Both data and platform have been submitted to the Gene Expression Omnibus (GEO; accession numbers [GEO:GPL10302] for the platform and [GEO:GSE21303] for the data). To identify statistically significant gene expression changes, ANOVA was performed across all developmental time points with a false discovery rate of ≤5% for multivariate correction. Bioinformatics analysis was performed with software from the NIA microarray analysis tool [13,33,34], GenMAPP and MAPPFinder with GO terms [35,36] and GOMiner [37]. To depict gene expression changes using heatmaps, the normalized intensity values at different stages for each gene were mean-centered and analyzed with TIGR-MEV software [38].

### RT-qPCR for amphibian intestines

RT-qPCR was performed as described [32] using primers and either FAM-labeled TaqMan probes or SYBR^® ^Green (Applied Biosystems, Foster City, CA, USA). Genes examined with the FAM-labeled Taqman probes were *TRβ*, *ST3*, *XHH*, *IFABP*, *TH*/*bZIP *and *GelA*. The expression level of each gene was normalized to that of the control gene *rpl8 *(ribosomal protein L8) [39]. Additional genes for microarry validation were analyzed with SYBR^® ^Green with the expression level of each gene normalized to that of the reference gene, *EF-1α *(elongation factor 1α). Primers are listed in Tables S1 and S2A in Additional file 1.

### Intestine histology, TUNEL assay, BrdU treatment, immunohistochemistry and in situ hybridization

Intestines were Bouin fixed, embedded in paraffin, sectioned and stained with H&E. Cell death was detected with TUNEL (terminal deoxyribonucleotidyl transferase-mediated dUTP-biotin nick labeling) assay [40]. To identify proliferating cells, live tadpoles were injected with 10 μl of BrdUrd (10 mM) 12 h before fixation with 4% paraformaldehyde, embedded in OCT compound, cryosectioned and processed for immunohistochemistry as described [41]. *In situ *hybridization probes were generated from cDNA clones (Open Biosystems Inc. Huntsville, AL, USA). The plasmids were linearized to synthesize both sense and antisense probes with T7 or SP6 RNA polymerase by using digoxigenin (DIG) RNA labeling mix (Roche Applied Science, Indianapolis, IN, USA). *In situ *hybridization was done as described [42].

### RT-qPCR for mouse intestines

Mouse RNA samples were purchased from Zyagen Laboratories (San Diego, CA, USA) at the ages of E17, E18, 1 day, and 2, 3, 4 and 8 weeks. These time points were selected to encompass the developmental period of intestinal maturation and when T3 levels mimic those around stages 63 to 66 in *Xenopus*. RT-qPCR was performed with SYBR^® ^Green as described above for amphibian tissue. The expression level of each gene was normalized to that of the reference gene, *RPS13 *(ribosomal protein S13). Primers are listed in Table S2B in Additional file 1.

## Abbreviations

BrdU: 5-bromo-2-deoxyuridine; EF-1α: elongation factor 1α; GelA: gelatinase A; GI: gastrointestinal; GO: Gene Ontology; H&E: haematoxylin and eosin; IFABP: intestinal fatty acid binding protein; rpl8: ribosomal protein L8; RT-qPCR: quantitative reverse-transcription PCR; ST3: stromelysin-3; T3: thyroid hormone; TGF-β: transforming growth factor-beta; TH/bZIP: T3-responsive basic leucine zipper transcription factor; TR: T3 receptor; TUNEL: terminal deoxyribonucleotidyl transferase-mediated dUTP-biotin nick labeling; XHH: sonic hedgehog.

## Authors' contributions

RH, BD, DB, and MF carried out the experiments and data analysis. RH wrote the first draft. YS supervised the research and finalized the paper. All the authors critically revised and approved the final version of the paper.

## Supplementary Material

Additional file 1 **Tables S1 to S6**. A PDF file containing supplementary tables. Click here for file

Additional file 2 **Figure S1**. A PDF file containing a supplementary figure. Click here for file

## References

[B1] Dauca M Bouziges F Colin S Kedinger M Keller MK Schilt J Simon-Assmann P Haffen K Development of the vertebrate small intestine and mechanisms of cell differentiation Int J Dev Biol 1990 34 205 218 2203458

[B2] Cheng H Leblond CP Origin, differentiation and renewal of the four main epithelial cell types in the mouse small intestine. III. Entero-endocrine cells Am J Anat 1974 141 503 519 10.1002/aja.1001410405 4216261

[B3] van der Flier LG Clevers H Stem cells, self-renewal, and differentiation in the intestinal epithelium Annu Rev Physiol 2009 71 241 260 10.1146/annurev.physiol.010908.163145 18808327

[B4] Simon TC Gordon JI Intestinal epithelial cell differentiation: new insights from mice, flies and nematodes Curr Opin Genet Dev 1995 5 577 586 10.1016/0959-437X(95)80026-3 8664545

[B5] Segal GH Petras RE Sternberg SS Small intestine Histology for Pathologists 1992 New York.: Raven Press, Ltd 547 571

[B6] Crosnier C Stamataki D Lewis J Organizing cell renewal in the intestine: stem cells, signals and combinatorial control Nat Rev Genet 2006 7 349 359 10.1038/nrg1840 16619050

[B7] Tata JR Gene expression during metamorphosis: an ideal model for post-embryonic development Bioessays 1993 15 239 248 10.1002/bies.950150404 8517853

[B8] Shi Y-B Amphibian Metamorphosis: From Morphology to Molecular Biology 1999 New York: John Wiley & Sons, Inc

[B9] Shi Y-B Ishizuya-Oka A Biphasic intestinal development in amphibians: embryogensis and remodeling during metamorphosis Curr Top Dev Biol 1996 32 205 235 10.1016/S0070-2153(08)60429-9 8929670

[B10] Buchholz DR Paul BD Fu L Shi YB Molecular and developmental analyses of thyroid hormone receptor function in *Xenopus laevis*, the African clawed frog Gen Comp Endocrinol 2006 145 1 19 10.1016/j.ygcen.2005.07.009 16266705

[B11] Hennings S Rubin D Shulman J Johnson LR Ontogeny of the intestinal mucosa Physiology of the Gastrointestinal Tract 1994 3 New York: Raven Press 571 601

[B12] Leloup J Buscaglia M La triiodothyronine: hormone de la métamorphose des amphibiens CR Acad Sci 1977 284 2261 2263

[B13] Sharov AA Dudekula DB Ko MS A web-based tool for principal component and significance analysis of microarray data Bioinformatics 2005 21 2548 2549 10.1093/bioinformatics/bti343 15734774

[B14] Das B Cai L Carter MG Piao Y-L Sharov AA Ko MSH Brown DD Gene expression changes at metamorphosis induce by thyroid hormone in *Xenopus laevis *tadpoles Dev Biol 2006 291 342 355 10.1016/j.ydbio.2005.12.032 16458881

[B15] Liu X Zou H Slaughter C Wang XC DFF, a heterodimeric protein that functions downstream of caspase-3 to trigger DNA fragmentation during apoptosis Cell 1997 89 175 184 10.1016/S0092-8674(00)80197-X 9108473

[B16] Sancho E Batlle E Clevers H Signaling pathways in intestinal development and cancer Annu Rev Cell Dev Biol 2004 20 695 723 10.1146/annurev.cellbio.20.010403.092805 15473857

[B17] Buchholz DR Heimeier RA Das B Washington T Shi Y-B Pairing morphology with gene expression in thyroid hormone-induced intestinal remodeling and identification of a core set of TH-induced genes across tadpole tissues Dev Biol 2007 303 576 590 10.1016/j.ydbio.2006.11.037 17214978

[B18] Huang MC Chen HY Huang HC Huang J Liang JT Shen TL Lin NY Ho CC Cho IM Hsu SM C2GnT-M is downregulated in colorectal cancer and its re-expression causes growth inhibition of colon cancer cells Oncogene 2006 25 3267 3276 10.1038/sj.onc.1209350 16418723

[B19] Suman G Jamil K Suseela K Vamsy MC Novel mutations of CYP3A4 in fine needle aspiration cytology samples of breast cancer patients and its clinical correlations Cancer Biomark 2009 5 33 40 19242060 10.3233/CBM-2009-0569 PMC12922810

[B20] Pearce CL Near AM Van Den Berg DJ Ramus SJ Gentry-Maharaj A Menon U Gayther SA Anderson AR Edlund CK Wu AH Chen X Beesley J Webb PM Validating genetic risk associations for ovarian cancer through the international Ovarian Cancer Association Consortium Br J Cancer 2009 100 412 420 10.1038/sj.bjc.6604820 19127255 PMC2634713

[B21] Koshikawa N Hasegawa S Nagashima Y Mitsuhashi K Tsubota Y Miyata S Miyagi Y Yasumitsu H Miyazaki K Expression of trypsin by epithelial cells of various tissues, leukocytes, and neurons in human and mouse Am J Pathol 1998 153 937 944 9736042 10.1016/S0002-9440(10)65635-0 PMC1853012

[B22] Ghosh D Porter E Shen B Lee SK Wilk D Drazba J Yadav SP Crabb JW Ganz T Bevins CL Paneth cell trypsin is the processing enzyme for human defensin-5 Nat Immunol 2002 3 583 590 10.1038/ni797 12021776

[B23] Neurath H Evolution of proteolytic enzymes Science 1984 224 350 357 10.1126/science.6369538 6369538

[B24] Shaheduzzaman S Vishwanath A Furusato B Cullen J Chen Y Banez L Nau M Ravindranath L Kim KH Mohammed A Chen Y Ehrich M Srikantan V Sesterhenn IA McLeod D Vahey M Petrovics G Dobi A Srivastava S Silencing of lactotransferrin expression by methylation in prostate cancer progression Cancer Biol Ther 2007 6 1088 1095 17568188 10.4161/cbt.6.7.4327

[B25] Liu F Fan D Qi J Zhu H Zhou Y Yang C Zhu Z Xiong D Co-expression of cytokeratin 8 and breast cancer resistant protein indicates a multifactorial drug-resistant phenotype in human breast cancer cell line Life Sci 2008 83 496 501 10.1016/j.lfs.2008.07.017 18725232

[B26] Choi MY Romer AI Hu M Lepourcelet M Mechoor A Yesilaltay A Krieger M Gray PA Shivdasani RA A dynamic expression survey identifies transcription factors relevant in mouse digestive tract development Development 2006 133 4119 4129 10.1242/dev.02537 16971476

[B27] Stuckenholz C Lu L Thakur P Kaminski N Bahary N FACS-assisted microarray profiling implicates novel genes and pathways in zebrafish gastrointestinal tract development Gastroenterology 2009 137 1321 1332 10.1053/j.gastro.2009.06.050 19563808 PMC2785077

[B28] Muraoka RS Koh Y Roebuck LR Sanders ME Brantley-Sieders D Gorska AE Moses HL Arteaga CL Increased malignancy of Neu-induced mammary tumors overexpressing active transforming growth factor beta1 Mol Cell Biol 2003 23 8691 8703 10.1128/MCB.23.23.8691-8703.2003 14612410 PMC262670

[B29] Bulus NM Sheng HM Sizemore N Oldham SM Barnett JV Coffey RJ Beauchamp DR Barnard JA Ras-mediated suppression of TGFbetaRII expression in intestinal epithelial cells involves Raf-independent signaling Neoplasia 2000 2 357 364 10.1038/sj.neo.7900099 11005570 PMC1550294

[B30] Allen CE Du J Jiang B Huang Q Yakovich AJ Barnard JA Transformation by oncogenic Ras expands the early genomic response to transforming growth factor beta in intestinal epithelial cells Neoplasia 2008 10 1073 1082 18813357 10.1593/neo.07739 PMC2546594

[B31] Vasiliou V Vasiliou K Nebert DW Human ATP-binding cassette (ABC) transporter family Hum Genomics 2009 3 281 290 19403462 10.1186/1479-7364-3-3-281 PMC2752038

[B32] Heimeier RA Das B Buchholz DR Shi YB The xenoestrogen bisphenol A inhibits postembryonic vertebrate development by antagonizing gene regulation by thyroid hormone Endocrinology 2009 150 2964 2973 10.1210/en.2008-1503 19228888 PMC2689811

[B33] Benjamini Y Hochberg Y Controlling the false discovery rate: a practical and powerful approach to multiple testing J Roy Statist Soc Ser B 1995 57 289 300

[B34] NIA Array Analysis http://lgsun.grc.nia.nih.gov/ANOVA

[B35] Doniger SW Salomonis N Dahlquist KD Vranizan K Lawlor SC Conklin BR MAPPFinder: using Gene Ontology and GenMAPP to create a global gene-expression profile from microarray data Genome Biol 2003 4 R7 10.1186/gb-2003-4-1-r7 12540299 PMC151291

[B36] Dahlquist KD Salomonis N Vranizan K Lawlor SC Conklin BR GenMAPP, a new tool for viewing and analyzing microarray data on biological pathways Nat Genet 2002 31 19 20 10.1038/ng0502-19 11984561

[B37] Zeeberg BR Feng W Wang G Wang MD Fojo AT Sunshine M Narasimhan S Kane DW Reinhold WC Lababidi S Bussey KJ Riss J Barrett JC Weinstein JN GoMiner: a resource for biological interpretation of genomic and proteomic data Genome Biol 2003 4 R28 10.1186/gb-2003-4-4-r28 12702209 PMC154579

[B38] Saeed AI Sharov V White J Li J Liang W Bhagabati N Braisted J Klapa M Currier T Thiagarajan M Sturn A Snuffin M Rezantsev A Popov D Ryltsov A Kostukovich E Borisovsky I Liu Z Vinsavich A Trush V Quackenbush J TM4: a free, open-source system for microarray data management and analysis Biotechniques 2003 34 374 378 12613259 10.2144/03342mt01

[B39] Shi Y-B Liang VC-T Cloning and characterization of the ribosomal protein L8 gene from *Xenopus laevis* Biochim Biophys Acta 1994 1217 227 228 8110841 10.1016/0167-4781(94)90042-6

[B40] Fu L Ishizuya-Oka A Buchholz DR Amano T Matsuda H Shi YB A causative role of stromelysin-3 in extracellular matrix remodeling and epithelial apoptosis during intestinal metamorphosis in *Xenopus laevis* J Biol Chem 2005 280 27856 27865 10.1074/jbc.M413275200 15929979

[B41] Schreiber AM Cai L Brown DD Remodeling of the intestine during metamorphosis of *Xenopus laevis* Proc Natl Acad Sci USA 2005 102 3720 3725 10.1073/pnas.0409868102 15738398 PMC553331

[B42] Hasebe T Hartman R Matsuda H Shi YB Spatial and temporal expression profiles suggest the involvement of gelatinase A and membrane type 1 matrix metalloproteinase in amphibian metamorphosis Cell Tissue Res 2006 324 105 116 10.1007/s00441-005-0099-7 16418836

