# Supplementary material for: Studies on Xenopus laevis intestine reveal biological pathways underlying vertebrate gut adaptation from embryo to adult
Source: Genome Biol. 2010 May 19;11(5):R55. doi: 10.1186/gb-2010-11-5-r55 (PMC2898076; doi:10.1186/gb-2010-11-5-r55)
Supplement: Additional file 1 — Tables S1 to S6. A PDF file containing supplementary tables. [file gb-2010-11-5-r55-S1.PDF]

Supplemental Table S1: Primers and probes used in real time RT-qPCR with Taqman assay.

Gene	Forward Primer	Reverse Primer	FAM dye-labeled probe
**THbZIP**	GGACAAGTGAGAAGCAAGAACAAG	GGGTTGGGCAAGGAACAAG	TTCTGCTGCCCATTCAG
**ST3**	CACTTGTAGCCATTGTATCACACTCA	GCCATGATCTTTCTGAGGCTTTTC	ATGCATTCTCACAAGCTGT
**TRβ**	CAAGAGTTGTTGATTTTGCCAAAAAGC	ACATGATCTCCATACAACAGCCTTT	CTGCCATGTGAAGACC
**GelA**	GACGATGATGAGCTTTGGACACTA	GAACTCTCCATCTGCATTACCATACTT	ACAACTTGGCCTTCTCC
**XHH**	CGAGTCCAAAGCTCATATTCACTGT	AGCACCCGCCAGACTTG	CCACTGAGTTCTCTGCTTTG
**IFABP**	AAGGTTGACAGAAGTGAAAACTATGAGAA	CCATCTTGCTGGATTATGACCTTCA	CTTCCGTTTCACTATATTTAC
**rpl8**	AGAAGGTCATCTCATCTGCAAACAG	CAATACGACCACCTCCAGCAA	CAACCCCAACAATAGCT

Supplemental Table S2A: Primer pairs used in qRT-PCR validation of the *Xenopus* cDNA array data. GenBank accession numbers, forward and reverse primer sequences are given.

GenBank	Forward Primer	Reverse Primer
**U41855**	GGCAAGAAAGAGCATGGAGA	CAAAGAGGTTGTATCCTTTATTGTCC
**BC076737**	TTGTCCTGGCAACACGGAAC	TGGCAATCCCTGGTCACTTG
**BC06001**	GGTTTATCACTGGCGGTTACCAC	AGCAGGACGGTGTCTGTTTCTGAC
**BC084618**	CACTGGCTACTCGGCAATGTTG	AAACCATACAGGATAAGCACCACC
**BC072842**	CAAATCTTCAGGGGGTAGCAGC	TGATGATGTCACCACTGGGATG
**BC077065**	AGAGAAACCTGTGGAGTTCCCC	CTCACGCCTTGCTTGTCTTAGTG
**BC054202**	TGTGGTTACGACACGGTGAAGG	TCCAAGGTCTGTTTCCCCATC
**X90838**	CGGTGAAGATGATGACGATGATGAG	GCTAAATCCCCAATAAAGGCTGTC
**BC041213**	GGCTCAGAACATACACTCAATGGA	AAAGACCCCAACCACAGCAAGTCC
**BC056840**	TTGTGTGGATGGAGGCGATGTG	CGTGTCGGTTTCTGTCTGTGAAC
**BX844453**	CACCTGTAGTTGACCTTAGTGGCG	TCTCCCAGTCTGAAGAGGAATGG
**BJ057663**	GACTCCAAATGTTGCCCACGAG	GGAACTGACCTTCAATCCAATGC
**BC078061**	ATGGACAAGTCTTTGCCCACAC	CCCCCAACCAGTGATGTAACAG
**BC081152**	GTGTCCCAGACACCCGTTG	TTGGTAGAACTGCTGGAAGGA
**BX845609**	GGACAAGTGAGAAGCAAGAACAAG	GGGTTGGGCAAGGAACAAG
**BC073555**	GCCTCTTCTCTCCAACACTGAATG	GAACTTCCCCAGGACACAATACC

Supplemental Table S2B: Mouse genes analyzed by qRT-PCR during intestinal development. GenBank accession numbers, HUGO (gene name), forward and reverse primers used in qRT-PCR are listed. These genes are upregulated at metamorphic climax in *Xenopus* intestine. A "Y" in the last column indicates that the gene is upregulated between 1 day and 2 weeks after birth when T3 level rises, thus similar to the regulation during *Xenopus* metamorphosis. Otherwise, the gene is designated with an "N".

GenBank	HUGO	Forward Primer	Reverse Primer	Upregulated after birth
NM_053110	GPNMB	GCTGGTCTTCGGATGAAAATGA	CCACAAAGGTGATATTGGAACCC	N
NM_011415	SNAI2	TGGTCAAGAAACATTTCAACGCC	GGTGAGGATCTCTGGTTTTGGTA	Y
NM_008516	LRRN1	GCACTGGGAAAGCAGCTTG	GTGGACTGTGGGGTAAACCAA	Y
X04770	IGLL1	ACTCTGTTCCTGCCTTCCTTA	GAGGGTTGGGTTGTCTCTACA	N
NM_010128	EMP1	TTGGTGCTACTGGCTGGTCT	CATTGCCGTAGGACAGGGAG	Y
NM_133862	FGG	ACCAGAGATAACTGTTGCATCCT	CCACGTCGGTTTGGTAAGAAG	N
NM_028749	NPL	AAGAAACTCCGGGGTCTTGTT	CCTGTGGTGCCATTCACAAAAAT	N
BC014821	RENBP	GTATTGTCGCTTATACCGCAGT	AACGCAGCAAAAATTCACCAC	N
NM_009243.3	SERPINA1A	TCCCCCTTGGCTCCCATTGCT	GGGAGTCATTTTCAGCTTGCTGTCCC	N
NM_008606	MMP11	CCGGAGAGTCACCGTCATC	GCAGGACTAGGGACCCAATG	Y
NM_008608	MMP14	CAGTATGGCTACCTACCTCCAG	GCCTTGCCTGTCACTTGTAAA	Y
NM_174857	MAMDC	GTGTGGCTTTGACTCCGTGT	GCCTGCAAATCAGAACTCAGC	Y
NM_010401	HAL	CTGTGCGACGCTACATGAAGA	TCATTGTCCTCTAAGGCCACC	N
NM_177794	TMEM26	TTCCTGTTGCATTCCCTGGTC	GCCGGAGAAAGCCATTTGT	Y
X93167	FN1	ATGTGGACCCCTCCTGATAGT	GCCCAGTGATTTCAGCAAAGG	Y
AK089294	CTSD	CCTGGCTTCGTCCTCCTTC	GGCGATGACTGCATGGAGT	Y
NM_021391	PPP1R1A	CAGACGATGGTTGAACATCACC	GGGGGTATCTGGCCTTGAC	Y
NM_007995	FCNA(FCN1)	GGGCCTTTTCAGGACTGCTC	GCCAGGGCAACTTTGGATGA	Y
NM_001145960	SLC37A2	GCGGCAGAAGCAGTGGCTCTA	AGCAGGGGTGGCCCATGTTG	Y
NM_008607.1	MMP13	GCCCATGAGCTTGGCCACTCC	GGGGTCTTCATCGCCTGGACCATA	N
NM_021439.2	CHST11	TGCACCCAGTCATGCGGAGG	CTGTTGGCCCGGCAGGTGTC	Y
NM_028870.3	CLTB	AGCGGGGCTGGTTCAGAGGA	CCTGTCCGCCTGGGCAATCG	N
NM_011594.3	TIMP2	CAGCAGTGTGCGGGGTCTCG	GCCATCTCCTTCTGCCTTTCCTGC	Y
NM_029568.2	MFAP4	GGGCGGCAAGTGGACGGTTT	TCAGCACGGCCAAAGCCCAG	Y
NM_139306.2	ACER2	ATGACAGGGGCAGGTTCAAGGC	GCTCTGCAACAAGCAGCGCA	Y
NM_015766.2	EBI3	TCCGCCAGGTGGGACCCATT	GCCCAGGGAGGCTCCAGTCA	Y
NM_198408.3	CRHBP	AAGCCGCGGTCTACGACCCT	GCATGTCCAGGCACCGCAGA	Y

Supplemental Table S3A: Genes up-regulated during natural development

Note: All the HUGO symbols are derived from predicted human homologs

Stage 58 v Stage 53			Stage 61 v Stage 53			Stage 66 v Stage 53		
GenBank	HUGO	Fold Change	GenBank	HUGO	Fold Change	GenBank	HUGO	Fold Change
BC054155		123.28	AF170337		617.16	BC081272		444.83
BC059976	CD74	32.15	BC054155		487.85	BC072970	ELA1	176.44
BC084607		30.06	Z27093	MMP11	372.22	BC078061		136.11
BC084832	CPB1	28.95	CF548836		236.26	BC081152	NFIX	121.87
BC080035	HLA-DRA	27.97	BC043635	ARG1	159.55	BX849304	ELA1	86.57
BG407150		26.88	BC084379	IGLL1	137.72	BC084428		83.83
L15586		25.01	L15586		129.80	BC082401	DPP4	74.40
BC081091	GBP6	24.68	U37376	MAMDC2	126.33	BC080096	SULT1A1	72.69
BP700722		22.44	BC072277		125.83	BC059786	APOA1	69.65
D13684	HLA-DQB1	22.10	BC087471		105.24	BC084832		60.91
BC084379	IGLL1	21.19	BC081152	NFIX	86.80	AY260728	SLC5A8	58.97
L20725	MR1	18.99	BG160459	HBE1	76.30	BC054155		53.57
BC059315	GBP7	17.81	AJ278067		71.27	BC056856	CPA1	38.45
BX846343	SEPT4	17.54	BC082413	IGLL1	57.74	CF271543	SPINK1	34.55
CX131348	PSMB9	17.01	BC071159	GPNMB	48.76	L28111	DIO3	34.53
BC054271	CEL	16.10	BC084875	IGLL1	48.19	BX846064	PGLYRP1	32.00
BC078585	CELA1	14.89	BC074477	EMP1	45.07	BG160459	HBE1	31.96
BC084831	CPA1	14.35	BC056080	MMP13	44.14	BG346716		31.94
BC056841	AMY2A	13.96	BC077870	MMP14	42.45	CB943692	CRISP3	31.69
BC082413	IGLL1	13.49	BX853254		38.96	BC084270		24.97
CB941299	CCL13	13.38	CB943692	CRISP3	38.17	BC053814	INMT	24.27
BC077420		13.34	BG407150		36.83	AF170337		24.15
BC085042		13.06	BC073180		36.55	BC061680	RDH16	24.15
BC056039	PSMB7	12.75	AF072455	MMP2	32.67	BC059976	CD74	22.40
BC059335	PSMB8	12.62	BC075151	ANXA1	30.94	BC082923	TNNI3	22.26
BC085029		11.71	BC093541		30.05	CF271248	LGALS1	21.19
BC078528	PNLIP	11.59	U08407	ARG2	29.28	CD300904		21.17
Y12326	CD3G	11.34	BX845344	ALDH1A2	29.28	BC072097	ACTC	20.68
BC077477		11.33	L28111	DIO3	28.70	BC085060		20.23
BC054227	CEL	10.91	U41855	C5orf13	27.12	BC043635	ARG1	20.07
BC054987	NAALADL1	10.72	BC053814	INMT	26.90	BC054202	CYP2C8	18.55
U68724	MPO	10.65	BC054947	MMP2	26.47	BC077848		17.23
BC056856	CPA1	10.26	BC077477		26.40	BC084607		16.91
CD255960	VPREB3	10.01	U41858	CRHBP	24.91	BG407150		16.35
BC077883		9.93	BC084607		23.15	BC070682	KRT19	16.04
CF271543	SPINK1	9.56	X61338		22.70	BC054987	NAALADL1	15.98
AF072455	MMP2	9.45	BJ059247		22.27	BC070669	ADH1B	15.66
CF548836		9.20	BC083048	MFAP4	21.68	BC054284	HSD11B1	15.61
BC083045		9.13	BC059976	CD74	21.56	BC073555	CTRB1	14.60
BX852027	CCL5	8.70	BC085060		21.56	BC074179	ABAT	14.42
BC073555	CTRB1	8.51	BC077915		19.56	BC045220	MATN2	12.53
CF283196		8.21	U68724	MPO	19.52	BC056841	AMY2A	12.21
BC074200	HAO2	7.96	BC059789	MAL	19.23	BC085209	FAM55A	11.96
BE508034		7.92	BC073409	C1QB	19.06	BC071004	SULT2B1	11.68
AF062387	ABCB9	7.57	AY260733	PPP1R1A	19.05	BX845609	PRSS2	11.63
BC045044	DSTN	7.49	BC077848		18.64	BX843298	PAFAH1B1	11.44
BC092343		7.03	AY069942	MPO	18.55	BC082713	CDC25C	11.28
BC085064		6.83	U60424	IGLL1	18.37	BC074200	HAO2	11.21
BG037463		6.71	BP692999		17.92	BC078585		10.86
BC077868		6.57	BC068634	TWSG1	17.83	BC080035		10.65

BM261196	IL2RG	6.36	CF283196		17.60	BE509325		10.59
AF440822	PTPNS1	6.28	BC073276	CHIT1	17.00	BX846116	MUC3B	10.45
BC084666		6.24	BC078523	SERPINA1	16.94	BC087465	SNTA1	9.44
CF270416		6.19	BC080035		16.92	BC073444		9.21
BC060433	DEF6	6.18	BC081167	TIMP2	16.52	BC059789	MAL	9.04
BC081221	PSTPIP1	6.06	BC085056	PYCARD	16.50	AF513854	DIRAS1	8.93
CF284976		6.01	CD811314		16.35	BX846582	TNXB	8.87
BC089175	MR1	5.85	CD255960	VPREB3	16.28	BC056039	PSMB7	8.78
Z27093	MMP11	5.84	BC082529	RENBP	15.80	BC074358	PDE6H	8.71
BC054214	SOCS3	5.79	BC085070	HLA-DQA2	15.62	BC043788	OGN	8.64
D44540	PSMB8	5.68	BC080096	SULT1A1	15.59	BC045083	EEF1A1	8.60
BC056035	SLA2	5.41	BC087516		15.19	BC045022	ID4	8.51
BC072984	IRF1	5.41	BC045220	MATN2	14.84	BC060751	SLC7A5	8.45
CK797290		5.28	CK798046		14.44	L15586		8.40
BC073363	PPT2	5.26	BE507589	HAL	14.42	AW635923		8.39
BC077289		5.19	AF368043	SNAI2	14.32	AY278679	FUT3	8.19
BJ092595		5.19	BC045120	GAL3ST2	14.29	BC074149	CYP2J2	8.16
AB060971		5.17	AB060971		14.26	BC077905	ATP2B2	8.13
BC077906		5.16	CD300904		14.20	BF428025		8.07
BC073409	C1QB	5.15	BC054185	FGG	14.01	CX131348	PSMB9	8.03
BC079773	C1R	5.12	BC082935	PPP1R1A	13.92	D13688	HLA-DQB1	8.00
BP687254		5.08	BC071097	ANXA5	13.84	D13684	HLA-DQB1	7.83
BE507817	BTN3A1	5.06	BC061685	CTSD	13.10	CF521081		7.82
BC061685	CTSD	5.01	BC077905	ATP2B2	13.09	BC057715	PCK1	7.82
BE189714		4.94	L39213	SHH	12.90	CK800512		7.82
BC077231	STX11	4.88	BX851570	IL8	12.81	BC084379	IGLL1	7.77
BC082709	CCR2	4.87	BC072841	FN1	12.39	BC084831	CPA1	7.72
BC073318	LCP2	4.86	BC082923	TNNI3	12.33	BC075151	ANXA1	7.64
BC046950	CFH	4.81	X14920		12.31	BC074489	CNFN	7.49
BC078517	RGS1	4.80	BC056040	MMP13	12.29	BC074300		7.49
BC077629		4.76	BX847105	PLTP	12.28	BM191646	SLC2A9	7.48
BC085024		4.70	BC074392		12.28	BC078565		7.48
BC043783	YES1	4.69	BC084358		12.27	BC074146	HAL	7.46
BX844628	EBI3	4.68	CF270408		12.26	CF548836		7.35
BC059984	IRF1	4.63	BC085209	FAM55A	11.99	BC072978	PRSS3	7.32
M58019	MR1	4.59	BX848475		11.70	U67129	AR	7.31
BC070682	KRT19	4.58	U23535	SCNN1A	11.68	CF285661		7.28
BI313803		4.56	AF109923	GLI2	11.64	BG264196	HBA2	7.25
BG018842		4.56	BC077649	KRT12	11.44	BC085064		7.25
BG657077	GPR17	4.55	BC074476	RABL2B	11.33	BC070671	SLC34A2	7.08
BC084974	C1S	4.53	BC056039	PSMB7	11.20	BC056091	GK	6.84
BC075237	C20orf100	4.52	BG264196	HBA2	11.18	BC073634		6.74
BC084875	IGLL1	4.52	BC061680	RDH16	10.97	BC045218	ALDOC	6.72
BC073417	CENTB1	4.52	BC063273	INPP5D	10.97	BE509209		6.65
BC073588	ZNFN1A3	4.48	U75996	EOMES	10.85	BP695201		6.53
BC056055	LCP1	4.45	BX844628	EBI3	10.71	BC080025	ADSSL1	6.43
BC087516		4.45	CF271248	LGALS1	10.59	BC089175	MR1	6.39
BC077287	HMHA1	4.44	BC059292	LRRN1	10.33	BC077984		6.38
BC077941		4.44	BC077883		10.28	BC060364	ABAT	6.37
BX854048	RARRES2	4.30	BC084419	NPL	10.15	BC073490	ALDH1L1	6.34
BC061672	STEAP2	4.29	CB941299	CCL13	10.14	CB941299	CCL13	6.26
BC072976		4.23	BP700722		10.01	BC074113		6.26
CB941351	PALM2	4.22	M58019	MR1	10.00	BC081091	GBP6	6.21
AB091342	FCN1	4.22	BC072970	ELA1	9.97	BX847508	COX17	6.20
BC049174	SLC2A3	4.18	BJ067538	PDE2A	9.93	BC085042		6.18

BC070838		4.16	AF387815	PRICKLE1	9.91	BC077420		6.17
BC084633	TRIM25	4.15	BC077420		9.81	L39213	SHH	6.14
AY069942	MPO	4.14	AB091339	FCN1	9.79	BC074340		6.04
BC085055	ANPEP	4.08	BC085024		9.67	BC077573		6.02
BC080036		4.07	BC045044	DSTN	9.62	BC076742	F2	5.90
BC060360	PNLIPRP2	4.05	BC056117	CHST11	9.54	BC088707		5.85
CF522156	C1orf149	4.04	AY289193	RNPC1	9.51	BC078615		5.79
BC045035	CUGBP2	4.01	BC079830	PIN4	9.51	BC073276	CHIT1	5.78
BC085056	PYCARD	4.01	BC077312	CLTB	9.47	AY152672	SLC26A6	5.75
BC059337	FAM49A	3.99	BC073498		9.43	CK797783	S100A11	5.67
BC084896		3.96	AF549892		9.43	U37376	MAMDC2	5.65
BC068752	SOCS3	3.96	CX131348	PSMB9	9.41	AF072455	MMP2	5.64
CF522012		3.95	BX854048	RARRES2	9.37	BC079804	PNPLA2	5.59
BC059319	ITGB2	3.89	BX853527	WDR52	9.25	BC072092	AQP1	5.57
BC073647	SERPINF1	3.84	BJ061785		9.23	BC046669	SLC38A6	5.54
BC077193	TXNIP	3.84	BC079817	RCSD1	9.22	AF185580	HLA-B	5.48
BC074257	FMNL1	3.81	CB943171		9.09	BC057735	LOH11CR2A	5.42
CF287532		3.75	U41856	DPP4	9.06	BC057722	KIAA1914	5.42
BC061681	HLA-DMA	3.71	BC059335	PSMB8	8.99	BX854048	RARRES2	5.37
BC074116		3.71	AF310007	SALL1	8.93	BJ079142	FKBP10	5.29
CV077928		3.69	L19881	APCS	8.91	BC068634	TWSG1	5.28
AF499689	FCER1G	3.68	U42461	GLI3	8.87	BC074196	ACOT12	5.28
BC078557		3.68	BC081140	BASP1	8.85	AJ278067		5.20
BC073053	CRP	3.66	BC084611	CD44	8.84	BC072154		5.19
BC063273	INPP5D	3.66	BC044329	SERPINH1	8.84	BC084414	BHMT	5.15
BC082687		3.65	BJ096279		8.80	BG018949		5.14
BC077848		3.64	BC076737	CCNJ	8.74	BC085029		5.12
D78003		3.64	BC042235	SLC37A2	8.66	BC072986	HSD3B7	5.09
AY204551	TAP1	3.64	BC056857	SNAI2	8.60	BC070531	UCP2	5.05
BC059329		3.62	BC088693		8.55	CD811314		5.01
BC072365		3.61	BI445881		8.52	CD255960	VPREB3	4.99
BX844666		3.58	BP701161		8.51	BC074477	EMP1	4.98
BX851139		3.58	BC077289		8.44	BC059335	PSMB8	4.96
BC085061		3.57	BC077287	HMHA1	8.44	BC073282	L1CAM	4.95
BC073465	SERINC3	3.56	BC084414	BHMT	8.42	BC087347	TGFBI	4.94
BC082529	RENBP	3.55	D13684	HLA-DQB1	8.38	BC073180		4.93
BC072277		3.52	BC043961	CNTFR	8.33	CD302391		4.91
BC084814	C9orf58	3.48	BC081037	TGIF2	8.30	BC072163	PYGB	4.73
BC092329		3.43	BX852027	CCL5	8.28	BC054247	CYP8B1	4.72
BG023176		3.43	BC074410		8.27	BC084358		4.72
BC073353		3.43	CK805172		8.24	BC074392		4.70
BC073545	CYBB	3.42	BC078467	GMFB	8.23	BC070774	TMCC1	4.68
BC044323	RASGRP2	3.37	AY260728	SLC5A8	8.15	BC046670	PTRF	4.63
BC074173	SLC16A3	3.37	BC080038	TMEM26	8.10	BC061663	HAAO	4.62
AY573848	CDKN1A	3.37	BQ730542		8.09	BC054947	MMP2	4.60
BC073303		3.37	BC057739	RUNX1	8.08	Z14122	LBH	4.56
BX844877	EPPK1	3.35	BC060027	DAB2	8.05	BC082413	IGLL1	4.55
BC054206	OGFR	3.32	BC073337		8.03	BC080378	SLC25A29	4.52
BG019050	PYCARD	3.32	BC053786	ANXA1	8.02	BC086634	JARID2	4.48
BC081059	ATP6V0D1	3.31	BC074489	CNFN	8.02	AB080020	LGALS3	4.48
BC047965	KARS	3.30	CK815995		8.01	BP708540		4.48
BC073340	PSME1	3.26	CN325429		7.98	BC059315	GBP7	4.46
BX847233		3.25	CK797783	S100A11	7.98	BX845344	ALDH1A2	4.44
BC099265		3.24	BC073603	CXCR4	7.97	BC048771	CLDN7	4.40
BC059300	RHOG	3.23	BC085042		7.97	AW199587		4.40

BC078467	GMFB	3.22	BC072806	MAB21L1	7.92	BX854001	L2HGDH	4.39
BC073348		3.22	BC084792	OLFM2	7.89	BX849887	CRP	4.30
BX847105	PLTP	3.21	CB943170		7.87	BC077477		4.30
BC085060		3.20	BC048771	CLDN7	7.86	BC077821		4.23
BC074140	NCF2	3.17	CF522156	C1orf149	7.85	BC077505	GPSM1	4.22
BX850436		3.15	AY495962		7.84	BC053786	ANXA1	4.17
BC042351	GRASP	3.12	BC073545	CYBB	7.83	BC079769		4.15
BP703429		3.11	BC081080	CD37	7.82	BF612748		4.13
U60424	IGLL1	3.06	AB091342	FCN1	7.81	BC073678		4.08
Z14122	LBH	3.05	U24433		7.74	BE505195		4.07
BC044261	PRDM1	3.05	BX842808	ASAH3L	7.70	BC042275	EGFL6	4.06
CD301233		3.04	BC084814	C9orf58	7.68	BX844877	EPPK1	4.03
BJ042255		3.03	BC073530	BIN1	7.67	BC083045		4.00
BC085063	CSF2RB	2.99	CF522012		7.65	BC056036	CLIC5	3.98
BG233711		2.98	BC077279		7.65	U41855	C5orf13	3.98
BC085220		2.97	BC073495	PPP1R14B	7.59	CB942680		3.97
AF055588	MGP	2.95	CF284516		7.59	BC042927	KRT12	3.96
BJ063258		2.94	BC082941	DBN1	7.54	BC060330	F10	3.95
BX847406		2.94	BX851035	C17orf62	7.51	AW200120		3.94
BG407525		2.93	BC043749	FYN	7.49	BC054150	SPARC	3.94
BC078133		2.93	BC082931	CTSZ	7.47	BC082680		3.92
BJ089284		2.91	BJ063258		7.46	BC045120	GAL3ST2	3.92
BC077278	SYK	2.89	BF615988		7.46	BC077308		3.92
BC084419	NPL	2.88	BX843298	PAFAH1B1	7.37	BG811769		3.90
BC073319		2.87	BC073311		7.35	BC077880	PMP22	3.89
CD329863		2.86	BC074257	FMNL1	7.33	U68724	MPO	3.88
AW643154	ALLC	2.86	BC084391		7.33	BC070672	CAV1	3.86
CV079029		2.84	BC081091	GBP6	7.30	BC085056	PYCARD	3.85
BC083048	MFAP4	2.83	BG657077	GPR17	7.28	BP685974		3.84
CF521258	PLAC8	2.82	BC047254	CUEDC1	7.25	M88105	LGALS1	3.83
BC073229		2.82	AF310008	YRDC	7.24	M23237	PDGFA	3.82
BC077285	CTSS	2.82	BC084972	ZFHX1B	7.24	BC072806	MAB21L1	3.81
BG729801		2.81	BC088707		7.22	BC088693		3.80
BC044330	SLC16A3	2.81	BC076742	F2	7.20	BC084347	PDE4B	3.79
BX849309		2.81	BC077925	PFC	7.18	BC045044	DSTN	3.77
BP687360		2.80	BC084924	C9orf19	7.17	BC072100		3.75
BC097600	FSCN1	2.80	CB558803	CAT	7.13	DR717081		3.73
BC086466		2.79	BC081059	ATP6V0D1	7.10	BC057717	SEMA3F	3.72
BC077701	FCHO1	2.78	BC045262	HOMER3	7.09	CK799264	CST3	3.72
BX845355	DOCK10	2.78	CF285606		7.07	BC071097	ANXA5	3.72
BC073276	CHIT1	2.77	BC077906		7.05	CB558803	CAT	3.72
BF428058	KARS	2.76	BC070670		7.04	BJ625984	ABCB4	3.72
BC087295	CMTM3	2.75	BC059999	DAB2	7.04	BC074204	CYP2A13	3.71
CK804709		2.74	BC097600	FSCN1	7.02	BC043850	ACSBG2	3.69
BC073321		2.73	BC086634	JARID2	6.98	BC082952	PYGL	3.69
BX842824	NAALAD2	2.73	BC078565		6.97	AF508961	LRP6	3.68
BC077925	PFC	2.71	BC071004	SULT2B1	6.94	BC060497	HSD17B6	3.68
BC060434	CTTN	2.69	AY043258	PPP2R3A	6.93	CF522156	C1orf149	3.68
BC061653	ACY1	2.68	AF287147	LEF1	6.88	D44540	PSMB8	3.67
BC090055		2.68	BC059995	CPN1	6.87	BC081121	INPP5A	3.66
CD361037		2.67	BC087372		6.83	CK797196		3.63
BC084787		2.65	BC056055	LCP1	6.83	BC072168	CEBPA	3.62
BC045059	ARL5B	2.65	BE189714		6.83	BC084217		3.62
BC046657	PTAFR	2.62	BC075160	SULT1C1	6.77	BC081241		3.60
BC084606	RAB37	2.61	AF131890	NGFR	6.76	BC043737		3.60

BC080034		2.61	BX849223	ATCAY	6.76	BC081080	CD37	3.59
BC079817	RCSD1	2.60	AY318877	HUNK	6.73	BC097600	FSCN1	3.57
BC074424		2.59	Y12326	CD3G	6.73	BC074306	HINT3	3.54
BC072174		2.59	BM261196	IL2RG	6.69	BX853459	HAGH	3.54
AF061727	SERPIND1	2.59	BC085063	CSF2RB	6.67	AF033110	FZD5	3.54
BC057722	KIAA1914	2.58	BC084912		6.66	AW640423		3.53
BX845723		2.58	BC074113		6.57	BC056849	CTRB1	3.53
BC046837	P2RY4	2.56	CF288442		6.57	BC077551	ARNTL	3.52
BC044008	MST1	2.56	BJ074424		6.55	BC076806	HSPB7	3.52
BX842732		2.56	CF284349		6.55	BC077910	GLUD1	3.52
BC082936		2.55	BC070838		6.54	BC077883		3.51
BQ736298		2.55	DQ096892		6.53	BC059294	A2M	3.51
BP683068		2.54	CV077928		6.50	BC084974	C1S	3.50
BJ045539		2.53	BC081241		6.48	BX851652	TRIM16	3.50
BC041721	CAMK1	2.53	BX848644	CD38	6.48	BC073316	TRIB1	3.50
BC070719	VPS24	2.52	AF062387	ABCB9	6.47	BC055989	BACE2	3.49
BE680067		2.52	CF548599	GNGT1	6.44	BC078056	SELENBP1	3.47
AY686699	EVL	2.52	BC080392	SEMA3D	6.43	BC092343		3.46
BC079972	TCF7L2	2.52	BC055959	BMP7	6.41	BC082930		3.45
CK798046		2.52	BC056081	MXRA8	6.35	BC049287	COL1A2	3.45
BC077564		2.50	BC087347	TGFBI	6.34	AY069942	MPO	3.45
CX134775		2.50	BC082472	GPBAR1	6.34	BC054215	ELA1	3.45
BX850255	TPRKB	2.50	BC076858		6.32	BC082364	AXIN2	3.44
BC082414		2.50	BC082674	CLDN19	6.32	BC078533		3.44
CD328776		2.49	BC085226	GLT25D1	6.31	BC073409	C1QB	3.43
BC084360	CD53	2.49	BC077938	CBFB	6.31	BC080392	SEMA3D	3.43
BC077535		2.49	BG364947	PRSS35	6.29	BC077535		3.41
BM180861		2.48	BC085223	VIM	6.28	AW871732		3.41
D29796	CFB	2.48	AB087137	ROR2	6.27	BC044027	SFXN1	3.41
BC078136	MMP17	2.47	AF232672	FKBP10	6.26	CK800673		3.40
AJ345114	GRM1	2.46	BC068658	ZNF423	6.23	BX849057		3.39
BC077902	CYBA	2.46	X61349	LOC90925	6.22	BX844938		3.38
CD326347	LENG8	2.46	BC075148		6.22	BC054248	FTCD	3.38
BC088682		2.46	BC060433	DEF6	6.21	U12683	ITGA5	3.37
BX853374		2.46	BC068806	FUT10	6.20	BC060753	COL3A1	3.34
BC068952	EML2	2.43	AF002983	CDH11	6.20	BX852027	CCL5	3.33
AB061672	MBD2	2.42	CD302391		6.18	BC073607	CYP2D6	3.32
BC081243		2.42	BC072252	CTSD	6.17	BC059986	SNX22	3.31
BC084386	SCAP2	2.42	BC061654	PLAT	6.16	BC056126	SH3BGRL2	3.31
BC088746		2.42	AW148133	C22orf3	6.16	AF432354	P2RY1	3.29
U37376	MAMDC2	2.41	BC074424		6.16	BC078467	GMFB	3.28
BC084819	FGL2	2.41	BC077278	SYK	6.16	BC082673		3.27
BC071089	NAGA	2.41	BC073279		6.14	BC088952	TRAM2	3.26
BC073279		2.41	BC044030	TUBB2B	6.13	Y12326	CD3G	3.25
BC073490	ALDH1L1	2.40	BC088682		6.12	BJ092482	RHBDL7	3.25
BI312781	ADAMTS13	2.40	AY310397		6.12	AB034701	COL1A1	3.24
BC087471		2.39	BC073053	CRP	6.11	BC074140	NCF2	3.24
CF284349		2.38	BC082673		6.11	Z27093	MMP11	3.24
BC084611	CD44	2.37	BC041758	H1FX	6.10	BG346671		3.24
BC081080	CD37	2.36	BC082709	CCR2	6.09	BC072277		3.23
BP683892		2.36	BI347717	HBE1	6.09	BC081275	PLS3	3.23
BC045043	ARPC1B	2.36	BC086475	TCF4	6.07	BJ069449	MSRB2	3.22
BC073603	CXCR4	2.35	BJ060214		6.07	BC092031		3.22
BX850413		2.35	BC044963	CXCR4	6.06	U41858	CRHBP	3.22
BC054295	TSPAN8	2.35	BC081189	PLTP	6.05	BC060328	CYP2C8	3.20

AF549892		2.33	BC080377	APCDD1	6.04	BC082462	CAPNS1	3.18
CB560728		2.33	BF615812	HBE1	6.03	AY289193	RNPC1	3.17
BG017658	PSD4	2.32	BI313803		6.03	BC073545	CYBB	3.17
AF287147	LEF1	2.32	BC082687		6.03	BX847105	PLTP	3.16
CV079892	EMR3	2.32	BC060435	HMOX1	6.01	BC077925	PFC	3.16
BC041758	H1FX	2.32	BX853189	HOXC5	6.01	CD302358	COL6A3	3.15
BC048771	CLDN7	2.32	BC084307		6.01	BC097581		3.15
BC060330	F10	2.31	BC061653	ACY1	6.00	BC077870	MMP14	3.14
BC057735	LOH11CR2A	2.30	BC043838		6.00	BC060433	DEF6	3.14
BC080063	RFFL	2.30	BC077286		5.99	BX853433		3.13
BC073402		2.30	BC045050	MYO5A	5.99	BC073363	PPT2	3.13
BC073550	ACP5	2.29	BC044261	PRDM1	5.99	BP700722		3.12
BC072062	ACP5	2.29	BC082432	TCF12	5.99	BC089134		3.11
X52692	ETS1	2.29	BC072092	AQP1	5.98	BC074257	FMNL1	3.09
BC075161	ETS1	2.27	AF170339	C18orf26	5.97	CX131041		3.08
BC061649	SNX5	2.26	BC041207	MARCKS	5.96	CX131946		3.07
CF270696		2.26	BX842824	NAALAD2	5.95	BC084318	ATP6V1G3	3.07
BC073525		2.25	BC078136	MMP17	5.94	BC077330		3.07
BG161028		2.25	BC080054	LDHB	5.93	AF529219		3.06
BC074410		2.24	BX853433		5.92	BG233646	S100A4	3.06
BC073396	FNBP1	2.24	BC045218	ALDOC	5.91	BC087471		3.04
AY043258	PPP2R3A	2.23	M18350		5.91	BC077275	RGS5	3.03
BC041207	MARCKS	2.23	BC073363	PPT2	5.91	BC044086	ZFP36L2	3.02
BG161387		2.22	BC084666		5.90	BC073647	SERPINF1	3.02
BI446136	ARHGEF16	2.22	BC089175	MR1	5.90	BC060465	HSD17B6	3.01
BC070796	TMEM30B	2.21	D78003		5.87	CF286277		3.01
BC056059	CTSS	2.21	BC088957	CHST12	5.87	BC077468	CAV2	3.00
BX844938		2.20	BF612692		5.86	BC084611	CD44	3.00
BC070713	TRAF2	2.20	BC073319		5.85	BC054257	LDHB	2.99
BC077873	C9orf19	2.20	CB945344		5.85	BC081189	PLTP	2.99
BC046669	SLC38A6	2.19	BC073318	LCP2	5.84	AW200470	EMP2	2.98
AY035397	SOX9	2.18	AF549913		5.84	BG161387		2.98
BC068656	RCBTB1	2.18	AF302765	PTCH	5.83	CF284976		2.97
BC079826	MR1	2.18	BC046669	SLC38A6	5.83	CF270696		2.97
BC079918	FAM107B	2.18	BC077285	CTSS	5.82	BC073318	LCP2	2.96
U75996	EOMES	2.18	BC078061		5.81	BC084972	ZFHX1B	2.95
BX845117	P2RY5	2.17	BC044689	CTSB	5.80	AF387815	PRICKLE1	2.95
BC083025	PLEC1	2.17	U12683	ITGA5	5.77	M58019	MR1	2.95
BX850727		2.17	BG022582	IGJ	5.77	U41856	DPP4	2.95
BC042348	FAM49B	2.17	BC082399		5.75	BX847406		2.94
BJ066554		2.17	BX849077		5.72	CD329863		2.94
BG023179		2.15	BE508825	VSIG4	5.72	BC054234	GCG	2.93
BX851469		2.15	BC043948	PDGFRA	5.71	BC084792	OLFM2	2.93
BC046671	SPIB	2.15	BC078517	RGS1	5.71	BC082467		2.93
AW200487		2.14	BC045035	CUGBP2	5.70	BC077498		2.92
BC073444		2.14	BF611398	UGCGL1	5.69	BJ081305		2.92
AW200023	PLK3	2.13	CD328137		5.68	BC073254	FHL3	2.91
BC080997	PSAP	2.13	BC060353	CFH	5.67	BC092158		2.91
BC076776	PLEKHA1	2.13	BC073367		5.65	BC061654	PLAT	2.90
BC043850	ACSBG2	2.12	BG161387		5.65	BC045013	SPARC	2.89
BQ384402		2.12	BX844877	EPPK1	5.61	CV079516	SPINK2	2.89
BC057739	RUNX1	2.12	BC072914		5.60	CF521166		2.89
CK798694		2.12	AY262358	CD99	5.59	BC098967	ADD3	2.89
BC072814	CYFIP2	2.11	BC077355	SNN	5.59	BG553365		2.88
BC084840	ST3GAL6	2.11	BC073254	FHL3	5.58	BC063273	INPP5D	2.88

BF072051		2.11	BC072062	ACP5	5.58	BC073465	SERINC3	2.88
CD301800		2.11	BG022421		5.58	BC043783	YES1	2.87
BC077840	RHOV	2.10	BC092325		5.57	BC073498		2.87
BX843871	STYK1	2.10	CB941351	PALM2	5.55	BC041721	CAMK1	2.85
BC073600		2.10	BC059300	RHOG	5.54	BC077868		2.85
BC043749	FYN	2.09	BC079829	DNAJB1	5.54	CK807007		2.85
BP715593		2.09	BX850685		5.53	BC071021	CYR61	2.84
CV076058		2.09	BC043779	SLC16A1	5.51	AF353715	KLF4	2.84
BC077761	FGFR3	2.08	AF187864	MARCKSL1	5.48	BE576052		2.84
BC074357	PERP	2.08	BC080062		5.47	BC081140	BASP1	2.83
BC072796	CASP6	2.07	BG018842		5.46	BE189714		2.83
CD302319		2.07	BC059319	ITGB2	5.46	AF427862	PECAM1	2.82
AY504996	C20orf32	2.07	CD303201		5.45	BC072059	SLC44A2	2.81
BC059983	SIAE	2.07	BC059329		5.45	CK797591		2.81
BC077938	CBFB	2.07	BC077629		5.45	CD327200		2.80
CF271689		2.07	BC085022	NHS	5.44	BC072274		2.80
BC077916	SOAT1	2.06	AF499689	FCER1G	5.43	AF374473	LMO2	2.79
AF465789	TLL1	2.06	BC081240		5.43	BC055959	BMP7	2.78
AW199362	CLK2	2.05	CD301233		5.41	CD099362	LMAN1	2.77
BC073582		2.05	BC076844		5.41	BE507817	BTN3A1	2.77
BF047391		2.05	BC054161	VIM	5.40	AF044080	NCOA3	2.77
BC046372	JMJD2B	2.04	BC044269	CALCRL	5.39	BC084896		2.77
BC071132	CD9	2.04	CF286277		5.39	BC072814	CYFIP2	2.77
BC073351		2.04	BC081221	PSTPIP1	5.38	BC082647		2.77
BJ619086		2.04	CK798617	COX4I2	5.38	AW634666		2.76
BC045050	MYO5A	2.04	BC086271	CPT1B	5.38	BC075237	C20orf100	2.75
BQ397842		2.04	BG018769	LFNG	5.38	BC077221	ACTC	2.75
BC054232	SERPINB6	2.03	BC045011	PARVB	5.37	BC076721	FGF13	2.74
BJ090358		2.03	BC045059	ARL5B	5.37	BC082817	MYADM	2.74
CK798368		2.03	AY660871		5.36	BC072084	FREQ	2.74
BC084073		2.03	BX847588	MARCH3	5.35	CD253705		2.74
BC068947		2.02	AY616034	MYO10	5.35	BC060027	DAB2	2.73
BF612190		2.02	BG017842		5.34	BC074103	DVL1	2.73
AB111447		2.02	BC073229		5.33	BE505753		2.73
BG346398	IREB2	2.01	U37375		5.33	BC073628		2.73
AW200581		2.01	BC045244	COL9A1	5.32	BC084388		2.73
BP688919		2.01	BC054150	SPARC	5.32	BC081228		2.73
BP691572		2.01	BC059320	HHEX	5.32	BC072272	CD81	2.73
CF285606		2.01	BC059285	TBX2	5.32	BC073417	CENTB1	2.72
BC073546		2.01	BC077505	GPSM1	5.31	BC072901		2.72
BC068851	WBP11	2.00	BX851196		5.31	AY495962		2.71
BC070857	SLC36A1	2.00	CB942680		5.31	BC072818	SLC41A1	2.70
BC054257	LDHB	2.00	CV079029		5.29	BJ083148		2.70
BF427961		2.00	BC068972		5.28	BC047255	COL6A1	2.69
BG022428		2.00	M35362	THRB	5.27	BC077854		2.69
CF285027		1.99	BC084768	KCTD12	5.27	AF274053	KCNMA1	2.68
BG360221		1.99	BC078057		5.26	BC041758	H1FX	2.68
BC092033	GPA33	1.99	BX846825		5.25	BC073353		2.68
BC074326		1.99	BP740062		5.25	Z47557	CCK	2.67
BC054234	GCG	1.98	BC042309	H3F3A	5.22	BC082674	CLDN19	2.67
U41856	DPP4	1.98	BP682601		5.22	AW644252		2.67
BE508394		1.97	BC070565	RCOR2	5.20	CD099943		2.66
BC068616	RAB24	1.97	CF270488		5.19	BF615988		2.65
BC077292	COL9A3	1.97	BC073550	ACP5	5.18	BE508034		2.65
BC059981	PLD3	1.97	BC059315	GBP7	5.17	CB563548	TMSL3	2.65

BC077994		1.96	AF283562	LEFTY1	5.16	BC084962	ATP2A1	2.65
BQ723998		1.96	BQ736298		5.16	BC073530	BIN1	2.65
BC097728	AGTRAP	1.96	BC086468	C10orf38	5.14	BC084775		2.65
BC072059	SLC44A2	1.96	BC084974	C1S	5.12	BC073396	FNBP1	2.64
BQ731671		1.96	BC072814	CYFIP2	5.11	BC077906		2.64
BG161758		1.95	BC084775		5.11	BC084266		2.64
BC044717	STAT3	1.95	BC070547	HES1	5.10	BJ067538	PDE2A	2.64
BP688269	SUB1	1.95	Z14122	LBH	5.10	BJ627001	PRX	2.63
CK798118	SH3BGRL3	1.95	BC079972	TCF7L2	5.08	BC081023	EDG4	2.63
AF432354	P2RY1	1.95	BE508034		5.08	BC082612	SLC37A3	2.63
CK800212		1.95	BC084198	NRP1	5.07	BC046671	SPIB	2.63
BM928954	SLAMF8	1.93	CK796806		5.07	BC081221	PSTPIP1	2.63
BC080021	ASAH1	1.93	BC082892		5.07	BC081153	RBPMS2	2.62
CB558803	CAT	1.93	BM928998		5.06	BC083025	PLEC1	2.62
BC084356		1.93	BC098974	CMKOR1	5.05	BX845304		2.62
BM928982		1.93	AJ311059	SMAD3	5.04	BM261196	IL2RG	2.62
BC090219	TMBIM4	1.92	BC084923	GNAI2	5.04	BX852154		2.62
BJ094364		1.92	BX846064	PGLYRP1	5.04	BC072984	IRF1	2.62
BC077570	JAK1	1.92	BC080012		5.03	BC077761	FGFR3	2.62
M58576	ANXA2	1.91	BC082364	AXIN2	5.03	BC074344	RAB25	2.61
BC060396	ELMO2	1.91	BC054257	LDHB	5.03	BC081123		2.61
BC074168	NEU1	1.90	BC072304	TGM2	5.02	BM928982		2.61
BG234189	ARHGAP27	1.90	BJ039688		5.02	AW765923		2.60
J05180	TGFB1	1.89	BC060430	PPAT	5.02	BC045265	CYB5R3	2.60
BC081167	TIMP2	1.89	CF286572		5.02	BX850563	CLDN2	2.60
BC078138		1.89	AB037688	PTCH2	5.01	CF548327		2.60
CD327121		1.89	BC078557		5.01	BG016213	CDX2	2.59
U60093	RBPSUH	1.88	BC073282	L1CAM	5.00	BC054206	OGFR	2.59
BC081140	BASP1	1.88	DR720479		5.00	CV077028		2.59
BC087297		1.88	BC074403		4.99	CD303201		2.58
BP741772		1.88	BC074353	PHACTR1	4.99	U47622	FGF9	2.58
BC060435	HMOX1	1.87	AB086020	SOX4	4.99	BC073295		2.57
BC072916	RNPEP	1.87	CF521258	PLAC8	4.98	CX134775		2.57
CD252706		1.87	BC060754	CORO1A	4.97	BC084886	RGS4	2.57
CB945344		1.87	J05180	TGFB1	4.96	CF548888		2.57
CF287379		1.87	CD325847	FAM62A	4.96	BC079803	ELL	2.57
CF546720		1.86	BE509179		4.95	BC077941		2.57
BC097538	PTPN6	1.86	BX847406		4.95	BC054997	EFNA3	2.56
BC078012	RASGRP2	1.86	BC087295	CMTM3	4.95	BC073337		2.56
BC072847	SOCS1	1.86	BC042351	GRASP	4.94	BC072779	EHD2	2.56
BC087391	SCPEP1	1.85	BC081276	NEFH	4.92	BC077584	SUOX	2.56
BG439828		1.85	CD302319		4.92	BC080063	RFFL	2.55
BC056111	CMTM7	1.85	AJ001754	FZD3	4.92	BC061674	GPR146	2.55
BC044963	CXCR4	1.84	BC074418	CBX1	4.92	BC049174	SLC2A3	2.55
BC076737	CCNJ	1.83	BC073236		4.92	BC082904		2.54
BG438924	PTK2	1.83	BC084386	SCAP2	4.91	BG018842		2.54
BG553375		1.83	BF615463		4.91	BC079815		2.54
CD328147		1.83	BC092329		4.90	BC073582		2.53
BG020384	C17orf37	1.82	BC077380	DACT1	4.90	AY689185		2.52
BG410073	WDR41	1.82	BG159957	ZNF484	4.90	CB983966		2.52
BC077575	DYX1C1	1.82	BC077231	STX11	4.89	BC059295	TFCP2	2.52
BC082952	PYGL	1.82	BC078114	CCDC69	4.89	CF271359	SON	2.52
AF081803	HCK	1.82	BX851139		4.88	BC084215		2.52
BC074476	RABL2B	1.81	BC068920	RND3	4.87	BM180994	RAB15	2.52
BC082848		1.81	D29796	CFB	4.86	BC073279		2.52

BG161509		1.81	CB983966		4.85	BJ059247		2.51
CF271104	TOR1A	1.81	BG407525		4.85	BX847376		2.51
CA982195		1.80	BC073588	ZNFN1A3	4.85	CK797112		2.50
BJ085940		1.80	BX853616		4.84	BC072890	ANXA3	2.50
BC089148	PRSS16	1.80	BX848215		4.84	BC087481		2.50
BX854219	DNAJB1	1.80	BP703429		4.83	BC077231	STX11	2.50
BC084374	BTN2A1	1.80	BM928982		4.82	BE026509		2.49
AF212298	SEPT2	1.79	BC060330	F10	4.82	AF317656	AKT1	2.49
BC068634	TWSG1	1.79	BQ730854	HINT3	4.82	AJ278065	AGXT	2.48
BJ638911		1.79	BC074268	C18orf43	4.82	BC083037	GRHPR	2.48
BC077620	WDR37	1.78	BC072059	SLC44A2	4.81	BC088676		2.48
BG016865		1.78	CF287379		4.81	BC044008	MST1	2.47
BX847588	MARCH3	1.78	BC063737	MLSTD2	4.80	BC063731	HS3ST1	2.47
BC048225	PDCD4	1.77	BC077868		4.80	CF548599	GNGT1	2.47
BM928998		1.77	BC084243	EOMES	4.79	CD301233		2.47
BQ385449		1.77	AF286645	HAND2	4.77	BC059984	IRF1	2.46
CX135390		1.77	AF139165	FZD2	4.77	BC073343	ITGB3	2.46
BX852957		1.75	BC070682	KRT19	4.77	BC047961	FAM3D	2.46
BC082477	PTK9L	1.75	AY152672	SLC26A6	4.76	BC084736	SULT1C1	2.46
BG023328		1.75	BC099265		4.76	BC061670	CCNI	2.46
BC075169		1.75	X66979	FLI1	4.76	BC072287	PCK2	2.45
BQ733387	GRM2	1.74	BE669221	PCGF3	4.76	CF284196		2.45
CK798446		1.74	BG016174		4.74	CF285062		2.45
AF142632	NRG1	1.74	BC060751	SLC7A5	4.73	BC082896		2.45
BF610645		1.74	BC089138	ENPP2	4.72	BC077255	MAFB	2.44
BJ629598		1.73	BQ383675		4.71	BP704578		2.44
L20728	MR1	1.71	BC072356	ZDHHC2	4.70	BC056033	SLC25A24	2.44
L20728	1.71		BM180861		4.70	BC056035	SLA2	2.44
			BC056126	SH3BGRL2	4.68	BM180861		2.44
			BX846575		4.67	CD326197	SULT1C1	2.43
			BC089264		4.67	BC089181	COL6A2	2.43
			BC044323	RASGRP2	4.67	BC043746	SLC19A3	2.43
			BC073642	AASS	4.66	BX853254		2.43
			CX132045		4.66	BC072821		2.42
			BC074300		4.65	BC068899	XPNPEP1	2.42
			CF284976		4.65	BC083015	SIGLEC12	2.42
			CB944397		4.65	BC086274		2.42
			BC073417	CENTB1	4.64	BE680067		2.42
			BC073465	SERINC3	4.64	BC082614	POPDC2	2.41
			CA971861	CSRP2	4.64	BX847233		2.41
			BC084819	FGL2	4.63	AW645021		2.41
			BC080040		4.63	CD326347	LENG8	2.41
			BC045134	SRC	4.63	BC085039	CRIP1	2.40
			BC046716	ATP1B3	4.62	BC076777		2.40
			BJ030986		4.61	BC084924	C9orf19	2.40
			Y13069	DPYSL3	4.60	AJ001754	FZD3	2.40
			AW640423		4.59	BC078079	RHBG	2.40
			U41824	MMP13	4.59	BC085024		2.40
			BC070801	QKI	4.59	BG037463		2.39
			BG729801		4.58	BC066120		2.38
			BC049174	SLC2A3	4.58	BC077891	ITGA2B	2.38
			BC087481		4.57	BC080009		2.38
			AB191720	TSC22D1	4.57	BF232175	ZNF21	2.38
			BC073678		4.56	BJ035117		2.38
			CF287532		4.56	CF289954	MXRA7	2.38

			BC073301	TSPAN18	4.55	BX848252	RASEF	2.37
			CD361577		4.55	BC045030	DYNC1LI1	2.37
			BC074346	CCDC50	4.54	BC077287	HMHA1	2.37
			CK800382	IFITM2	4.54	BC082471		2.37
			BC049389	EDG2	4.53	BC084889		2.37
			BC044024	MEIS2	4.53	BC072158	RAB27B	2.36
			CF270416		4.52	BE505183		2.36
			BC088915	RXRG	4.52	BX850233		2.36
			BX844666		4.51	CK798118	SH3BGRL3	2.36
			BE680067		4.51	BC074252		2.36
			BQ399739		4.50	BC080388	EMILIN2	2.36
			BC084633	TRIM25	4.49	BC077916	SOAT1	2.36
			BC080099	CD276	4.48	BG160621		2.36
			BC073600		4.47	BC042252		2.36
			BX849309		4.47	CD328101		2.35
			BG553375		4.46	BC077332	B3GNT5	2.35
			BC056059	CTSS	4.45	BQ735436		2.34
			BC072084	FREQ	4.45	BC077902	CYBA	2.34
			U19618	FGB	4.45	BC079798	QSCN6L1	2.34
			BC068900	TPP1	4.45	BC063737	MLSTD2	2.34
			BC077902	CYBA	4.45	AB015205	CNTN1	2.33
			BC073494	SOX4	4.44	BC073243		2.33
			BC073396	FNBP1	4.44	BC041207	MARCKS	2.32
			BP687254		4.44	BC043760	ALPL	2.32
			BC078615		4.42	BC078021		2.32
			BX847839		4.42	BC086282	CREBBP	2.32
			BC060434	CTTN	4.42	BC070719	VPS24	2.31
			AY573848	CDKN1A	4.41	BE679528		2.31
			BC074116		4.40	CF548303		2.31
			BC084217		4.40	CK797424		2.31
			BC055998	AGTRL1	4.40	BC073495	PPP1R14B	2.31
			BC044687	SFRP2	4.39	BC073588	ZNFN1A3	2.31
			BC092158		4.39	CD099720		2.31
			BC078133		4.38	AY714078	RUNX1T1	2.30
			AB014611	FOXD3	4.38	BC079773	C1R	2.30
			BC071059	ALDH1A2	4.38	BC081059	ATP6V0D1	2.29
			BC074421	MAB21L1	4.38	CF521264	METTL1	2.29
			BC076827	NT5DC2	4.38	BC054282	LUM	2.29
			BX848927		4.38	BC087410		2.29
			CK798028		4.37	CD326941	PTGS2	2.29
			BG439844		4.36	BC080997	PSAP	2.28
			CD329863		4.36	BE509211		2.28
			AW643154	ALLC	4.36	AF465789	TLL1	2.28
			BC074173	SLC16A3	4.36	BG513927		2.28
			BX845693	PDGFRA	4.35	BC077873	C9orf19	2.28
			AF461119	ROBO1	4.34	BX852516	FAM55A	2.27
			BX848480		4.34	AY280863	CTDSPL	2.27
			BC077873	C9orf19	4.33	BJ096279		2.27
			BC072984	IRF1	4.31	AY573378	MMP15	2.27
			BG578993		4.31	BP713744		2.27
			BX845902		4.30	CB560198	MUC2	2.27
			BC054260	ADH1B	4.29	BC076737	CCNJ	2.27
			BC080034		4.29	BC092027		2.26
			BX845568	BAX	4.28	AW643154	ALLC	2.26
			CB560268		4.28	BC060745	EPHA2	2.26

			D38175	PLXNA1	4.28	BC073043	PBP	2.26
			BC068760		4.27	CK798617	COX4I2	2.26
			BC070797	RND3	4.27	BX848085		2.25
			BC071046		4.27	AJ243591	PCBP2	2.25
			BE131768		4.27	BJ092595		2.25
			AY030051	SEMA3A	4.27	BX844666		2.25
			BC073303		4.27	AB008225	SLC5A11	2.25
			BC086287		4.26	BC060361	TKT	2.24
			BC084356		4.26	BJ640299	RHOB	2.24
			BC074326		4.25	BX853414	MYO18A	2.24
			BC084961	ARHGAP26	4.25	BC072967		2.24
			CB943089	C20orf172	4.25	AB114053		2.23
			CK798118	SH3BGRL3	4.25	BI313803		2.23
			BC057721	INA	4.23	BG811389		2.23
			BC073488	BCAM	4.23	BC054951	EHD2	2.23
			BC080997	PSAP	4.23	BX853192		2.23
			AJ242680	FOXF1	4.22	BC077900	MYL9	2.22
			BC074121	GGCX	4.22	BC077738	PURA	2.22
			BX844667	KLF13	4.21	BC082936		2.22
			BC077798	HPCAL1	4.20	BC073285	RARRES1	2.22
			AJ605777		4.19	BC080012		2.22
			BC068655		4.19	BC043948	PDGFRA	2.21
			AF374473	LMO2	4.17	BC088671	WDR71	2.21
			BX852372	LLGL1	4.16	BC073229		2.21
			AW638861		4.16	BC079762		2.20
			X65943	FGFR2	4.15	CK797077	PRDX5	2.20
			X83962	TMEFF1	4.14	BC090231	GPC4	2.20
			BC044121	DOK4	4.13	BC085026		2.19
			BQ733689		4.13	BX853583		2.19
			BC073257	TSPAN4	4.12	BC061681	HLA-DMA	2.19
			BC080382	BMP1	4.11	BC082856	AXUD1	2.19
			BC045209	PRPH	4.11	CF520724	SERINC2	2.19
			BC073628		4.11	BC074194	GPT2	2.19
			BC079773	C1R	4.10	BQ734261		2.19
			BC073321		4.09	BC088682		2.19
			BC043788	OGN	4.09	BC073340	PSME1	2.18
			BX844554	OVCH1	4.09	BC092325		2.18
			BC046742	NT5DC2	4.08	BC074144		2.18
			BP701946		4.08	BC047258	GCAT	2.18
			BC082395	PPP1R14A	4.07	BC072308	SLC25A11	2.17
			CF520724	SERINC2	4.07	BC077824	CALU	2.17
			BC049287	COL1A2	4.07	BC083041		2.17
			BC070668	APP	4.06	BJ068686		2.16
			BC045083	EEF1A1	4.06	CB562066		2.16
			AB117614	FGFRL1	4.06	BC082709	CCR2	2.16
			BC070543	AQP3	4.06	BC086466		2.16
			BC068831	PLCG1	4.05	BJ050558		2.16
			AY318878	HUNK	4.05	BX844434		2.16
			BC074275	NXN	4.03	CV077651		2.16
			BC073353		4.03	AB070723		2.15
			BX854059		4.01	BC084887		2.15
			BJ085893		3.99	BP683892		2.15
			BJ631095		3.99	BX851075	PRSS27	2.15
			BG730630		3.99	BC044705	MEOX2	2.14
			BC046852	BIN1	3.99	D49837	GUCY2C	2.14

			BC045027	TES	3.99	BF048582		2.14
			BC075161	ETS1	3.98	AF109923	GLI2	2.14
			BC059981	PLD3	3.98	CD330023		2.13
			BP708254		3.98	BC090253	PCCA	2.13
			BC077701	FCHO1	3.98	BC073642	AASS	2.13
			AF440822	PTPNS1	3.97	BC079817	RCSD1	2.13
			U89265	KCND3	3.96	CD255791		2.13
			BQ397842		3.96	CF284526		2.13
			BX852154		3.96	BX846575		2.12
			BC056035	SLA2	3.95	BC055991	EMID1	2.12
			AJ345114	GRM1	3.94	CB944409		2.12
			BX851652	TRIM16	3.93	BC068952	EML2	2.12
			BJ636788		3.93	BC084606	RAB37	2.12
			D83649	SOX7	3.91	BJ612247		2.12
			CF271792	C14orf49	3.91	BC060396	ELMO2	2.12
			BX845355	DOCK10	3.91	CK798371		2.12
			BC077767	CHRD	3.90	BG578993		2.12
			CA973076	PAPSS1	3.90	BC073349	ADRB2	2.11
			X52692	ETS1	3.90	CA973023	MAPK7	2.11
			BC073590	KIAA1166	3.90	AW199362	CLK2	2.11
			BC041733	SH2D3C	3.89	BC070838		2.11
			AF044080	NCOA3	3.89	BG161509		2.11
			AY762376	SMARCA2	3.89	BC073583	EAF1	2.11
			BC068835	SLC6A14	3.88	BC092341		2.11
			BC082414		3.87	BP732418		2.11
			BC057732	SEPT9	3.87	BC061652	HSD11B1	2.11
			BC047258	GCAT	3.86	Y13069	DPYSL3	2.11
			BJ061386		3.86	BC046701	FAM62B	2.11
			BC072365		3.85	BC090224	SULT1E1	2.10
			BC073348		3.84	AY352638	MTUS1	2.10
			BC046257	CNN2	3.84	CD361037		2.10
			CF271689		3.84	BC074173	SLC16A3	2.10
			BC080025	ADSSL1	3.83	BC084120	HPD	2.09
			CD253823		3.83	BC077911		2.09
			CV079892	EMR3	3.83	AY340103	PER2	2.09
			BC088805		3.83	BG409953		2.09
			BC084787		3.82	BP703429		2.09
			BX849960		3.82	U29448	NODAL	2.08
			BC059337	FAM49A	3.82	BC048021	GCNT1	2.08
			BC060753	COL3A1	3.82	BJ070981		2.08
			BC074342		3.82	CD303416		2.08
			BC045118	PCDHGA12	3.81	BX850733		2.08
			BC087334		3.81	BC074410		2.08
			BX850169	A4GNT	3.80	BC045258	CTNNB1	2.08
			BC072875	HOOK3	3.80	CF270416		2.07
			BC046703	EMP3	3.80	BC081179		2.07
			BC073349	ADRB2	3.80	BC043876	PGM1	2.07
			BC041265	TUBB6	3.79	BX851139		2.07
			BF428025		3.78	BC043786	SLC37A1	2.07
			CF521858		3.78	AJ555187		2.07
			BC077824	CALU	3.77	BC073525		2.07
			BC092343		3.77	BC084923	GNAI2	2.07
			BC077878		3.77	CB941351	PALM2	2.06
			U54497	ITGA4	3.76	BC042309	H3F3A	2.06
			U04707	CDH1	3.76	BC056055	LCP1	2.06

			BC084941	GLIS2	3.76	BC081037	TGIF2	2.06
			CF285070	VMO1	3.75	BC084227	PHACTR2	2.06
			BC081243		3.75	BC081167	TIMP2	2.06
			BC043747	MYCN	3.75	AF310007	SALL1	2.06
			BC068733	HS2ST1	3.74	BC059300	RHOG	2.06
			BC085029		3.74	M29857	IGF1	2.06
			BC089283	SOX3	3.73	BC057739	RUNX1	2.06
			AW646020	LRCH2	3.73	BQ737487		2.06
			BG730432		3.73	BC087549		2.05
			BC046671	SPIB	3.73	BC063735	GNAS	2.05
			BC077941		3.72	BC073324	RAPGEF1	2.05
			BC072049	C9orf28	3.72	BF615296		2.05
			BC070978	BCKDK	3.71	BC056857	SNAI2	2.05
			BP716170		3.70	BC073402		2.05
			BC080388	EMILIN2	3.70	BC074390	CADPS	2.05
			BC084270		3.70	BC068891	ACOX2	2.05
			CK799264	CST3	3.69	BX848880	ARSB	2.05
			BC059296	SOX4	3.69	BC073433	MAL2	2.05
			AB093560	IQGAP1	3.68	BF048004		2.04
			BC070645	FAM109A	3.68	BC080111		2.04
			BC072100		3.68	CD256700		2.03
			AF244359	RGS19	3.67	BC081172	GDI1	2.03
			BC059983	SIAE	3.67	AW638861		2.03
			BC046667	CTSB	3.67	BQ731232		2.03
			BC074140	NCF2	3.66	BC080054	LDHB	2.03
			BC077498		3.66	CF519906		2.03
			AW200266	SLAMF8	3.65	BC079763	UAP1L1	2.03
			BC073237	PARD6G	3.65	BC082687		2.03
			AW200140	BTN3A1	3.65	BC072912	SSTR2	2.03
			BJ630546		3.65	BC078019	PRKCD	2.03
			BC044027	SFXN1	3.64	BC091628		2.02
			CK805510	KCNE1L	3.64	AJ344435	FOXD2	2.02
			BJ046308		3.62	BC070645	FAM109A	2.02
			BX848320		3.61	BC070655	HIGD1A	2.02
			BC073607	CYP2D6	3.61	AY260733	PPP1R1A	2.02
			BC099326	HES5	3.61	BC073576	RSU1	2.02
			BP702886		3.61	AW200607	SEMA4A	2.02
			BC075132	FKBP14	3.60	BC049177	ANKRD25	2.01
			AF043643	PCDH7	3.60	J05180	TGFB1	2.01
			BC073392		3.60	BC059995	CPN1	2.01
			BC097728	AGTRAP	3.60	BC075244		2.01
			BX850117		3.59	BC080402	ZNRF1	2.01
			BC080111		3.59	BC088805		2.01
			BX847771		3.59	BC088939	TPM3	2.01
			BC068723	TRIM36	3.58	BC084391		2.01
			BX846232		3.58	BC074116		2.01
			BC049004	TUBB	3.58	BC074247		2.00
			CK797847		3.58	BC084263	GNB1	2.00
			M76710	NCAM1	3.58	BC045050	MYO5A	2.00
			BC084886	RGS4	3.57	BC078136	MMP17	1.99
			CF547880		3.57	BG438924	PTK2	1.99
			BC083045		3.57	BF024854		1.99
			AF055980	IGF1R	3.57	AF061727	SERPIND1	1.99
			BI068185		3.57	BF025586		1.99
			BC086466		3.56	CF286639		1.99

			BC090161		3.56	BC059337	FAM49A	1.99
			AB001073	ACVR1	3.56	BC068782		1.99
			BC090231	GPC4	3.56	BI312922		1.98
			BC090055		3.55	BX846071	CA12	1.98
			U77640	LFNG	3.55	BQ733226		1.98
			BC084782	PLXNA1	3.54	BE679575		1.98
			BX851861	SLC38A6	3.54	BC079819		1.98
			BP695201		3.52	CD361010		1.98
			BC087402		3.52	BC078517	RGS1	1.98
			BC073577		3.52	BJ623141		1.98
			BC066119	C7orf19	3.52	BC071000	AGPAT4	1.98
			BC082447	SRGAP2	3.52	BC073695	KLF11	1.98
			BC077489	YPEL1	3.51	BC088967	B3GNT5	1.98
			CF283947		3.51	BC068831	PLCG1	1.97
			BC080021	ASAH1	3.51	AF032382	ADAM9	1.97
			CB941493	MCAM	3.51	X66959	TCF3	1.97
			BC044039	ID3	3.51	BX842732		1.97
			BC072910	KAZALD1	3.50	BC046745	PADI2	1.97
			BC088952	TRAM2	3.50	BC083034		1.96
			BC092316		3.50	BC083036	MOSPD2	1.96
			BG038211	VAT1	3.50	M22834	H1F0	1.96
			CB943716		3.50	BC077281	PRKACA	1.96
			BC084788	TMBIM1	3.49	BC085220		1.96
			BC073390	CLDN5	3.48	BC077639		1.96
			BC081076	SLC2A6	3.48	BF612190		1.96
			BC078612	NME3	3.48	BJ036670		1.96
			U35408	KLF9	3.48	U09135	PTPRA	1.96
			BX853754	CKAP4	3.48	BC082642	RAB14	1.96
			CK797913		3.47	BF231908		1.95
			BC073205		3.47	BC071132	CD9	1.95
			BJ630946	ME2	3.47	BC092033	GPA33	1.95
			BP722273		3.47	AF549892		1.95
			BC070723	TSPAN17	3.47	BC044968	EMP2	1.95
			BC054166	ID3	3.46	BC076646	PHYH	1.95
			BM928981	PVRL3	3.46	BG019755	PYCR2	1.95
			BC057735	LOH11CR2A	3.45	BX851706		1.95
			BC087391	SCPEP1	3.45	BP687254		1.95
			BC076721	FGF13	3.45	BC077629		1.95
			BC077221	ACTC	3.45	BJ083221		1.95
			CB944946		3.45	BC081239	CSRP1	1.95
			CF287507		3.44	AJ345114	GRM1	1.94
			BJ042570	RNF24	3.44	BG018753		1.94
			BC076776	PLEKHA1	3.43	BC084778		1.94
			BC074204	CYP2A13	3.43	BX847353	S100A14	1.94
			BC077559	PDGFB	3.43	BC073249		1.94
			BC086305		3.43	CB942633		1.94
			AW643311		3.42	BG657077	GPR17	1.93
			BJ047296	LRRC54	3.42	BC088904		1.93
			BC060396	ELMO2	3.42	BX851993		1.93
			BG022428		3.41	BG234189	ARHGAP27	1.93
			BC089181	COL6A2	3.41	BC077938	CBFB	1.93
			BC079918	FAM107B	3.41	BG022174	PPP1R9B	1.92
			BC044016	TGIF	3.41	AW200212		1.92
			BC055991	EMID1	3.40	BC082694		1.92
			BC054306	MOSPD1	3.40	BC076776	PLEKHA1	1.92

			BC075237	C20orf100	3.39	BJ063258		1.92
			BC048222	THBS3	3.39	BC041751	C15orf44	1.92
			AF513854	DIRAS1	3.36	BC079989		1.92
			L38675	NKX2-3	3.35	BC073412	GIT2	1.92
			BC085220		3.35	BC073422	ANXA6	1.91
			BC061657	TNFAIP8L3	3.34	BC057724	EFNB2	1.91
			BC082928		3.34	BC077355	SNN	1.91
			BC057717	SEMA3F	3.34	U63817	TRIM14	1.91
			BC072056	APEX1	3.34	BC088909	SH3GL2	1.91
			BC073296		3.33	BC073092		1.91
			BC057719	FKBP9	3.33	BC074121	GGCX	1.91
			BC045092	SSBP3	3.33	BC081170		1.91
			BX844253		3.32	CK798638		1.91
			BC084281	SNAG1	3.32	BF047391		1.90
			BC082704		3.32	CK804949		1.90
			CF546720		3.31	BC081240		1.90
			BC077302		3.30	BC068927		1.90
			BC074481		3.30	CF283551		1.90
			AY714077		3.29	BG017658	PSD4	1.90
			BC073266		3.29	BC084662		1.90
			BC077854		3.28	BC077701	FCHO1	1.90
			BM191696	ICAM5	3.28	BQ729848	UCHL1	1.90
			BC090217	C1orf102	3.28	AB111447		1.90
			BC042927	KRT12	3.28	U42461	GLI3	1.90
			BP708255		3.28	BC059320	HHEX	1.89
			BC074200	HAO2	3.28	BG513502	ME2	1.89
			BC073412	GIT2	3.27	BC044080	ALDH9A1	1.89
			BC073647	SERPINF1	3.27	BC080118	ADIPOR2	1.89
			BC054158	STMN4	3.27	CA973085		1.89
			BC089182		3.27	BC077245	RAB27B	1.89
			BC044123	GNAI3	3.27	BG729801		1.89
			CX134775		3.27	BC074135		1.89
			BM928954	SLAMF8	3.27	AW766889		1.89
			BC056051	ELK3	3.26	BC087382		1.89
			BC073491		3.25	BC084082	CTBP1	1.88
			BC074383	CCDC28B	3.25	M33874	NOTCH1	1.88
			AW199542	ARL3	3.25	BJ082123		1.88
			BQ733202		3.25	AY686699	EVL	1.88
			BC072272	CD81	3.25	BF072051		1.88
			BC045017	MLSTD2	3.25	BC076719		1.88
			BQ735962	COL8A1	3.25	BP741772		1.88
			BC081144		3.25	BC099017		1.88
			U68059	FRZB	3.25	BQ388127		1.88
			BC070758	RAB11FIP5	3.24	BJ056228		1.87
			BC082695		3.24	BC068723	TRIM36	1.87
			BX846631		3.24	BC044269	CALCRL	1.87
			BC088671	WDR71	3.24	BX853259		1.87
			M22834	H1F0	3.24	BC055972	TFPI2	1.87
			BC084395	NINJ2	3.23	BC056063	FNBP4	1.87
			BG022174	PPP1R9B	3.23	BC071040		1.87
			BC043735	ELAVL4	3.23	BX847954		1.86
			BC078120	ALDH3B1	3.23	BM191386		1.86
			BC082531		3.23	BC077286		1.86
			X12499	HOXC6	3.23	BX851424		1.86
			AB072005	SYNGR1	3.22	BX851355		1.86

			BC081255	SMARCD2	3.22	BC074253	STAU1	1.86
			CK807007		3.22	BC084341	FDXR	1.86
			BX849304	ELA1	3.21	BX848168		1.86
			BC068952	EML2	3.21	BP691429		1.86
			BC056085	RGS19	3.21	BQ731271		1.85
			BJ084787		3.21	BQ723797	B4GALT1	1.85
			BC059984	IRF1	3.20	BC074368	MKRN1	1.85
			BC082703		3.20	BC084814	C9orf58	1.85
			BC084736	SULT1C1	3.20	BC073716		1.85
			BX851075	PRSS27	3.20	AF411389	BIVM	1.85
			L29495	PIM3	3.20	BC085061		1.85
			BG018205		3.20	BJ616349		1.85
			BX843409		3.20	BC054161	VIM	1.84
			BC097748	SPIN	3.19	BX844523	ZP3	1.84
			CA983391	SEPT9	3.19	BE192039		1.84
			BJ034583		3.18	AF187864	MARCKSL1	1.84
			BE576624		3.18	BC056012	FAM3D	1.84
			BX848180	TCTEX1D1	3.17	BG360221		1.84
			BC072937		3.17	BJ619086		1.84
			CD253944		3.17	BX854457		1.84
			BC073243		3.16	U60093	RBPSUH	1.84
			BC063274	GPR124	3.16	AY262358	CD99	1.84
			AF317656	AKT1	3.15	BC076834	COQ10B	1.84
			AY046070	SLIT2	3.15	BC073301	TSPAN18	1.83
			BG264321		3.15	AW634233	ATF1	1.83
			BC047249	GNG7	3.14	BP687284		1.83
			CF286639		3.14	BC074206		1.83
			CF522601		3.14	BX849222		1.83
			BX848873	RGS2	3.14	CF548399		1.83
			U60093	RBPSUH	3.13	CK800469		1.83
			BC077577	AQP3	3.13	AF318178	GCGR	1.83
			BC046657	PTAFR	3.13	BC084788	TMBIM1	1.83
			BC084120	HPD	3.13	BC084404	CSRP3	1.83
			AF197944	PTPRD	3.13	BC056838	CYR61	1.83
			BG038061		3.12	BC084294		1.83
			BC084362		3.11	BC097855	HNRPD	1.83
			BC084801		3.11	AF207901	CGN	1.82
			BC070837	PLCG1	3.11	BC059963	BACE2	1.82
			BI315029		3.11	BC045232	WDR23	1.82
			CA982820		3.11	BC045226	CRLF3	1.82
			BC047255	COL6A1	3.11	BG022166		1.82
			BC057746	SMAD1	3.11	BC080409	RNF13	1.82
			BC057722	KIAA1914	3.10	BJ069899		1.82
			BC079826	MR1	3.10	BC054281	HNMT	1.82
			U77639	SMAD1	3.10	BJ090358		1.81
			BC044330	SLC16A3	3.09	BC084860	ME3	1.81
			BP704574	CDC42SE2	3.09	BC087297		1.81
			BC061947	KIT	3.09	BC070794		1.81
			BC056020	PRPH	3.08	BC076751	B3GNT1	1.81
			BC083015	SIGLEC12	3.08	BC043749	FYN	1.81
			BC074306	HINT3	3.08	BC070558	ITGB1	1.81
			AF346838	TRPS1	3.08	BC073622		1.81
			BC072345	FAM113A	3.08	CF285662		1.81
			BE679528		3.08	BC070538	RAP2B	1.81
			BC084957		3.08	BC077485	ATP1B3	1.81

			BC077503	SNAG1	3.07	BC070750	DDEF2	1.80
			BC088909	SH3GL2	3.07	BC074476	RABL2B	1.80
			BX850179		3.07	BC077187	IRF2	1.80
			BC083034		3.07	BE131768		1.80
			BC079733		3.06	BQ736298		1.80
			BC072163	PYGB	3.06	AB008773	PROX1	1.80
			U18775	TLE1	3.06	BC088670	LOXL1	1.80
			BJ060417		3.06	BX842824	NAALAD2	1.80
			X66959	TCF3	3.06	CF285602		1.80
			BJ068535		3.05	BC082941	DBN1	1.80
			BX851828		3.05	BE669296		1.80
			BC084216	ORMDL1	3.05	CK798105		1.80
			BC043741	FKBP9	3.05	BC042351	GRASP	1.79
			BJ063903	FAM107B	3.04	BC078557		1.79
			BC042267	CEPT1	3.04	BX852372	LLGL1	1.79
			BC079718	ARRDC2	3.04	BC077335		1.79
			BJ627001	PRX	3.04	CD330078	NUBPL	1.79
			BQ731009		3.04	AF302423	DDX21	1.79
			BC068752	SOCS3	3.04	BC070735		1.79
			U66288	WNT2B	3.04	BC088937		1.79
			CF548888		3.04	BG553375		1.79
			AW634666		3.04	BX843356		1.79
			BC054285	CDK2AP1	3.04	BX844068	B3GNT7	1.79
			CF547820		3.03	BX849457		1.79
			BC072123	SOX17	3.03	BC045211	C1orf77	1.79
			BC072951		3.03	BC072179	PDLIM7	1.79
			BX854030		3.03	BC084969	SSPN	1.79
			D87209	SOX11	3.02	DQ096892		1.79
			BP709295		3.02	BC072800	CD9	1.78
			BC081135	CCNI	3.02	BC077289		1.78
			CF270696		3.02	BC081168	DDAH1	1.78
			BG233646	S100A4	3.02	BC084930	CAMK1	1.78
			CD325861		3.02	BI068185		1.78
			AY686699	EVL	3.01	BP705466		1.78
			BC076801	FAM26B	3.01	BP688919		1.78
			BP687487		3.01	AW148133	C22orf3	1.78
			BC068797	HEBP1	3.01	BC068768	EPS8	1.78
			BC074379	RASGEF1A	3.01	BC079972	TCF7L2	1.77
			BF610645		3.01	BF071784	FAM58A	1.77
			CD329717		3.01	BC073478	FAM102A	1.77
			BC078579	PSKH1	3.01	BF025071	ALOX15B	1.77
			BC089259		3.01	BC073240	MYO1D	1.77
			BX851536		3.01	AF002983	CDH11	1.77
			D49373	CFB	3.00	AF319538	EXT1	1.77
			BQ732897		3.00	BC088746		1.77
			BC072174		3.00	BC092329		1.77
			CK797035		3.00	BC087402		1.77
			AF302766	SMO	3.00	CF287379		1.76
			CD099389		3.00	BC047245	PYGB	1.76
			AW199681	ME2	2.99	BC043890	LECT1	1.76
			BC077880	PMP22	2.99	CD100547		1.76
			AF033110	FZD5	2.98	BX854219	DNAJB1	1.76
			BJ088669	RAB20	2.98	BC074268	C18orf43	1.76
			BC077193	TXNIP	2.98	BJ080002		1.76
			BC077468	CAV2	2.98	U75996	EOMES	1.76

			BC072271	GUCY1B3	2.98	BC056102	C1orf21	1.75
			BJ081018		2.98	BJ066554		1.75
			BC079762		2.98	AW645391		1.75
			BC072082	MTHFD2	2.97	BX852552		1.75
			BC089247	WDR69	2.97	AF440822	PTPNS1	1.75
			CK798484		2.97	CF342388		1.75
			BJ050974		2.96	CK805226		1.75
			BC072348	LOH11CR2A	2.96	CF287653		1.75
			CF287096		2.96	BG016865		1.75
			AB111446		2.96	X66979	FLI1	1.75
			CF521264	METTL1	2.96	BC074283		1.74
			BF615469	SH3BP4	2.96	BC070788	LLGL1	1.74
			BC084795	SH3GL3	2.95	BC044084	IGSF4B	1.74
			BC073324	RAPGEF1	2.95	BX843353	RALGDS	1.74
			AF154112	SIN3A	2.95	BC059313	CD2BP2	1.74
			AW200275		2.95	AW643311		1.74
			BC058326	SDS	2.94	BC073320		1.74
			BC060494	SP4	2.94	BJ041471		1.74
			BC077967	FNDC3A	2.93	BP687360		1.74
			BC079989		2.93	CK798161	SOD1	1.73
			BP704537		2.93	BC077559	PDGFB	1.73
			AB042255	CRY1	2.93	BP702839		1.73
			BC043760	ALPL	2.93	CB942111		1.73
			AB052692	SOX17	2.93	AJ009292	BCL10	1.73
			CD302358	COL6A3	2.93	BJ619579		1.73
			BC057743	CUGBP1	2.93	CD325861		1.73
			BC082446		2.92	BC060442	DIDO1	1.72
			AJ635202	GPR68	2.92	BC079707		1.72
			AY762636		2.91	CK798484		1.72
			BC073559	XYLT1	2.91	CF270683		1.72
			BC070743	REV1L	2.90	BC047254	CUEDC1	1.72
			BC068750	SH3BP4	2.89	BG814570	APBA1	1.72
			BQ734359		2.89	CF290385		1.72
			BC080085	EDIL3	2.89	BC072191	ZNF217	1.72
			BG893369		2.89	AW199462	NAALAD2	1.71
			CF283551		2.89	BC072204	LIMS1	1.71
			BX847357		2.89	BC070603	RNF111	1.71
			BC071011	KBTBD2	2.89	CD253051	C1orf149	1.71
			BC059297	PPT2	2.88	BF610645		1.70
			BC084662		2.88	AF364821	MYL4	1.70
			AF157558	NR2F1	2.88	BC061685	CTSD	1.70
			BC077597	SH3GLB2	2.88	BC075249	NDRG4	1.70
			CK798340		2.88	BJ036402		1.70
			BG234127	LTBP3	2.88	BX843871	STYK1	1.70
			BX848330		2.88	BC070978	BCKDK	1.70
			BC043887	PRPSAP1	2.87	CB945344		1.68
			AW200150		2.87	AW199542	ARL3	1.68
			BF612190		2.87	CX135390		1.67
			BX850931		2.87	BC070823	SH3MD2	1.67
			CD324647		2.87	BC077778	GNG7	1.67
			BC072752	FREQ	2.87	CD327732		1.63
			CD327121		2.87			
			AW645526	MDM1	2.86			
			CF270683		2.86			
			CB562980	TTC8	2.85			

			BC078012	RASGRP2	2.85			
			BX853841		2.85			
			BC082950	PPGB	2.85			
			BC042348	FAM49B	2.85			
			BX844450	C16orf48	2.84			
			CD100497		2.84			
			BC073726	KIFAP3	2.83			
			BC077601	B4GALT3	2.83			
			BC041731	HOXA3	2.83			
			BF612406		2.83			
			BQ735488	EP400	2.83			
			BJ640299	RHOB	2.83			
			BX844585		2.83			
			BC057696	CTDSP2	2.82			
			BG162643		2.82			
			BX850461	ATHL1	2.82			
			AF212298	SEPT2	2.82			
			BX852352		2.82			
			CD303400		2.82			
			BP688782		2.82			
			BC042275	EGFL6	2.82			
			BC084339	WDR37	2.81			
			BC046719	PFN2	2.81			
			BC054225	HSPB8	2.81			
			BC088947	ACVR1	2.80			
			BG264233		2.80			
			CV076436	ART5	2.80			
			BC082952	PYGL	2.80			
			CK799983		2.80			
			AW199861		2.80			
			BC084896		2.80			
			BC089265	C1orf52	2.80			
			BG162303	TNS3	2.80			
			CA982783	PEO1	2.80			
			BC048219	CPT1A	2.79			
			BC077620	WDR37	2.79			
			BX846329	AGPAT1	2.79			
			BC079763	UAP1L1	2.79			
			M81683	ETS2	2.79			
			BC083033		2.79			
			BC056842	LGMN	2.79			
			BC080090	OGDHL	2.79			
			BJ062290		2.79			
			CK804709		2.79			
			CK799129		2.79			
			BJ066554		2.78			
			BC056079	ARHGDIB	2.78			
			AB049354	FSTL1	2.78			
			BC080036		2.78			
			BG553396		2.77			
			AW646642		2.77			
			BE575410		2.77			
			BF612440		2.77			
			BX853578	PIN4	2.77			
			CB563548	TMSL3	2.77			

			BF232099	SEMA3A	2.77			
			BP741831		2.77			
			BP677676		2.76			
			U81958	BMPR2	2.76			
			AY278679	FUT3	2.76			
			BM191279		2.76			
			CF290385		2.76			
			BC061674	GPR146	2.75			
			AF081803	HCK	2.75			
			BC044117	PELI1	2.75			
			BJ035117		2.75			
			AY363162	TECTA	2.74			
			BC073347		2.74			
			BC072769	RGS4	2.74			
			BF615400		2.73			
			BC077919		2.73			
			BC043783	YES1	2.73			
			CK805269	FTH1	2.73			
			BX848893	PGLYRP1	2.73			
			AF482757	GTF2IRD1	2.73			
			AF053935	NOS1	2.72			
			BC081134	SAV1	2.72			
			BC090159		2.72			
			CK797591		2.72			
			AW636919		2.72			
			BC072148		2.72			
			BC072787	CLIC4	2.72			
			U68387	MEIS1	2.72			
			BC075234		2.71			
			AF508961	LRP6	2.71			
			BC068931	PRKAR2B	2.71			
			BG018753		2.71			
			CF521551		2.71			
			BC082936		2.70			
			BC087383		2.70			
			AF224746	BICC1	2.70			
			BC084811	DUSP23	2.70			
			AF172399	NGFR	2.70			
			BC084360	CD53	2.70			
			BC077539	PTP4A1	2.70			
			BQ732917		2.69			
			BG161397		2.69			
			BX846572		2.69			
			BC089285	C6orf134	2.69			
			BC089169	AK7	2.69			
			BX853276		2.68			
			AW640053	PIN4	2.68			
			CF271887		2.68			
			BC080402	ZNRF1	2.68			
			BE679557		2.67			
			BC046837	P2RY4	2.67			
			BC077893		2.67			
			AY216793	ENC1	2.67			
			AW199587		2.67			
			BC046679	SFRS1	2.67			

			BC073267	ENTPD1	2.67			
			BC071089	NAGA	2.67			
			BJ073273		2.67			
			DR719140		2.67			
			BC078527	IGSF4C	2.66			
			BC056109	CTSC	2.66			
			BC077960	PRTFDC1	2.66			
			BP685880		2.66			
			CB944643		2.66			
			BG410073	WDR41	2.66			
			BC081284		2.65			
			BC097583	UAP1L1	2.65			
			BC072967		2.65			
			CF289954	MXRA7	2.65			
			BF231930		2.65			
			BC081047	SEPT11	2.64			
			BC084233	TMEM110	2.64			
			AW639557		2.64			
			BC068743	C22orf5	2.64			
			BC087423		2.64			
			CF286923		2.64			
			U39929	CBX4	2.64			
			U41761	SDC3	2.64			
			AB034701	COL1A1	2.64			
			BC074252		2.64			
			AJ311602	KIF3A	2.63			
			BC070590		2.63			
			BC081193		2.63			
			BJ072373	SARDH	2.63			
			BG162675		2.63			
			AF427862	PECAM1	2.63			
			BX847871		2.63			
			BC080168	NEGR1	2.63			
			BJ623174		2.63			
			AW200487		2.62			
			BC074374	PAICS	2.62			
			D87752	AMBP	2.62			
			BC077253	GPM6B	2.62			
			BC046678	CLU	2.62			
			BC080097		2.62			
			BE026509		2.62			
			BC043808	CNN3	2.62			
			CF342082		2.62			
			BC045043	ARPC1B	2.61			
			BP699378		2.61			
			BC082817	MYADM	2.61			
			CK798371		2.61			
			BC070538	RAP2B	2.61			
			BC076779	MAPKBP1	2.61			
			BX851320		2.61			
			BC072779	EHD2	2.60			
			BF610679		2.60			
			AF032382	ADAM9	2.59			
			CF285540		2.59			
			BG345217		2.59			

			BC041248	FKBP1A	2.59				
			AW639424		2.59				
			CK797904	C3orf38	2.59				
			BX850954		2.59				
			BC077450	ZNF161	2.59				
			BG513848		2.59				
			BJ077113	ZNF420	2.58				
			CK798368		2.58				
			AW200477		2.58				
			BG163156	KCNE3	2.58				
			BF072310		2.57				
			M94969	ABI2	2.57				
			BC086473		2.57				
			X57675	CDH2	2.57				
			BJ073152		2.57				
			AW638291		2.56				
			CF286792		2.56				
			BX845870		2.56				
			CK798694		2.56				
			BC068780		2.56				
			BC068659	REEP5	2.56				
			BC075244		2.56				
			BP686017		2.56				
			BC075223	RGS10	2.55				
			AW200562	LOC147991	2.55				
			BC057731	SEPHS1	2.55				
			BC041721	CAMK1	2.55				
			AJ549811	HTR2B	2.55				
			BC082678	C2orf32	2.54				
			BX845823		2.54				
			X56870	MYC	2.54				
			BJ078526		2.54				
			BC060350	SOX17	2.53				
			BC082477	PTK9L	2.53				
			BC081003	SDCBP	2.53				
			BC079984		2.53				
			BC099353	SLC36A4	2.53				
			BJ638827	RAB20	2.53				
			AF351126	EZH2	2.53				
			BF048004		2.52				
			BJ030923	C1orf78	2.52				
			BC076802		2.52				
			BC081051	PIP5K2B	2.52				
			BC043893	PLOD3	2.52				
			BC080112		2.51				
			BC077738	PURA	2.51				
			BX850702	C10orf38	2.51				
			BC081227	TMEM111	2.51				
			BC076836		2.51				
			BX849724	ZNF436	2.51				
			BJ612457	MLL2	2.51				
			AB015205	CNTN1	2.50				
			BC086461	TNIP1	2.50				
			BC084099	HISPPD1	2.50				
			BC055972	TFPI2	2.50				

			BC070842		2.50			
			BC046572	ZNF238	2.50			
			BF427048		2.50			
			BC084746		2.50			
			BJ057306	C14orf1	2.49			
			CF548877		2.49			
			BC044008	MST1	2.49			
			BC080043	MAP4K5	2.49			
			BC073413	C2orf4	2.49			
			BC084111	KIAA1826	2.49			
			BE576135	COMP	2.49			
			BG413880		2.49			
			BJ619086		2.49			
			L25856	HOXA1	2.48			
			BC041727	PMP22	2.48			
			BC068947		2.48			
			CX134846		2.48			
			BC075248	STMN2	2.48			
			BC073429	BPGM	2.48			
			BC074340		2.48			
			BE506346		2.48			
			BQ730753	KIF26A	2.48			
			BC081172	GDI1	2.48			
			BC061934	STOX2	2.47			
			BF231562		2.47			
			BX850302		2.47			
			BC088804	C8orf42	2.47			
			BJ058524		2.47			
			BC077476	INA	2.47			
			BC088967	B3GNT5	2.47			
			BC082475	SNAP25	2.47			
			BC077275	RGS5	2.47			
			BC077648	CLEC10A	2.46			
			BC088790	GPX7	2.46			
			BJ064568		2.46			
			BC060497	HSD17B6	2.46			
			BP715062		2.46			
			BG021249		2.45			
			BJ085145	ORMDL2	2.45			
			BC079978	FUT8	2.45			
			BC074249	TSPAN5	2.45			
			BC087460	WNT4	2.44			
			BC077169	TIA1	2.44			
			BF614679		2.44			
			BC074390	CADPS	2.44			
			BC077864	SERPINE2	2.44			
			BG264344	GLRX	2.44			
			CA973750		2.43			
			BQ731232		2.43			
			BC073471		2.43			
			BP707389		2.43			
			BC045013	SPARC	2.43			
			BC071068	RAB2B	2.42			
			BC072231	ARL8B	2.42			
			BC042274	GNS	2.42			

			BC077463		2.42			
			BC088945	CYYR1	2.42			
			BC094114		2.42			
			AW645397		2.42			
			BC087400	ANKRD45	2.42			
			BC094085		2.42			
			CD327154		2.41			
			BC070701		2.41			
			BJ037405		2.41			
			BC060476	C9orf7	2.41			
			BC075168		2.41			
			BC075230		2.41			
			AF061727	SERPIND1	2.41			
			BJ055135		2.40			
			AY280863	CTDSPL	2.40			
			BJ063104		2.40			
			BC044956	RNPC1	2.40			
			BQ386276		2.40			
			BQ732561	DGKD	2.40			
			BJ050057	CASR	2.40			
			CD327260		2.40			
			CF342159		2.39			
			BC043746	SLC19A3	2.39			
			BC044671	MAPRE1	2.39			
			BC090253	PCCA	2.39			
			BX843661		2.38			
			BE189593	ASB6	2.38			
			BP685122		2.38			
			BC081176	LHFPL3	2.37			
			BG814570	APBA1	2.37			
			BP689420		2.37			
			BC072259		2.37			
			BC060458	POLR2B	2.37			
			BC068802		2.37			
			CK796526		2.37			
			BG485959		2.36			
			BC088667	M6PR	2.36			
			BC078069		2.36			
			BC078138		2.36			
			BJ052099		2.36			
			D83476	BMP1	2.36			
			M76566	GATA1	2.35			
			BC077740	MBD3	2.35			
			BC082350		2.35			
			BC042290	H3F3A	2.35			
			BX845117	P2RY5	2.35			
			BQ735822		2.35			
			BC070666	EPB41L4A	2.35			
			BC097784	SNX30	2.35			
			AB025112	NPR2	2.35			
			AY008299	ILF3	2.35			
			BX843406		2.35			
			AF107662	ETV1	2.34			
			CF286684		2.34			
			BC068770	STAG1	2.34			

			CF548267		2.34			
			AB109555	AHR	2.34			
			BQ730962		2.34			
			AJ009284	HES6	2.34			
			BC081281		2.34			
			BX848766	TSC1	2.34			
			BC071042	CYP2C18	2.34			
			BC070710	MAX	2.34			
			BP698662	HBXAP	2.34			
			BX848455		2.34			
			CK805128	FKBP1A	2.34			
			BC044686	PCMTD2	2.33			
			BC082440	LRRC14	2.33			
			CB944409		2.33			
			CD301757		2.33			
			U14169	CPEB1	2.33			
			AW636221		2.33			
			BX852823		2.33			
			CV072922		2.33			
			BC044697	TSPAN7	2.32			
			BC070788	LLGL1	2.32			
			BC071000	AGPAT4	2.32			
			BC087415		2.32			
			D14400	ITPR1	2.32			
			BC077324	PTGES	2.32			
			BC073606		2.32			
			BP699151		2.32			
			CF285539		2.32			
			CF548303		2.32			
			AF411389	BIVM	2.32			
			BX853414	MYO18A	2.32			
			BC089137		2.31			
			BC077852		2.31			
			BP709723		2.31			
			BQ733975		2.31			
			BC070641		2.31			
			BC081121	INPP5A	2.31			
			BP698716		2.31			
			BC074369	GGT1	2.31			
			AW644128		2.30			
			BC068782		2.30			
			CD327200		2.30			
			AF321229	INVS	2.30			
			BC082859		2.30			
			BC043751	RPE65	2.30			
			BJ034898		2.30			
			BP705893		2.30			
			BC045226	CRLF3	2.30			
			BC079971	SLC15A4	2.30			
			BC084615		2.30			
			BX853985		2.30			
			BC044316	EDNRA	2.29			
			BC054951	EHD2	2.29			
			BC082896		2.29			
			BX846116	MUC3B	2.29			

			BC072119	VANGL2	2.29			
			CK804838		2.29			
			BC089173	KLHL17	2.29			
			BC074313	ALCAM	2.29			
			BC063738		2.29			
			BC089272	PTTG1IP	2.29			
			S61773	INHBB	2.29			
			AB096099	EIF4G2	2.29			
			BG159797	GPR23	2.29			
			BC054214	SOCS3	2.28			
			BC056023	FLT1	2.28			
			BC045258	CTNNB1	2.28			
			BG037975		2.28			
			BC080399		2.28			
			CA973023	MAPK7	2.28			
			BC090252	GNAO1	2.28			
			BC072732	CBLB	2.28			
			BC081089	RAB3GAP1	2.28			
			CA983203		2.28			
			BC084083	SHKBP1	2.28			
			CB943529	GIT2	2.27			
			BG161509		2.27			
			CK798446		2.27			
			BC074404	NBL1	2.27			
			BG162094	SSBP3	2.27			
			BX842912		2.27			
			BC084803		2.27			
			CK800686	HELZ	2.27			
			BE679390		2.27			
			BC061941	CCDC63	2.27			
			CK800545		2.27			
			BC084089	NID2	2.27			
			BC056041	PLA2G4A	2.26			
			BF615536		2.26			
			BC074168	NEU1	2.26			
			AY303832	CAP2	2.26			
			BE026027		2.26			
			BC045253		2.26			
			CX134065		2.26			
			BJ078216		2.26			
			CA972148		2.26			
			BU152721		2.26			
			AF059570	SFRP2	2.26			
			AW766900		2.26			
			BF613768		2.26			
			BC082842	PDK1	2.25			
			BG038337		2.25			
			BC086263		2.25			
			BC047965	KARS	2.24			
			BC077400	UBXD4	2.24			
			BC084408		2.24			
			BP693006		2.24			
			BC071048	PBX2	2.24			
			CV080315	PDLIM4	2.24			
			BC077300	PAG1	2.24			

			CK797924	NPC2	2.24			
			BC072971		2.24			
			BP699675		2.24			
			BC072991	SIRT6	2.23			
			BC073263		2.23			
			BE505183		2.23			
			CV076058		2.23			
			BC087464		2.23			
			CD328493		2.23			
			BG160708		2.23			
			AW635234		2.23			
			BC073659		2.23			
			BC077778	GNG7	2.23			
			BX848880	ARSB	2.23			
			BJ046282		2.23			
			BC079775	RAP1GDS1	2.23			
			BJ081049		2.23			
			BX848354	SYN3	2.22			
			BG016741	IMPDH2	2.22			
			BC084227	PHACTR2	2.22			
			AW148044		2.22			
			BC081023	EDG4	2.22			
			AF546707	TUBD1	2.22			
			AY344472	LDLRAP1	2.22			
			BC085039	CRIP1	2.22			
			BG020077		2.22			
			BC077871	GPR137B	2.22			
			BJ030698		2.21			
			BX847776		2.21			
			CK798533	TMEM107	2.21			
			BE576657	RASA2	2.21			
			CA974505	THRAP6	2.21			
			BF611979		2.21			
			BC084885	NIPA2	2.21			
			BC089172	SLC18A1	2.21			
			CD326197	SULT1C1	2.21			
			AF274053	KCNMA1	2.21			
			BG022306		2.21			
			M98807	NOG	2.21			
			BC074356	EDG1	2.20			
			BC074307	PRNP	2.20			
			BJ038635		2.20			
			BC073525		2.20			
			BJ617381		2.20			
			BC060480	GPRC5A	2.20			
			BJ042255		2.20			
			U64442	APC	2.20			
			CK800034	C14orf93	2.20			
			BC082647		2.20			
			CK798214		2.20			
			BC074347	TRPC3	2.20			
			BG234611		2.20			
			BX848095		2.20			
			BC075217	NIPSNAP3A	2.20			
			BC075249	NDRG4	2.20			

			BC070719	VPS24	2.19				
			BE506594	STIM2	2.19				
			BC054258	ATP6V0C	2.19				
			BC060328	CYP2C8	2.18				
			BC077431	TMC7	2.18				
			BC070672	CAV1	2.18				
			BC072824	FAM3C	2.18				
			BC081168	DDAH1	2.18				
			BC084082	CTBP1	2.17				
			BP683892		2.17				
			BC043968	TSPAN31	2.17				
			BE189070		2.17				
			DR718596	PQLC2	2.17				
			BC068642	SMAD6	2.17				
			BC084112		2.17				
			CK797468		2.17				
			BC074123		2.17				
			BC077862	KCTD1	2.17				
			AY484582	RTN1	2.17				
			BC081151	RAB20	2.17				
			BC073422	ANXA6	2.17				
			BJ612287		2.17				
			AW199965		2.17				
			CD252706		2.17				
			M27502	FYN	2.17				
			CF290001		2.16				
			BC060327	EAF2	2.16				
			BC081038	TRIM28	2.16				
			BP703709		2.16				
			BC044315	LYPLA2	2.16				
			BC084388		2.16				
			CB562066		2.16				
			AF302423	DDX21	2.16				
			BC071027		2.16				
			BX849488		2.16				
			BC084409		2.16				
			BJ084667		2.16				
			BC077961	CD63	2.16				
			BX842722	C1orf149	2.15				
			BC084231		2.15				
			BC046658	MIDN	2.15				
			BC078007	HIRA	2.15				
			AW642902	EIF2C4	2.15				
			BC056076	CDC2L6	2.15				
			BC070747	PIAS2	2.15				
			BC072934	PPM1D	2.15				
			BC083029		2.15				
			BC076830		2.15				
			BC080041	GRK6	2.15				
			CA982120	ZNF300	2.15				
			BC068688		2.14				
			BX848111	RSU1	2.14				
			BC074483	CCDC53	2.14				
			BC075209		2.14				
			BC077840	RHOV	2.14				

			BM928912		2.14			
			M24752	HOXA7	2.14			
			AW200038	RFWD3	2.14			
			BC070614	UBE2E3	2.14			
			CD301800		2.14			
			BC060469	ABCC5	2.14			
			BP720937		2.14			
			CD253566		2.14			
			AW644252		2.13			
			BP699483		2.13			
			BX850630	COX15	2.13			
			BC080093		2.13			
			BC084432		2.13			
			BP696539		2.13			
			CD303355		2.13			
			AF388035	CDON	2.13			
			CD253832		2.13			
			BC080006	GALNT11	2.13			
			BG017658	PSD4	2.12			
			AW199724		2.12			
			AY491055	EDIL3	2.12			
			BJ031356	ZNF84	2.11			
			BJ066067		2.11			
			BC073605	SPRY2	2.11			
			BP693239		2.11			
			BC044076		2.11			
			BJ612923	ZNF569	2.11			
			BX844295		2.11			
			CF547458		2.11			
			BC045234	SLC22A15	2.11			
			BC054953	PCOLCE	2.11			
			BC073307	VGLL4	2.11			
			CB756059		2.11			
			BC092034	ASB7	2.11			
			BC043623	RBMS1	2.10			
			BC072216	JUNB	2.10			
			BC082856	AXUD1	2.10			
			BP692816		2.10			
			BC078079	RHBG	2.10			
			BG160506		2.10			
			AW200278	FTH1	2.10			
			BC054279	EEF1A2	2.10			
			BC072744	DUSP22	2.10			
			BQ733226		2.10			
			AW766889		2.10			
			AF432354	P2RY1	2.10			
			BC083017		2.10			
			BC043963	CLK2	2.09			
			BG233711		2.09			
			CA981854		2.09			
			CF285650		2.09			
			AB026192		2.09			
			BC075228		2.09			
			BM180020		2.09			
			AY344472	LDLRAP1	2.09			

			AY057997	GJB3	2.09			
			BC074239		2.08			
			BC093570	PIGW	2.08			
			BC082694		2.08			
			BC082699		2.08			
			BC084363		2.08			
			BP701259		2.08			
			BC046259	HAPLN3	2.07			
			BC081256		2.07			
			BC082682	RABAC1	2.07			
			AW642576		2.07			
			BQ732572	RP1L1	2.07			
			BX843583	CRYAB	2.07			
			BC043792	VIP	2.07			
			BX848258		2.07			
			BC073185		2.07			
			CK800037	CASP6	2.07			
			BC073112	TYK2	2.07			
			CA971610		2.07			
			BG016865		2.07			
			BC084599	BTC	2.07			
			BQ737351		2.06			
			BC070648	SEPT11	2.06			
			BC078071	GUK1	2.06			
			BC046668	SFRS6	2.06			
			BC070803	KLF11	2.06			
			AB001469	LIMK1	2.06			
			BC077570	JAK1	2.06			
			BC071005		2.06			
			BC074142		2.06			
			BJ032419	ARL5B	2.06			
			BI441612		2.05			
			BC084347	PDE4B	2.05			
			CD325358	PGBD5	2.05			
			CK796938	ADNP	2.05			
			BC072894	CDK5	2.05			
			BC073272	H2AFY2	2.05			
			BC076739	KIAA1826	2.05			
			BC087549		2.04			
			BC044977	GALK2	2.04			
			BC081228		2.04			
			BE505678		2.04			
			BC077897	CD81	2.04			
			BC072800	CD9	2.04			
			BE505174		2.04			
			BF615077	FUT8	2.04			
			BP689354	C5	2.03			
			BP721556		2.03			
			BC083012	TSPAN4	2.03			
			BP706611		2.03			
			BC089131	BAMBI	2.03			
			BG162394		2.03			
			CA974443	TH1L	2.03			
			BC077770	HNRPH1	2.03			
			BX852957		2.03			

			AW200608		2.03			
			AY312062	PTPN12	2.03			
			DQ096881		2.03			
			BC043981	ILF2	2.03			
			BC061651	MGRN1	2.02			
			BC068810	LDLRAP1	2.02			
			BC081175		2.02			
			BC079819		2.02			
			BC044107	GCLM	2.02			
			BP693110		2.02			
			M25866	ZNF84	2.02			
			BC060427	FMO2	2.02			
			BC077913	RDH10	2.02			
			BC084246		2.02			
			BC041517	STMN3	2.01			
			BC057724	EFNB2	2.01			
			BC070650	SNX1	2.01			
			BI478163		2.01			
			Z14997		2.01			
			BC087465	SNTA1	2.01			
			BJ072242		2.01			
			AF466017	ARMC1	2.01			
			BC077792	CSF3R	2.00			
			BC071057	ZNF238	2.00			
			BC077587	SAP130	2.00			
			BC073738	C20orf55	2.00			
			BF427443	STK11	2.00			
			BQ730716	NXN	2.00			
			CA983547	MED19	2.00			
			BC072945	GPR157	2.00			
			BX849398		2.00			
			BX853225		2.00			
			CO386691		2.00			
			BC048021	GCNT1	2.00			
			BG017699	AHI1	2.00			
			BQ388100		2.00			
			BQ736650	TXNDC	2.00			
			BX844003		2.00			
			CD253973	TLN2	2.00			
			CV072994		2.00			
			BC073546		2.00			
			BM261189		2.00			
			BC088746		2.00			
			BJ641756		2.00			
			AJ507633	SLC35A3	1.99			
			AW768051		1.99			
			BC059973	C16orf35	1.99			
			BP697143		1.99			
			BC088942		1.99			
			CD303126		1.99			
			CF270864		1.99			
			AF393242	TLL1	1.99			
			BX846234		1.99			
			BX851623		1.99			
			BC074435		1.99			

			AW640794		1.99			
			BC041726	PACSIN3	1.99			
			BE576731		1.99			
			BE680031		1.99			
			BJ612498		1.99			
			AW637943		1.99			
			BC078013	YTHDF3	1.99			
			BC074309	KRT17	1.98			
			CA982525		1.98			
			AW766084		1.98			
			BC077226	YPEL5	1.98			
			BC079814	PIK3C2A	1.98			
			BC087299		1.98			
			BC092118	RNASEH1	1.98			
			BP706688	ANKRD26	1.98			
			CK803867	C1orf121	1.98			
			BC057714	RGS6	1.98			
			BC061649	SNX5	1.98			
			BC076766		1.98			
			AF040993		1.98			
			AW642827		1.98			
			BP694998		1.98			
			CK798479		1.98			
			BC043762	RASSF6	1.98			
			BC044096	SS18	1.98			
			BG160237		1.98			
			CB755900	CAPN7	1.98			
			CK796998	SNAPC5	1.98			
			BX851249		1.97			
			BC044009	HMGB3	1.97			
			BQ734561	KCNE1L	1.97			
			BI314838		1.97			
			BX851854	ING1	1.97			
			BJ057452		1.97			
			BC044267	EGFL7	1.97			
			CK798428		1.97			
			BP694403		1.97			
			BX846134	ATXN3	1.97			
			BC068930	PCGF2	1.97			
			BC084383		1.97			
			BC080018	TRAF4	1.96			
			BG730106		1.96			
			BX847954		1.96			
			BC059324	G6PD	1.96			
			BC075182	RHPN2	1.96			
			BC088715	SAP130	1.96			
			BC099278	OTC	1.96			
			BP684279		1.96			
			BC063732	FAM79B	1.96			
			BG439425		1.96			
			BC044672	PTK9L	1.96			
			CB592993		1.96			
			BC071067	HNRPA0	1.96			
			BC073428	FGFR3	1.96			
			BC071012	PDK1	1.96			

			BC076851		1.96			
			BC082837	SLC44A1	1.95			
			BX854020	PPP1R12A	1.95			
			AB083001	CAMKK2	1.95			
			AF051784	SMC1L1	1.95			
			AF401352	SH2D1A	1.95			
			BC078525	IXL	1.95			
			BC081002		1.95			
			M76565	GATA3	1.95			
			AI031384	ELOVL6	1.95			
			BC074354		1.95			
			BC084833	B4GALT7	1.95			
			CD252842		1.95			
			AF142632	NRG1	1.95			
			BC068778	NEK4	1.95			
			BC079985		1.95			
			BG022440		1.95			
			BX850674	C20orf19	1.95			
			BF614085		1.95			
			BC070607	ZDHHC20	1.94			
			BC085026		1.94			
			BJ091892	C9orf125	1.94			
			BC072204	LIMS1	1.94			
			AY566230	NIN	1.94			
			BC074298		1.94			
			BG019176	KLF11	1.94			
			BX854638		1.94			
			BG730933	NKD1	1.94			
			BQ731442		1.94			
			BC087320	NME4	1.94			
			BX852516	FAM55A	1.94			
			CX132446		1.93			
			BC073677	GTF2F2	1.93			
			BX843873		1.93			
			BC042263	SHOC2	1.93			
			BP741772		1.93			
			AW633477		1.93			
			BC099017		1.93			
			BC041206	CREB1	1.93			
			BP689243		1.93			
			BC092320	HDAC10	1.93			
			BX846603		1.93			
			BC068967	TRIP12	1.93			
			AW199751	ENY2	1.92			
			BC054962	CBX5	1.92			
			BE509350	CD58	1.92			
			CK799314		1.92			
			U66710	MCM7	1.92			
			AW766222	PDCD6	1.92			
			AY885243		1.92			
			BC059290	OPHN1	1.92			
			BF613269		1.92			
			BC079999	SNRPD2	1.92			
			BC084337	RDS	1.92			
			BC059352	PAFAH1B2	1.92			

			BI446334		1.92			
			BC076715	SMARCA5	1.92			
			BF614416	MUM1	1.92			
			AW633166		1.92			
			BJ616349		1.92			
			BC047245	PYGB	1.92			
			CK797487		1.92			
			BC074109	SEC22L2	1.91			
			BJ073801		1.91			
			BX849303		1.91			
			BX851457	KLHL18	1.91			
			AB107103	MAML1	1.91			
			AY504996	C20orf32	1.91			
			BC068758	ZNF507	1.91			
			BG439828		1.91			
			BC056111	CMTM7	1.91			
			BG017789	CD37	1.91			
			AW200112		1.90			
			BC072995		1.90			
			BC089262	SAMD4A	1.90			
			BX854457		1.90			
			BC077174		1.90			
			BX851406	FBXO22	1.90			
			AW640576		1.90			
			BC077373	CEP76	1.90			
			BE506299		1.90			
			BJ042926		1.90			
			CA971430		1.90			
			AW200497		1.90			
			BC073340	PSME1	1.90			
			BC070569	GTF2F1	1.89			
			BC072373		1.89			
			CK799643	ERICH1	1.89			
			BC048020	LMO4	1.89			
			BC088908		1.89			
			BC093534		1.89			
			BP688514		1.89			
			BC043802	THRAP4	1.89			
			BJ092595		1.89			
			BX843561	WDR41	1.89			
			BE575901	ANP32E	1.89			
			AW767274		1.89			
			BC079987	BAMBI	1.89			
			BG022727	CAP1	1.89			
			AY052629	IGFBP5	1.89			
			BF049338		1.88			
			CA982052		1.88			
			BC084940	MLF2	1.88			
			BC072963	TARBP2	1.88			
			BP687360		1.88			
			BP689799		1.88			
			BQ731780		1.88			
			BX850038		1.88			
			CD302825		1.88			
			BC072191	ZNF217	1.88			

			BC072364	AMPH	1.88			
			BC077298	HOXC10	1.88			
			CK797205		1.88			
			BF231668	RAB34	1.88			
			CK800055		1.88			
			BE507181		1.88			
			BJ638911		1.88			
			BX848422		1.88			
			AW200252		1.88			
			BC081114	P4HA2	1.87			
			BF427807		1.87			
			BC056112	CHN1	1.87			
			BP701788		1.87			
			BC068924		1.87			
			BC084428		1.87			
			CK796688		1.87			
			BC092120	ZNF585A	1.87			
			CO385591		1.87			
			BE669182		1.87			
			AF549908	HMGB2	1.87			
			BP687629		1.87			
			BJ088800		1.87			
			BE507732		1.87			
			AW199824		1.86			
			BX852440		1.86			
			CF285710	GPT2	1.86			
			AW148075	BTG3	1.86			
			BX849426		1.86			
			BG730586		1.86			
			BJ061488		1.86			
			BC059286	SMAD7	1.86			
			BC068926	MAP2K5	1.86			
			BC089198		1.86			
			AJ555187		1.86			
			BC081238	SLC43A1	1.86			
			BC078089		1.86			
			BC042222	SLC7A3	1.86			
			BG019400	TLR1	1.86			
			BJ065916		1.86			
			BC070606	SSBP3	1.86			
			CB755810		1.86			
			BQ734261		1.86			
			BC085028		1.85			
			BJ093004		1.85			
			BJ628972		1.85			
			BX844538	LENG1	1.85			
			BE507287		1.85			
			BC097855	HNRPD	1.85			
			BJ090358		1.85			
			L20728	MR1	1.85			
			BC080107		1.85			
			BF231538		1.85			
			BC072285	CXXC1	1.85			
			BC077969	FBXL20	1.85			
			BP691913		1.85			

			BC046697	PRKACB	1.85			
			BP740251		1.85			
			CF548959		1.85			
			BC077480	MTHFD2	1.84			
			AY035397	SOX9	1.84			
			CK799833		1.84			
			AF545659	FLOT1	1.84			
			BP697688		1.84			
			BC097925	C11orf46	1.84			
			BJ622465		1.84			
			BC073570	ACTR10	1.84			
			BC082825	ZNF585B	1.84			
			BC084665	P2RY12	1.84			
			CF285027		1.84			
			BC077264	ETS2	1.83			
			BC043629	SDCBP	1.83			
			BC074357	PERP	1.83			
			BC046850	KNS2	1.83			
			CD811370		1.83			
			BI448805		1.83			
			BX854258		1.83			
			CO382517		1.83			
			BC078116	SEMA4C	1.83			
			BC086284	ZNFN1A4	1.83			
			BC084276	PDE5A	1.83			
			BC084298	TEX10	1.83			
			BJ088082	RNF130	1.83			
			CD301184		1.83			
			AW646443		1.83			
			BC046951	MAF1	1.83			
			BC068685		1.83			
			BJ074198		1.83			
			BJ085832		1.83			
			CF548617	DLG4	1.83			
			BC073407	GJA1	1.82			
			BJ619512		1.82			
			BC082869	PHF10	1.82			
			BC085058		1.82			
			BC084060	ELF2	1.82			
			BC089176		1.82			
			M29857	IGF1	1.82			
			BC044038	UBE2H	1.82			
			CN318629	POP1	1.82			
			BC084969	SSPN	1.82			
			BX853762		1.82			
			AW645391		1.82			
			BC073397	SLC38A3	1.82			
			BG234149		1.82			
			BC078062	HMG20A	1.82			
			BG730702		1.82			
			BM180394		1.82			
			AW765504	KIAA0460	1.81			
			AW200607	SEMA4A	1.81			
			AW633302		1.81			
			BC073426	SNX10	1.81			

			BG037930		1.81			
			U95104	CYYR1	1.81			
			BX844446	PPP1R14A	1.81			
			BC073346	PSMA5	1.81			
			CB560758		1.81			
			BG160137		1.81			
			BG160616		1.81			
			BP688728		1.81			
			BG162225		1.81			
			BJ644269		1.81			
			AF212299	TERT	1.81			
			BC072219	NPHP1	1.81			
			BP686071	DENND2C	1.81			
			S80988	RNF36	1.81			
			BX844526		1.80			
			AW199874		1.80			
			BX855177	ELL	1.80			
			BC084410	HEY1	1.80			
			BX851375	PPFIBP2	1.80			
			CD100831		1.80			
			CF283462		1.80			
			BC046733	SNAPC5	1.80			
			BC081160	ATP6V0B	1.80			
			CF270711		1.80			
			AW640455		1.80			
			BC077611		1.80			
			BM172522		1.80			
			CB559165	FCN2	1.80			
			BC044985	PPM1D	1.80			
			BJ040178	ZNF484	1.80			
			BC079779	MAPT	1.80			
			BC084962	ATP2A1	1.80			
			BC097538	PTPN6	1.79			
			BJ037429		1.79			
			BC075153	GLT8D1	1.79			
			BG161391		1.79			
			BX848237	PHC1	1.79			
			CF519782		1.79			
			BX853773	LY9	1.79			
			BG161111		1.79			
			BE491038	BRD1	1.79			
			AW200470	EMP2	1.79			
			BC044271	TARDBP	1.79			
			BC046954	DNAJA2	1.79			
			BC084882	OPN1SW	1.79			
			BE192088		1.79			
			CK797064	PSMB2	1.78			
			BX850387	REEP6	1.78			
			CV077651		1.78			
			BC082370	ARPC4	1.78			
			BC046649	ANUBL1	1.78			
			BC084310	CITED2	1.78			
			BJ069449	MSRB2	1.78			
			BQ398810		1.78			
			BC045080		1.78			

			BC072140		1.78			
			BC080419	IFT52	1.78			
			CK798049		1.78			
			BE679052	EIF5B	1.78			
			BC079722		1.77			
			BJ030478		1.77			
			BG162413		1.77			
			CD255626		1.77			
			CF271607		1.77			
			BC070610	ZNF499	1.77			
			BI313886	KLHL7	1.77			
			BP740064		1.77			
			BC086459	SUV420H1	1.77			
			BG163133	SOSTDC1	1.77			
			BC079769		1.77			
			BC076870	SNX10	1.77			
			BI349874		1.77			
			CF549211		1.77			
			BC054198	ARPC2	1.76			
			BP677659		1.76			
			BC088802	DAPK2	1.76			
			BF613334		1.76			
			BX845200		1.76			
			BC073479	PITX2	1.76			
			BC061681	HLA-DMA	1.76			
			BC073475	SESTD1	1.76			
			BP696833	RIC8A	1.76			
			BC059964	C1orf80	1.76			
			BC073652	CCT4	1.76			
			BC082903	TSC22D3	1.76			
			BC084797	GNB4	1.76			
			BC076837	RNASET2	1.75			
			X68024	ZFX	1.75			
			CA974036		1.75			
			BC054220	TPM1	1.75			
			BP699596		1.75			
			BP733644		1.75			
			BC072852	SURF5	1.75			
			CD328776		1.75			
			BG555382	PITRM1	1.75			
			CB559498		1.75			
			BC081054	HDAC2	1.75			
			BC070558	ITGB1	1.75			
			BC077294	RBBP5	1.75			
			BC079698		1.75			
			BC045128	NONO	1.75			
			BC054167	SEC11L1	1.74			
			BC054976	UBC	1.74			
			BJ039130		1.74			
			BQ731162	ZNF592	1.74			
			BC045129	CDC34	1.74			
			BC075190	EIF3S4	1.74			
			BJ636429	SMAD1	1.74			
			BC073675		1.74			
			BJ068253	C3orf38	1.74			

			BC046661	SFRS3	1.74			
			BF427906		1.74			
			BX845493		1.74			
			BX847278		1.74			
			CA981557		1.74			
			CA981988		1.74			
			BC070768	KLHL7	1.74			
			BC081245	COPS8	1.74			
			BC090223		1.74			
			BC084342	SRPK2	1.74			
			BC081129		1.73			
			BI478201		1.73			
			BE680744	DNMBP	1.73			
			BP724847		1.73			
			AJ010503	PAX5	1.73			
			BC054982	NCALD	1.73			
			CF288359		1.73			
			BC055994	CASC4	1.73			
			CF342308		1.73			
			BC077554	C21orf59	1.73			
			BC073576	RSU1	1.73			
			BF427961		1.72			
			AJ243591	PCBP2	1.72			
			BC073511	VPS72	1.72			
			BC084806	SCG2	1.72			
			BC088689		1.72			
			BF049078	CTDSPL2	1.72			
			CA983533	PAPSS1	1.72			
			AW635951		1.72			
			BC045260	HNRPA1	1.72			
			BC071021	CYR61	1.72			
			BG514340		1.72			
			AB059563	BMP3	1.71			
			AF166261	CCDC18	1.71			
			BC070649	RBMXL1	1.71			
			CX135377		1.71			
			CK797066		1.71			
			AY781409	TRIM33	1.71			
			BC042314	VPS4A	1.71			
			BI441614		1.71			
			BJ088746		1.71			
			BJ088674		1.71			
			AW646471		1.71			
			BC084404	CSRP3	1.71			
			BC074283		1.70			
			BC074455	SCRN1	1.70			
			BG017718	TMSL3	1.70			
			BG018724		1.70			
			CA980763	ZNF484	1.70			
			CK798514		1.70			
			BC082353	OGT	1.70			
			BC044983	PSMA4	1.70			
			BE679591		1.70			
			BC075188		1.70			
			BC081165	GBA	1.70			

			BC094169	SHANK2	1.69			
			BP710184		1.69			
			BQ736615		1.69			
			AF012925	VENTX	1.69			
			BC089148	PRSS16	1.69			
			CD100547		1.68			
			BJ092419		1.68			
			BC054981	TEF	1.68			
			BP709456		1.68			
			BJ617051		1.68			
			CX130081	FBXW7	1.68			
			BC082661	CYSLTR1	1.67			
			BC072990	DGUOK	1.67			
			BC061671	CPNE1	1.67			
			BC070537		1.67			
			BJ616085		1.67			
			BC068709		1.66			
			BP704246	C9orf40	1.66			
			BP707937		1.66			
			BJ625917		1.65			
			AY149119	PAQR8	1.65			
			BM262060		1.64			
			BX844131		1.64			

Supplemental Table S3B: Genes down-regulated during natural development

Note: All the HUGO symbols are derived from predicted human homologs

Stage 58 v Stage 53			Stage 61 v Stage 53			Stage 66 v Stage 53		
**GenBank**	**HUGO**	**Fold Change**	**GenBank**	**HUGO**	**Fold Change**	**GenBank**	**HUGO**	**Fold Change**
AB075925	OLFM4	0.017	BC082684	APOA4	0.007	BC044116	KRT8	0.005
AB022088	CYP1A1	0.078	BC047973	GATM	0.012	BC085055	ANPEP	0.018
BC086297		0.082	BC080108	ELOVL2	0.012	BC082530		0.028
BC042305	SLC22A6	0.086	BC078533		0.017	BC044073		0.033
BC078522		0.088	BC045001	GAMT	0.017	BC074222	SLC16A12	0.036
DR728320		0.098	BC086290		0.018	CK799950		0.049
BC077417	F11	0.128	BC077556	FADS2	0.020	BJ057663	AMID	0.055
BC073008	DNASE1L3	0.131	BC078522		0.021	BC072842	TRIM2	0.067
BC076771	FRAT1	0.150	BC054984	ABHD6	0.024	AY762616		0.068
BC060331	G6PC	0.151	AW646661		0.025	BC042305	SLC22A6	0.068
BC074120		0.157	BC061932	AKR1D1	0.026	BC056840	LTF	0.071
BC073507		0.161	BC044073		0.026	BC060496	CYP3A4	0.072
BC045001	GAMT	0.165	BC086297		0.027	BC081224		0.082
BC073297		0.169	BC048227	PCK1	0.028	DQ096886	CA14	0.083
BJ057663	AMID	0.170	BC074120		0.028	BX844453	DIO1	0.088
AW641041		0.172	BC071092		0.029	L20816	PLCB3	0.092
BP687505		0.173	BC074322	TMEM56	0.030	BC086270		0.097
BC087377		0.174	BC076843	NPPC	0.032	BC063726	FADS2	0.101
BC079680	SHMT2	0.178	BC076743		0.033	DR728320		0.103
BX845249		0.181	BC084618	CYP4B1	0.033	BX843674	ABO	0.106
CD099668	VNN1	0.183	U76636	CALB1	0.033	BC056128	WNT5B	0.109
BC082696		0.184	BC077457	QPRT	0.035	BC082934	CYP3A4	0.111
BC043748		0.185	BC072977	PAFAH2	0.036	AB022088	CYP1A1	0.112
BC060496	CYP3A4	0.191	BC084855		0.036	BC045001	GAMT	0.112
DQ096886	CA14	0.194	CK799015		0.037	BC082652		0.114
CK799015		0.201	CD099668	VNN1	0.039	BC059306	SLC45A3	0.115
BC059968	AADAT	0.203	BC054960	VIL1	0.044	BC056067	EDAR	0.117
AW645021		0.205	BP704434		0.045	BC056046	NNMT	0.124
BC046739	ACTC	0.208	CD301370	ELOVL5	0.046	BC077065		0.125
BC082839		0.209	BQ727333		0.046	BC054222	CYP46A1	0.130
BC044073		0.233	BP707243		0.047	BC041315	CPA3	0.135
D49543	INHBB	0.238	BC068755	ACAT1	0.047	BC077437		0.135
BC072360	RAB30	0.244	X93494	GCK	0.048	AY648303		0.139
AW646661		0.250	BC060496	CYP3A4	0.048	BC041213	CA2	0.141
AF184090	ADFP	0.257	BC054239	TM4SF4	0.048	BC084838	GSTO1	0.143
BC056038	DSCR1	0.260	BC087294		0.049	BC077457	QPRT	0.145
BC056046	NNMT	0.263	BC054307	STOM	0.051	CK799015		0.147
BP707243		0.271	BC042305	SLC22A6	0.054	BC084127	IGFBP1	0.151
BC073632		0.277	BC073008	DNASE1L3	0.055	BC073507		0.153
BC074191	GCHFR	0.279	CD325272		0.057	CB561838		0.153
BC056067	EDAR	0.283	BC041754	FGA	0.057	BC084855		0.158
BC084783	CIDEA	0.284	BC072842	TRIM2	0.058	BX849284		0.161
AF232672	FKBP10	0.289	BC074303	GRTP1	0.059	BC071092		0.168
BC054225	HSPB8	0.294	BC087377		0.060	BC061650	TAGLN	0.171
AY689185		0.312	BC077436		0.061	BC077417	F11	0.179
Z95080	HAND1	0.319	BC089165		0.063	BC046378	HYAL2	0.180
AB059564	GDF10	0.320	AY762616		0.067	BJ067458		0.191
BC047973	GATM	0.332	AB114053		0.071	CF288050	C6orf85	0.193
BC060415	PAPSS2	0.333	BC081224		0.071	BC081074		0.204
BC074488		0.335	BQ737008	FAAH	0.072	BC043819	IRAK2	0.205
BC077824	CALU	0.336	CD100048		0.073	BC083003	ADH1B	0.207
AW200257	PEMT	0.337	BC063726	FADS2	0.074	X93494	GCK	0.216
BC054153	GLUL	0.340	AB022088	CYP1A1	0.080	BC079744		0.219
CK799950		0.341	BC087494		0.080	BC047247	KCNK6	0.222

BG016864		0.356	BC046711	SDFR2	0.082	BM179237	SYN3	0.231
BE507722		0.361	U05001	NR5A2	0.082	CD302379		0.237
BC078077		0.362	BC090224	SULT1E1	0.082	CD328086		0.238
BC059979	RHBG	0.364	BP699428	EPHA7	0.082	BM928974		0.244
BC043628	CHRND	0.367	BC054291	HSD11B1	0.082	CK800173		0.246
AY762616		0.368	BP687505		0.082	BC054153	GLUL	0.251
BX843339	EIF2AK2	0.370	BC081141	RDH5	0.083	BC060331	G6PC	0.255
BX848856	INHBB	0.370	BC079789		0.084	BC081130	C1orf139	0.259
AY303832	CAP2	0.371	BC074127		0.085	AW641055	PCTK2	0.261
BC077819		0.372	CV121162		0.087	BC054253	FAM82B	0.263
BC070544	TMEM45B	0.373	BC061667	APOA4	0.087	BC072808	ARID3A	0.265
BC068878		0.373	AY197550		0.087	BC088706		0.270
BC068755	ACAT1	0.378	BC070782	MYO1B	0.088	BC078106	ZFYVE28	0.274
BC073422	ANXA6	0.378	BC074131	CYP4V2	0.090	BC078055	SC5DL	0.275
BC068972		0.381	BC081210		0.093	BC072804	TMEM34	0.278
BC086290		0.383	BC075136	DHRS4	0.095	BC059979	RHBG	0.278
BC046856	EIF4A2	0.384	DQ096886	CA14	0.095	BG731178	CA4	0.279
BJ039830		0.386	BG022822		0.096	BC074377	JUN	0.282
BG037969	SNX3	0.386	BX848543		0.097	BC079982		0.289
BC086266	ADAMTS1	0.389	BC061682	ADH1C	0.099	BC076838	CRY1	0.290
AF358869	WEE1	0.391	BC084384	FA2H	0.099	BC054307	STOM	0.291
BQ736745		0.392	AW641041		0.100	BC068700	STARD10	0.293
BC076843	NPPC	0.392	BC077479	CYP4B1	0.100	BC084783	CIDEA	0.294
BC059963	BACE2	0.393	BC043756	ACSL1	0.101	AB059564	GDF10	0.296
BC072803	SMYD1	0.393	BC077417	F11	0.103	D86492	IRF6	0.299
BC081238	SLC43A1	0.396	BC084850		0.103	BC043628	CHRND	0.299
BC082481		0.396	BC072281	TSPAN13	0.105	BQ733454		0.302
BP706536		0.396	BC042356	STOM	0.105	BC056847	CYP2C8	0.305
BG731178	CA4	0.402	BJ041689	CLEC10A	0.107	BP707684	CAPN2	0.307
BC073613	F7	0.403	BC056067	EDAR	0.107	BC068755	ACAT1	0.309
BP674987	POR	0.406	BX843538	WBSCR17	0.107	BC044675	ENPP2	0.316
AB030502	UBQLN4	0.410	BC046694	RDH16	0.107	BG017589		0.316
BQ727333		0.412	BC054987	NAALADL1	0.108	DQ096919		0.316
BC081074		0.413	BC084078	C9orf52	0.108	BE507722		0.316
CV073503		0.413	BC079694	HMGCS1	0.109	BC068748		0.322
BC068729	RBM7	0.416	BC082652		0.109	AF263272	CDH6	0.322
BC041213	CA2	0.416	BC076826		0.112	BG019049		0.329
AF388036	BOC	0.418	AF184090	ADFP	0.112	CD301370	ELOVL5	0.335
BC081210		0.419	BC054281	HNMT	0.113	BJ062152		0.336
X74315	FOXI1	0.419	BC054207	DEGS2	0.113	BC042356	STOM	0.337
BC049292	COMT	0.421	BG037969	SNX3	0.114	BC070544	TMEM45B	0.338
BX852437	MFSD2	0.423	BC077437		0.114	BC083004	LONRF1	0.339
BX852927	SEC11L3	0.425	CK805431		0.116	BC084850		0.340
BC072380	POLR3D	0.426	BC060486	SLC37A4	0.117	BC059997		0.342
BP691809		0.426	BC078055	SC5DL	0.117	BQ386792		0.344
BP698942	MRPS23	0.428	BX845249		0.117	CB756001	LCT	0.345
BC059997		0.430	BC041234	PISD	0.117	BC060473	SLCO2A1	0.346
CD325272		0.431	CK803937		0.117	BC044982	SLIT1	0.348
BX846825		0.433	D49543	INHBB	0.118	BC053785	SLC31A1	0.351
BC084860	ME3	0.433	BC079736		0.119	BC074387	PCSK2	0.354
CD301370	ELOVL5	0.435	BC085208		0.119	AW199610	RNF152	0.354
BC084265	HMBS	0.436	BC079744		0.120	BC077836	UAP1	0.355
CV121162		0.437	BC082372	CIDEC	0.121	BC072823	SESN3	0.356
BC077458	SF3B4	0.438	CD326519		0.121	BC073572	NR1D1	0.359
BJ063969	PDLIM3	0.439	BC081049		0.122	BG234364		0.359
BJ086892		0.440	BC060331	G6PC	0.124	BC081141	RDH5	0.361
CF520476	FAM3B	0.440	BC056036	CLIC5	0.126	BP742091		0.362
CF522107		0.441	BC061656	SAR1B	0.126	BC061655	PLS3	0.364
BC087294		0.442	AF143489	ACAT2	0.126	X88927	NOLC1	0.369

BC077820	FBXW5	0.444	BC072340		0.126	AF146087	HES5	0.371
BC082891	CIDEA	0.449	BC041303	SLC25A1	0.129	CD302763	MLH1	0.372
BC082395	PPP1R14A	0.450	BC053785	SLC31A1	0.129	BC044326	NEK6	0.374
BC060486	SLC37A4	0.451	BC073246		0.130	BC082891	CIDEA	0.375
BC077437		0.451	BC070544	TMEM45B	0.131	AY354455	NCK2	0.376
BC088706		0.454	CK800673		0.131	BC076771	FRAT1	0.377
BC068745	SESN2	0.455	BE576565		0.131	CB944053		0.382
BC080019		0.455	BQ726613	SLC39A5	0.131	BQ726537	MUC5B	0.382
BQ386792		0.456	BC042929	HMGCS1	0.132	BG037998		0.384
BC043741	FKBP9	0.457	BC081057		0.134	BC041234	PISD	0.384
BC055967	EBP	0.457	BC088706		0.135	AJ007835	AHCY	0.384
D86492	IRF6	0.458	BC045267	PALMD	0.137	BX851573	MGAT4B	0.388
BC073130	NIF3L1	0.461	BC059979	RHBG	0.137	BC076843	NPPC	0.390
AF527799	BVES	0.465	BC046739	ACTC	0.137	CA972804	E2F5	0.391
BC072910	KAZALD1	0.467	BC072036		0.137	AW200247	MIXL1	0.393
BC054220	TPM1	0.469	BC082351	ADFP	0.138	BC090198		0.393
BC081192	MRPL2	0.472	BC078066	EPHX2	0.139	BC073014	BXDC2	0.395
CF286689		0.473	BX844453	DIO1	0.141	BJ635358		0.396
BC086477	IRS2	0.474	DR728320		0.141	BC072927	PIPOX	0.402
BC080437		0.474	BC082648	IDI1	0.142	X74315	FOXI1	0.403
BC042276	SHMT1	0.475	BC073400	AHCY	0.144	DR729572		0.405
BC056121	FARSLB	0.476	BC059968	AADAT	0.145	BC072223	BYSL	0.407
BC077216	PHB2	0.478	BC068707	LAP3	0.147	CB563002		0.409
BC060361	TKT	0.478	CK799950		0.148	BC059961	HABP4	0.410
AB026192		0.480	U17608	ABCB1	0.148	BC054957	CD63	0.411
M55163	FGFR1	0.481	CK799249	GSTZ1	0.148	BC072340		0.411
CK800472		0.482	BC060346	SDSL	0.148	BC073384	ASMTL	0.411
DR729572		0.482	BC084341	FDXR	0.150	CA983342		0.413
BC082401	DPP4	0.483	BC075178	SLC31A1	0.151	CK800517		0.414
X69662	GSS	0.484	BP683015		0.152	BC073400	AHCY	0.418
BC084325		0.484	AJ007835	AHCY	0.152	BC072989		0.420
BC082934	CYP3A4	0.486	BC068798	TDRD7	0.153	BC060398	RIOK3	0.421
AB037936	RGN	0.487	BX852007		0.154	AF184090	ADFP	0.423
BC082372	CIDEC	0.487	BC082934	CYP3A4	0.155	BC077990	SLC38A2	0.423
U88065	ADAR	0.488	BC078068	RHOU	0.155	BG161411		0.423
BC054283	FAH	0.489	BX850024	HSD17B6	0.156	BC077606	PPOX	0.424
CK800173		0.489	BC077065		0.156	BC068845	NOLA2	0.424
BC073032	EEFSEC	0.490	BC076754	MPST	0.158	CK815991	FLJ20397	0.424
BC083037	GRHPR	0.490	CB944053		0.158	BC072976		0.425
BC082648	IDI1	0.491	BC054299	ACAA1	0.158	BC077582	TLE1	0.425
BC072331		0.491	BC054309	PRDX6	0.158	BC088699		0.425
BX850234	BET1	0.492	BC056840	LTF	0.158	BC046856	EIF4A2	0.425
BP693983	MVK	0.493	BJ062152		0.160	CV073503		0.426
BC044971	SLC7A8	0.494	BC054315	MYBPH	0.161	CX132821		0.426
BE132270		0.496	BC045003	IRF6	0.162	BC068696		0.429
CV076731		0.496	BC077507	PHYH2	0.162	BP698942	MRPS23	0.429
BI314985		0.499	AY354455	NCK2	0.163	BC074446	NT5C2	0.430
BC044112	PDK2	0.500	AY362041	SOD2	0.164	CK797932	PC	0.431
BF614590	DNAJA3	0.500	BC074145	HSD17B4	0.164	BC081210		0.431
BC073664	ALAD	0.501	BC070657	ABCD3	0.167	AW764914		0.432
BC043819	IRAK2	0.502	BC056046	NNMT	0.167	BQ737008	FAAH	0.433
BC068714	MAD2L1	0.503	CK800472		0.167	CK805115		0.433
BC042288	CDC20	0.503	BC097581		0.168	BC074463	FCN2	0.433
BC056131	TRIM16	0.503	BC084237		0.170	CA982667		0.434
BC077963		0.504	BC068666		0.171	U45453	GATA4	0.434
BC081014	GTSE1	0.505	BG019049		0.172	BC044007	PKM2	0.434
BC071096	METT10D	0.506	CD302379		0.174	BC097862	WDR50	0.435
BC042228	FZD7	0.507	BQ733454		0.174	BC073021	CCDC58	0.435
BC090198		0.507	BP709638		0.174	AJ585762	ST3GAL2	0.435

BC081043	AZIN1	0.509	BC082705	DDT	0.174	BC068695	WARS	0.436
BC082939		0.509	CK800517		0.175	BC086470	KLF10	0.436
CD255149		0.510	BX848856	INHBB	0.175	BC061648	TSPAN3	0.437
BC084949	TIMM22	0.511	BC074152		0.175	BC084265	HMBS	0.437
BC082705	DDT	0.511	BC054229	APOB	0.178	X12499	HOXC6	0.437
BP690860		0.511	BC076642	PSPH	0.179	BC082875	RBM19	0.439
BC084848	TAGLN	0.512	BC073507		0.179	BC048226	C17orf40	0.441
BC087401		0.513	BC084255		0.179	BC087309		0.441
BC072276	KPTN	0.517	BC070665	SLC25A10	0.180	BF611360		0.441
BC084801		0.518	AW200202	CASP7	0.180	AW782935		0.444
BX844946		0.519	BC054290	GSTK1	0.181	CX130840		0.445
BC054152	SQRDL	0.520	BC074206		0.181	AW646316		0.445
BC068968	MASTL	0.520	BC046740	ACSL1	0.181	BC068798	TDRD7	0.446
BC061655	PLS3	0.521	BC063727		0.182	BC070789		0.447
BQ884420	ARHGAP17	0.521	BJ065503	GJB1	0.184	BX853035		0.448
BC078029		0.522	BC082361	RHOU	0.184	BC073613	F7	0.448
BC044995	DHCR7	0.522	BJ636812	SYCP2	0.185	BG160506		0.450
CF288031		0.523	BC082471		0.186	BC068856		0.452
AJ007835	AHCY	0.526	CA983342		0.187	BX853482	CD3EAP	0.453
BC070692	SRM	0.527	BC073116	B3GNT7	0.188	AF417472	MYBPC3	0.454
BC084828	MRPL12	0.527	BC088717		0.188	AW641041		0.454
BC076752	GLO1	0.527	BC088905	ALDH3A2	0.188	BC077799	MED8	0.455
BC081144		0.528	BC070704	SLC2A2	0.189	BQ736745		0.456
BQ724783		0.531	CK798124		0.190	BC077210	ACADM	0.456
BC070821		0.532	BC059301	KCNJ15	0.192	AB003358	LYN	0.456
BC082429	DMD	0.532	BC074458	C9orf64	0.193	BC068895	KIAA0859	0.457
CB563002		0.532	BC074465		0.193	BC068940	CDH1	0.457
BP709301		0.532	BC060415	PAPSS2	0.193	BC073722		0.458
CF549269		0.533	BC072788		0.194	BC081246	LRRC22	0.458
CX132821		0.533	BC059991	DGAT2	0.194	BC042245	GLDC	0.459
BJ068483		0.534	BG731178	CA4	0.194	BC084936	BLNK	0.460
BJ643315	CRBN	0.535	BC077572		0.194	BC074191	GCHFR	0.461
CK798087		0.535	AY648303		0.195	BP674987	POR	0.462
BC068895	KIAA0859	0.535	BC063733	CAPN2	0.196	BC070652	PQLC1	0.464
BC060449	EPB41L3	0.536	BC059997		0.198	BG162399		0.464
BC082884	C10orf22	0.536	BC071002	AADAT	0.199	CK797414		0.465
BJ636812	SYCP2	0.537	CA981199	HSD17B8	0.199	BC077917	LTB4DH	0.466
BC082400	USP14	0.537	BC085048		0.199	BC073297		0.467
BX845098	MRPS31	0.538	BC079708		0.200	BC070692	SRM	0.467
BC071128	CBR1	0.539	BC081075	SLC6A19	0.200	BP701638		0.467
BC087450		0.539	BC084269		0.201	BC056121	FARSLB	0.468
BC044121	DOK4	0.540	AB008225	SLC5A11	0.201	BC082481		0.469
BJ055719		0.540	AW200549		0.201	BC084066	CDC2L1	0.470
BC071140	MTX1	0.541	BC073248		0.201	BC081042		0.470
AF036617	FECH	0.541	BC060449	EPB41L3	0.202	BC082939		0.473
AY534752	CDC14A	0.541	CA973597	SCP2	0.203	BM192882		0.477
BC082480	TATDN1	0.541	BC076646	PHYH	0.204	AW635120		0.480
BC054181	KDELR3	0.544	BC082381		0.204	BF615462	RABL5	0.482
BC068798	TDRD7	0.545	BC077917	LTB4DH	0.204	BX843538	WBSCR17	0.482
BX842761	PRSS15	0.545	CK804949		0.204	BC084773	TLR5	0.483
BC082409	SLC25A26	0.545	BC071106	ALDH3A2	0.205	BX849962	PTPRR	0.483
BC079713	LARS	0.546	BJ616136	B3GALT2	0.205	BC070683		0.484
BC073000		0.548	AY689185		0.205	BC068661		0.484
CA972414		0.549	BC042276	SHMT1	0.206	BJ636812	SYCP2	0.485
BC076789	CHRNA5	0.549	CK796641		0.206	BG016864		0.489
BC073217	NDUFB7	0.549	AW871903	PRRT1	0.206	AF042353		0.489
BC077210	ACADM	0.551	BC072909		0.208	CK800700		0.489
BC073058	SMYD5	0.551	AF146087	HES5	0.208	BC056000	PRSS27	0.491
BC073400	AHCY	0.555	BC043834	SLC25A25	0.210	AW200398		0.492

BP684778		0.555	BC082696		0.210	BC084278	NOC3L	0.493
BC072179	PDLIM7	0.556	BC077486	TMEM16E	0.210	AY095442	BUB1B	0.494
CD101209		0.557	BC073341	DHRS7	0.211	BC082648	IDI1	0.494
BC070595	C15orf41	0.558	BG017589		0.211	BC077206	PDCL3	0.494
BC077929	AOC3	0.559	BC077984		0.212	CA974265		0.495
X53962	CDK7	0.559	BC080008		0.213	BC087487	CETP	0.496
BC077224	KIF20A	0.562	BC078570	PEMT	0.213	BC072977	PAFAH2	0.496
BC045127	CYC1	0.563	BC076718	GATA6	0.214	BC068809		0.497
BJ055092		0.564	CB756001	LCT	0.214	BC084674		0.497
BC072036		0.564	BC084322		0.215	BC081014	GTSE1	0.498
BC056845	ACAD8	0.569	BC078134		0.216	BC092390		0.499
BC068951	RPIA	0.583	BC088918	GRAMD3	0.216	CF547931		0.499
BJ624120		0.592	BX843339	EIF2AK2	0.216	BC072376	AP1M1	0.499
			BC089129		0.216	BC084264	DHX32	0.500
			BC094271	QDPR	0.217	BC080095	C3orf26	0.501
			AF061833	ALDH1A1	0.218	BQ736389	SMN1	0.502
			BC077210	ACADM	0.218	CD325272		0.502
			BC077580	B4GALT5	0.218	BC078028	C1orf93	0.502
			BC046710	ABHD3	0.219	CF549269		0.504
			AJ575933	FUT2	0.219	BC068703		0.505
			BE507722		0.220	BC068878		0.505
			BC074197	HMGCR	0.220	BC072067	PMPCB	0.506
			CK796536	MGST1	0.220	BC047972	WDR36	0.506
			BC087621	DERA	0.220	BC059972	PDK2	0.506
			BC088699		0.221	X69662	GSS	0.506
			BJ067458		0.223	BC087453		0.507
			BC080065	CDR2	0.223	CD328604		0.507
			CK799885	MGST3	0.223	CK798087		0.507
			CD328086		0.223	BC070576	MPP6	0.508
			BC073297		0.225	BC092017	CYB5B	0.508
			BC070671	SLC34A2	0.225	BC068745	SESN2	0.509
			BC081062	GCSH	0.226	BC077562	MINA	0.509
			BQ736745		0.227	BJ094084		0.510
			BC073572	NR1D1	0.228	BC073712		0.510
			AW767401	DBI	0.228	BX852494	C2orf17	0.511
			BC084936	BLNK	0.229	CF522107		0.512
			BC075133	ECH1	0.229	AW164964		0.513
			BC063272	SLC25A22	0.229	BX845098	MRPS31	0.514
			BC049292	COMT	0.229	BQ725721		0.515
			BC041742	B4GALT2	0.230	BC073430	NMD3	0.515
			BC084667		0.231	AF272361	EBNA1BP2	0.515
			BC084264	DHX32	0.231	BC073130	NIF3L1	0.515
			BX849020	STARD4	0.231	BC043879	ZNF622	0.517
			BC074429		0.232	BC092020		0.518
			AW645021		0.232	X53962	CDK7	0.518
			BC082655	SCP2	0.233	BC082380	PPID	0.518
			BC045119	ACAA2	0.233	BC054239	TM4SF4	0.519
			BC059990	CYP2E1	0.235	BC072199	SRPK1	0.519
			BC060444	PRKAG2	0.235	BC074325		0.519
			BC077325		0.235	AY676874		0.520
			AF182522	PKP3	0.236	BC043994		0.520
			BC078518	CRYM	0.236	BE679747		0.520
			BG409617		0.236	CB565452		0.520
			BC075155	ADK	0.236	BX844221		0.521
			BJ052687		0.237	BC068757	GLTP	0.522
			BC077334	CYB5A	0.237	BC070738	CENTG2	0.522
			BC072727	PGRMC1	0.238	BC088708	NUBP1	0.522
			BC045269	GOT1	0.239	AW200624		0.523
			BC068745	SESN2	0.241	BC043624	PPP2R1A	0.523

			BC071136	DECR2	0.242	BX855102	C5orf15	0.524
			BC081124	IPMK	0.242	BC082640		0.524
			BC074446	NT5C2	0.243	M96858	CDC25C	0.525
			BP674987	POR	0.243	BC074496	PRAF1	0.525
			BC077769		0.243	CK742103		0.526
			BX849284		0.245	BC042276	SHMT1	0.526
			BC042245	GLDC	0.246	BJ068336	NEK6	0.526
			BC080003	DPYD	0.246	BP684433		0.526
			CF520476	FAM3B	0.246	BC082395	PPP1R14A	0.527
			CK799201		0.246	BC088960	SGTA	0.528
			CA982667		0.247	BC090160	C14orf32	0.528
			BC059318	POR	0.248	CF521010		0.528
			BC072803	SMYD1	0.248	AF270484	TKTL2	0.528
			BC079981	OACT5	0.249	BC075155	ADK	0.529
			AB176536	LRRC54	0.250	BC088717		0.529
			BJ039830		0.250	BC077415	EXOSC7	0.530
			BP725976		0.251	CF290590	PPP1R15B	0.531
			BC046688	SLC7A8	0.251	BX847425		0.532
			BC045088	SERHL2	0.252	BC087621	DERA	0.532
			DQ097000		0.254	BJ631732		0.532
			BP706037	COX6C	0.255	BC073463	DNAI1	0.532
			BC076860		0.258	BC073227	TSEN2	0.533
			CF521010		0.258	AY534752	CDC14A	0.534
			CF521207		0.258	BC077826		0.534
			CA982252		0.258	BC087459		0.534
			BC084761	SLC35F2	0.258	BC079784	TMEM48	0.535
			BC047967	SLC16A6	0.258	BC060355	GAS6	0.535
			BC073240	MYO1D	0.259	BQ726603		0.536
			BC071113		0.260	BC086273		0.537
			CA983600	ALDH6A1	0.261	BC059354	TBL3	0.538
			BC077532	PECR	0.262	AY164461	SFRP5	0.538
			CD100577		0.263	BC075175	SCRIB	0.538
			BC054964	CAT	0.263	BF072222	WDR12	0.538
			Z49827	HNF4A	0.263	BC074238	RAB6A	0.539
			BC074162	HSD17B12	0.264	AY731504		0.540
			CB563002		0.264	BC077216	PHB2	0.540
			BJ083148		0.264	BJ616136	B3GALT2	0.540
			BC081043	AZIN1	0.265	BJ094257		0.541
			BC055967	EBP	0.266	CF520476	FAM3B	0.541
			BC082902		0.266	AB176537	LRRC54	0.543
			BC041213	CA2	0.267	BC077322	TSEN54	0.543
			BC089134		0.267	BC077792	CSF3R	0.544
			BF025190	DPP4	0.268	BC084984	CEBPZ	0.544
			BX853035		0.268	BX851821		0.544
			BC074201	DHDH	0.268	BC097518	TIMM10	0.545
			BJ086892		0.269	BG810957		0.545
			CB756731		0.269	BQ398814	ZNF365	0.546
			AJ585762	ST3GAL2	0.269	CK805195		0.547
			BX848535		0.269	BC072749	EXOSC9	0.548
			BC059978		0.270	AW200034	RNU3IP2	0.548
			BM179769		0.270	CO386298	CKS2	0.548
			BC073664	ALAD	0.270	BP682407		0.549
			BC074319		0.270	CV121162		0.549
			BC053792	SULT1C2	0.271	CN327872		0.549
			BC085020	SLC25A20	0.272	BC045248	GNL3	0.550
			BC054253	FAM82B	0.272	BC097649	WDR51B	0.550
			BE576109		0.272	BQ734449	HSPA14	0.550
			BC055995	OXNAD1	0.272	BC081085	AGPAT1	0.550
			BC082357	NUDT4	0.274	CK798599	PRDX2	0.550

			BC077247		0.274	BC079921	PKM2	0.552
			BX851573	MGAT4B	0.274	BF610767		0.552
			BJ094257		0.275	BC059311		0.552
			AW200247	MIXL1	0.275	BC077397	NUP88	0.552
			BC084266		0.275	BC077507	PHYH2	0.553
			AW635120		0.276	BC087401		0.554
			CA982427		0.276	CV074563		0.554
			BC077977	HADH2	0.276	BC099015	IBRDC1	0.554
			BC076857	PANK3	0.277	BJ616292		0.554
			BC074198	ASRGL1	0.277	BC045096	NCLN	0.556
			BM179237	SYN3	0.277	AW200257	PEMT	0.557
			BC082651	IDH1	0.278	BC072263		0.557
			BC084981		0.278	BP705037		0.558
			BC073653	SLC29A2	0.279	BC073574	SCCPDH	0.558
			CK806759	TMEM4	0.279	BG023328		0.558
			BP717523		0.279	CV076731		0.559
			BC072823	SESN3	0.280	BC068692	C14orf111	0.560
			BC073503	CIDEB	0.281	BC073197		0.560
			AW200411		0.281	BC073458		0.560
			BC083016	SREBF1	0.282	BC084408		0.560
			BJ631732		0.282	U36486	KIF2	0.562
			BC082387	C10orf58	0.283	BC045088	SERHL2	0.562
			CA973541	OCIAD2	0.285	BC076746		0.562
			BC046724	M6PRBP1	0.285	BC072214	DDX47	0.563
			BC082394		0.286	BP691865		0.563
			BX846352	FDX1	0.286	BX842842	AGPAT5	0.563
			BC077756	PFKM	0.287	CK796680	FNTA	0.564
			BC074191	GCHFR	0.287	AW200118		0.564
			CK797657		0.287	BC088915	RXRG	0.566
			BC057298	DPP4	0.288	CA981840		0.566
			BC078118	STRBP	0.288	AW199681	ME2	0.566
			CK800436	COX5B	0.289	BC073116	B3GNT7	0.566
			BC085065	NUAK2	0.290	BC074445	CACYBP	0.566
			CA974238	ATP5G3	0.291	BC079782	JUND	0.567
			BC061652	HSD11B1	0.291	BC077638	BXDC5	0.569
			BP682407		0.292	Z14253	ETF1	0.569
			AB059564	GDF10	0.292	BC070870	LRPPRC	0.570
			BC073379	OSBPL2	0.292	BC068907	DDX18	0.571
			BE508558		0.292	AW200411		0.571
			CA973430	BAIAP2L2	0.293	BC081043	AZIN1	0.571
			BG161411		0.295	BC079713	LARS	0.572
			BC045121	PHB	0.297	AJ557446	KPNA2	0.572
			BC056018	GPSN2	0.297	BQ884366		0.573
			BC072287	PCK2	0.297	BX849523	DCP2	0.573
			BC075226		0.298	AW199690		0.574
			BE507901		0.299	CK806759	TMEM4	0.574
			BC082653	CTH	0.300	BC073217	NDUFB7	0.575
			BC054278	PRDX6	0.300	BC073536	NFATC1	0.575
			BC083003	ADH1B	0.300	BC046945	BXDC1	0.576
			AW199835		0.301	AF055588	MGP	0.576
			CK797953		0.302	BE679568		0.576
			BC075171	CNDP2	0.302	BC073492	C21orf70	0.576
			BC061936	SYK	0.302	BC084310	CITED2	0.577
			BC081056	ACADM	0.303	BC059336	ASAH1	0.577
			BX845098	MRPS31	0.303	BC060455	CYP2C19	0.578
			BC082349	GATA6	0.304	X82012	KIF4A	0.578
			BE506980		0.304	BX842712	ZNF179	0.579
			CV523300	HOXA9	0.304	BC044113		0.579
			BC084329	HIBADH	0.304	BC084092	KIAA1008	0.580

			BE507967		0.304	BC072210	HRB2	0.581
			BC059972	PDK2	0.304	CK799932	GRPEL1	0.581
			BJ057663	AMID	0.304	BQ734937		0.581
			BC059961	HABP4	0.304	AF036617	FECH	0.584
			BC077788	SDF2L1	0.305	BC054996	SNX2	0.584
			BC084752		0.305	BC076784		0.585
			AW199610	RNF152	0.306	BP697564		0.586
			BF614568		0.306	BC092018		0.590
			BQ884420	ARHGAP17	0.307	BJ061090		0.590
			CK799532	USP21	0.307	AW199535		0.592
			CF547537	CHDH	0.307	BG811183		0.597
			BP692333		0.307	BX854027	LBR	0.597
			BC076840	PGM2	0.308	AW200559	MRPL27	0.599
			BC073130	NIF3L1	0.308	BG885983	ARHGAP18	0.601
			BC077894	OPRS1	0.308	BC063272	SLC25A22	0.602
			BX853097		0.308	BJ086892		0.603
			BC078529		0.308	BE191747		0.605
			CA983003		0.309	BG264524		0.606
			BC060011	GCKR	0.309	BJ098227	AOAH	0.611
			BC082922	SUCLG1	0.310	BJ074182		0.615
			BC072964	SNX12	0.310			
			BC072040	SLC7A7	0.310			
			BC076848	PKP3	0.311			
			CB560198	MUC2	0.311			
			BC088922		0.312			
			CK800173		0.312			
			BE188971		0.312			
			BX846284	MUC5B	0.313			
			BC068932		0.313			
			BC085199	TMEM62	0.313			
			DR716465	MOGAT2	0.317			
			CK796896		0.319			
			CD302254		0.319			
			BX852437	MFSD2	0.320			
			BC045036	CPT2	0.321			
			BC088685		0.321			
			BQ897453		0.322			
			BC072751		0.322			
			BC043628	CHRND	0.322			
			BQ733291		0.322			
			BJ045456		0.322			
			BC047257	MPP1	0.323			
			U41839	UMOD	0.323			
			BC082954		0.324			
			BC084841	DHFR	0.324			
			BC094074	PCCB	0.324			
			BX855392		0.326			
			BC083004	LONRF1	0.326			
			BC087453		0.326			
			BQ385209	CKB	0.328			
			BC056853	SSR1	0.328			
			BQ386777		0.329			
			BP707684	CAPN2	0.329			
			BP693983	MVK	0.331			
			BC082836	PCBD1	0.331			
			BC073632		0.331			
			BC079717	ENPP4	0.332			
			BC070619	PPA2	0.332			
			AF421880	CACNA1S	0.332			

			CK796828	AKR7A2	0.333			
			CB564765	DNAJB1	0.333			
			BC082939		0.334			
			BC074387	PCSK2	0.334			
			AB024524	ALDOC	0.334			
			BC043819	IRAK2	0.334			
			AW199896		0.335			
			BC073722		0.335			
			CF522107		0.336			
			BC070546	ANKH	0.336			
			AY509892		0.337			
			BC043991	ACO1	0.338			
			BG160257		0.338			
			CD326805		0.338			
			BC076865	GPHN	0.339			
			BC088665	C9orf98	0.339			
			BP691865		0.339			
			BC084799		0.339			
			AB092377		0.339			
			BJ038707		0.339			
			BG020219		0.340			
			BC077335		0.340			
			BF231809		0.340			
			BC070741		0.340			
			BF612654		0.341			
			BC056108	HADHSC	0.343			
			BC059330	PEX11B	0.343			
			AF055588	MGP	0.343			
			BJ618005		0.344			
			BX850790	NIPSNAP1	0.344			
			BC056038	DSCR1	0.344			
			BC042344	TOM1L1	0.345			
			CV523251		0.345			
			BJ639319		0.345			
			BX850131	MCCC2	0.346			
			BC073693	TMEM15	0.346			
			BC071024		0.346			
			BC060383	AKR1B1	0.346			
			BC046843	RFK	0.347			
			AF100165	STK10	0.349			
			BC072808	ARID3A	0.349			
			BC056120	PTER	0.349			
			BC078604		0.349			
			BG810957		0.349			
			CK800243	BZRP	0.349			
			BC072818	SLC41A1	0.350			
			BX855428		0.351			
			BJ031684	HK1	0.352			
			BC081014	GTSE1	0.352			
			CX131603	RSHL2	0.352			
			BF611101	UQCR	0.352			
			BC068809		0.353			
			BC060329	TOB1	0.354			
			BC070973		0.354			
			BC087387	LTB4DH	0.354			
			CA971257		0.355			
			BC087622	PPARA	0.356			
			BC077922	DES	0.357			
			BC097726		0.357			

			BC056847	CYP2C8	0.358			
			BC079791		0.358			
			BC078524		0.358			
			AW200620		0.359			
			BX842761	PRSS15	0.360			
			BC097634		0.360			
			BC076745		0.361			
			BC088702		0.361			
			BC077219	AGXT2L1	0.362			
			BX852056		0.362			
			BQ732878	FETUB	0.362			
			BJ089483		0.363			
			BC081201		0.363			
			BC070852	HOOK2	0.363			
			BC057712	FLJ21106	0.364			
			AW200087	NDUFA1	0.365			
			BC084934	ENTPD4	0.365			
			BC073213	OGDH	0.365			
			CA973487	SPAG1	0.365			
			BX847738		0.365			
			BC044291	DNM1L	0.366			
			BC080033	SUCLA2	0.366			
			BC042262	KCNK1	0.367			
			BC041194	HSD17B12	0.367			
			CD256759		0.368			
			BQ388051	RAB43	0.368			
			BX843871	STYK1	0.368			
			CA974467	STARD10	0.369			
			BP701163		0.369			
			BX850459	C20orf22	0.369			
			U91846	VDR	0.369			
			BC073703		0.369			
			BI446936		0.370			
			BC068891	ACOX2	0.370			
			BC077260	CCNA2	0.370			
			M60680	CDC2	0.370			
			BC092312	KIAA1919	0.372			
			BC044971	SLC7A8	0.372			
			BE192407	HSD17B6	0.372			
			BC081219	DOM3Z	0.373			
			BC087421		0.375			
			BC046378	HYAL2	0.375			
			BE491598		0.375			
			BC068856		0.376			
			BC044053	ZNF622	0.376			
			BC048018	SDCBP	0.378			
			BJ624263		0.378			
			BQ386792		0.378			
			BC077836	UAP1	0.379			
			AW200556	COX6C	0.379			
			BC077618	ACAD9	0.379			
			BE575499		0.379			
			BC077976		0.380			
			BF614590	DNAJA3	0.380			
			AW766099		0.380			
			BC073733	RTCD1	0.380			
			BG409814		0.380			
			BC059994	TOMM70A	0.381			
			BC061648	TSPAN3	0.381			

			BC059996	HSDL2	0.381			
			BC074210	ADH1B	0.383			
			BJ068771		0.383			
			BP719407		0.383			
			BC078029		0.383			
			BC091709		0.384			
			BP685925		0.384			
			BC070762	APITD1	0.384			
			BC087401		0.384			
			BF611511		0.384			
			BC078625	LENG9	0.385			
			BC082712		0.386			
			BX850234	BET1	0.386			
			BC079720	DNAJC5	0.386			
			BC057748	SPATS2	0.387			
			Y09941	CLCN3	0.387			
			BC044274	ESRRA	0.388			
			BC076864		0.388			
			BC073416	DHRS3	0.389			
			BC082410	RNPEP	0.389			
			BC070868	PBP	0.390			
			BC073692	ARL6IP2	0.390			
			CF288050	C6orf85	0.390			
			BQ897327		0.390			
			BX850336		0.392			
			BC045222	HADHB	0.392			
			BC070564		0.392			
			BF611222	COX7A2L	0.393			
			CA972715		0.393			
			BG552903		0.393			
			BF024886		0.393			
			BC041289	CAV3	0.394			
			DR729572		0.394			
			BJ068686		0.395			
			BC075202	SORD	0.395			
			BC060446	SDHA	0.395			
			CK797701		0.396			
			BC041735	ADAMTS13	0.396			
			BJ048218		0.396			
			BC060398	RIOK3	0.397			
			BC075173		0.397			
			BC068748		0.398			
			BC092341		0.399			
			BC045123	CKMT1B	0.399			
			BC087379		0.400			
			AW199690		0.401			
			BC083037	GRHPR	0.401			
			BJ617395		0.401			
			BC072821		0.402			
			BC084392	PHCA	0.402			
			CA972414		0.402			
			CK800585	TIMM8B	0.403			
			X74315	FOXI1	0.403			
			BE026823	NMT1	0.405			
			BC081113	PAK1	0.405			
			M96858	CDC25C	0.405			
			BC068954		0.405			
			BC071122	GATA4	0.405			
			BX846940	TOR1B	0.405			

			BC042221	KCNJ16	0.406			
			BC074325		0.406			
			AW634109	UQCRQ	0.406			
			BC070522	SEC23B	0.406			
			CN323707	ATP5H	0.406			
			AB022787	TYRO3	0.407			
			BC054222	CYP46A1	0.407			
			BG486671		0.407			
			AW634635		0.408			
			BC043859	SDHB	0.408			
			BJ076956	MYL4	0.408			
			CF547931		0.408			
			BC076838	CRY1	0.408			
			BC087364		0.409			
			BC077877	ATF5	0.409			
			BG016864		0.409			
			BC071111	IRF6	0.410			
			BC077963		0.410			
			BP732418		0.410			
			BC054147	MYL7	0.410			
			BC068626		0.410			
			AY534752	CDC14A	0.410			
			BC042931	UQCRC2	0.410			
			BF614639	ATPIF1	0.411			
			BP698942	MRPS23	0.411			
			L13702	GATA5	0.411			
			BC084966	UGCG	0.412			
			CK797287		0.412			
			BC092309	MYOHD1	0.412			
			BC077270	PAFAH1B1	0.413			
			BG016658		0.413			
			BC044981		0.413			
			BC054994	PEPD	0.414			
			BC092017	CYB5B	0.414			
			BJ058051		0.414			
			BX846639	COQ3	0.414			
			BG552014		0.414			
			CK796707	PIGF	0.414			
			BC068899	XPNPEP1	0.415			
			CK797592		0.415			
			BG020414	ABHD14B	0.415			
			CO387976		0.415			
			BC043868	PCYT1A	0.416			
			BF611103		0.416			
			BP741787		0.416			
			BC089190		0.416			
			BC082861		0.416			
			BC071073		0.417			
			BC072330		0.417			
			BC078076	HCCS	0.417			
			BQ388273		0.418			
			CD101250	OCM	0.419			
			BG017853		0.420			
			AW645247		0.420			
			BC084157		0.420			
			BC084325		0.420			
			BE680083		0.420			
			BC042270	GMDS	0.420			
			BC072826		0.420			

			BC092100	TXNDC14	0.421			
			BC060455	CYP2C19	0.421			
			BX852046	GSTA1	0.422			
			BX854775	CKAP2	0.423			
			BC080019		0.423			
			CA971480		0.423			
			AW766394		0.423			
			BC074179	ABAT	0.424			
			BC076770	MGAT1	0.424			
			BC081010	NDE1	0.425			
			BC084267		0.426			
			CV081134	NDUFB9	0.426			
			AW765260	MRPL46	0.426			
			BC078569	NDUFS8	0.426			
			BC073197		0.427			
			BC097801	CIAPIN1	0.427			
			BC068670		0.427			
			AW764580	KRT8	0.427			
			BC087330		0.427			
			BX849263	COX5B	0.427			
			BC073643	UROD	0.428			
			BE509325		0.428			
			AB046536	MTCH2	0.429			
			AW164955		0.429			
			BC074139	GMPR2	0.429			
			BC094201	NPEPL1	0.430			
			BC073496		0.430			
			BM192882		0.430			
			BP703629		0.430			
			BC081159		0.431			
			CK804222	HSD17B6	0.431			
			AW767623	CRISP3	0.432			
			BC043999	SPR	0.432			
			BC072124	ARVCF	0.432			
			BC073021	CCDC58	0.433			
			BC087380		0.433			
			BC075135		0.433			
			CD301859		0.434			
			BC073490	ALDH1L1	0.435			
			M59454	YBX2	0.436			
			BC087381		0.437			
			AB176537	LRRC54	0.437			
			BC068661		0.437			
			BC079714	NDFIP2	0.437			
			BE131864		0.437			
			CA972632		0.437			
			CF289962		0.437			
			BF072406	NDUFA3	0.438			
			BC087501		0.438			
			BX844087		0.438			
			BC077371	FLRT3	0.438			
			BC072175	SDFR2	0.441			
			D49837	GUCY2C	0.441			
			CK799329		0.441			
			BC045259	HSPA9B	0.441			
			BC078536		0.441			
			BX854511		0.442			
			BC092390		0.443			
			BC084928	KIF23	0.444			

			BC056131	TRIM16	0.444			
			BG438810		0.444			
			BC044037	DNAJC3	0.444			
			BC041231	TEX261	0.444			
			AW200613		0.445			
			BX850733		0.445			
			BC080059	GMPPB	0.445			
			BC077199	SDHD	0.446			
			BC070627	AP1M2	0.446			
			BC079712		0.447			
			BJ633615	PLDN	0.447			
			CF271280		0.447			
			BX849277		0.447			
			BC084277		0.447			
			CA981435	FNBP1L	0.447			
			CK799837		0.448			
			BC073239	CHCHD2	0.448			
			BC046947	CASQ1	0.448			
			BC080102	TMEM93	0.448			
			BC073059		0.448			
			BC088716		0.448			
			AY114144	TNNT3	0.449			
			BC079804	PNPLA2	0.449			
			BQ735326		0.449			
			BC080116	C1QTNF4	0.450			
			BF613812	CASC3	0.450			
			BC082901		0.450			
			BC082919	NUCB1	0.451			
			BC071154	TAX1BP3	0.451			
			BC068878		0.452			
			CA974265		0.452			
			BE680570		0.452			
			BC072314	FLAD1	0.452			
			CO385090		0.453			
			BC083009	C18orf55	0.454			
			BC073377		0.454			
			CA982851	NDUFB8	0.454			
			BC056136	TXNRD2	0.454			
			BC077419	MYH4	0.454			
			BC070789		0.455			
			BC073574	SCCPDH	0.455			
			BC077578	AP1M2	0.455			
			BC082698		0.455			
			BJ613168	EIF2AK2	0.455			
			BC095924	MGST3	0.455			
			BC050257	TOMM40	0.456			
			U10161	HTR7	0.456			
			BG163154		0.456			
			BX852379	C18orf55	0.456			
			AJ304990	CCNB3	0.456			
			BC056024	TCF2	0.457			
			CB565128		0.457			
			BC085049	MRPL9	0.457			
			BX844434		0.457			
			CK797281		0.458			
			CK798516	STAMBP	0.459			
			AW645440		0.459			
			BC049394	NDUFS1	0.459			
			BP727769		0.460			

			CK805158	PMVK	0.460			
			BJ060494		0.461			
			BQ735737		0.461			
			CV075669		0.461			
			BC054204	METAP1	0.461			
			BC084958		0.462			
			BJ635358		0.462			
			BC072731	NDRG3	0.462			
			BC074341		0.462			
			CX131946		0.462			
			BC060470	SAMM50	0.463			
			AW641055	PCTK2	0.463			
			BC088690	C18orf19	0.463			
			BC056042	TJP2	0.464			
			BC045127	CYC1	0.465			
			U35728	TFAM	0.465			
			BF613503		0.465			
			BG811521		0.467			
			AF308152	P2RX4	0.467			
			AW460440		0.467			
			BC076752	GLO1	0.467			
			AW200424	COX7C	0.467			
			AY271302		0.467			
			BC078575	NIT1	0.467			
			BC084931	KCNK5	0.467			
			BC076719		0.468			
			CD362829		0.468			
			M80257	YBX2	0.468			
			CD101209		0.468			
			BC087343	PSMD10	0.469			
			BG578422		0.469			
			BC073217	NDUFB7	0.469			
			BC073719		0.470			
			BE491189	MRPL41	0.470			
			BC087436		0.470			
			BC087349		0.471			
			CF288031		0.471			
			CK797961		0.471			
			BC074134	CYB561	0.472			
			BC084256	ATP6AP1	0.472			
			AF338157	IRX4	0.472			
			BG514494		0.472			
			BC046656	RHOA	0.473			
			BG234364		0.473			
			BC077194	GCDH	0.474			
			BC087397	STK19	0.474			
			BG023328		0.474			
			BX850255	TPRKB	0.474			
			BC072225	BCAR3	0.475			
			CB756825	NDUFS7	0.475			
			AF027728	CENPE	0.476			
			BC054282	LUM	0.476			
			BC060749	LPGAT1	0.476			
			BC078619		0.476			
			BC082388	C20orf112	0.476			
			BC085059	TMEM41B	0.476			
			BQ398256		0.477			
			CB562834	VIL1	0.477			
			AW199573	BRP44	0.478			

			BC068714	MAD2L1	0.478			
			BG884808		0.478			
			BC059316	NADK	0.479			
			BG264524		0.480			
			CD254188		0.480			
			CO388496		0.480			
			BE506967	NDUFA6	0.481			
			BC082626		0.481			
			BE576620		0.481			
			CF522635	CLPB	0.481			
			BC056096	XRCC6BP1	0.481			
			BC081246	LRRC22	0.481			
			BC077639		0.481			
			BC091638	AP2A2	0.481			
			AJ557446	KPNA2	0.483			
			BE575428		0.483			
			BM181057	FGA	0.483			
			BC070561		0.484			
			BC056098	NDUFS5	0.484			
			BC092028	PPP1R3B	0.484			
			BC070821		0.484			
			CD326370	BCORL1	0.484			
			BC042294	SLC3A2	0.485			
			DQ096919		0.486			
			BC073388		0.486			
			BC070870	LRPPRC	0.486			
			BG513473		0.486			
			BG811799	DCI	0.487			
			BJ616363		0.487			
			BQ735035		0.487			
			BJ094248		0.487			
			AJ575267	ADH5	0.488			
			BX853488		0.488			
			CF520187		0.488			
			CK797494		0.489			
			BC081090	TIMM44	0.489			
			BC041187	NR1I2	0.489			
			BC080129	GLO1	0.489			
			AB093561	IQGAP1	0.489			
			BC046683	RAB6A	0.490			
			BP703418		0.490			
			BX842712	ZNF179	0.490			
			CA981670		0.490			
			CK799932	GRPEL1	0.490			
			M55163	FGFR1	0.490			
			BC072961	PRKAB1	0.491			
			BC046577	VDAC1	0.492			
			BC054248	FTCD	0.492			
			BC080009		0.493			
			BG438927	RPL13A	0.493			
			BC070602	MFN2	0.493			
			BX847608	MRPL19	0.493			
			BC054292	TXNDC9	0.494			
			BX849973		0.494			
			BC094149		0.494			
			BJ624120		0.494			
			CK798956		0.494			
			BC073699		0.495			
			BJ613998	ATP5G3	0.496			

			BP704928		0.496			
			X56451	SRF	0.496			
			BC073043	PBP	0.496			
			AW634424	ATP5J	0.496			
			BC042288	CDC20	0.497			
			CB558400		0.498			
			AW646316		0.498			
			BC060355	GAS6	0.498			
			BC077484	PGAM1	0.498			
			BC054152	SQRDL	0.499			
			BC074447	CCDC19	0.499			
			BG017560		0.499			
			AW200552		0.499			
			BC086270		0.499			
			BJ094084		0.499			
			BQ884366		0.499			
			BU152749		0.499			
			BX848034		0.499			
			AW200217	MRPL37	0.500			
			BJ644027		0.500			
			BJ078276	WDR39	0.500			
			CB561882	TBC1D14	0.500			
			BC042224	HNF4A	0.501			
			BX848092		0.501			
			BC070630	PGAM1	0.501			
			CF286131		0.501			
			CK798748	C6orf125	0.501			
			BC088820	NDUFS7	0.502			
			BC080113	C14orf100	0.502			
			CB565751	PYCR2	0.502			
			CD099938		0.502			
			BF072340		0.503			
			BP684778		0.503			
			CB943246		0.503			
			BX853482	CD3EAP	0.503			
			BG812314		0.504			
			BC070749	SSX2IP	0.504			
			AF525882	TERF1	0.504			
			BX850181	C3orf59	0.505			
			CD255149		0.505			
			CD325445		0.505			
			BC054234	GCG	0.505			
			BC084922	KIF20A	0.505			
			BI095472		0.505			
			BQ725085		0.505			
			BC070635	ARL5A	0.505			
			BC083022	C20orf155	0.506			
			CV076731		0.506			
			BE507594		0.506			
			BC074293	CCNG2	0.506			
			BC076747	POU3F4	0.506			
			BC082882	C20orf45	0.506			
			BC073389		0.506			
			BG162399		0.506			
			CK797441		0.506			
			BC087292		0.507			
			CB565452		0.507			
			CO388111		0.507			
			BG515438	SDHC	0.507			

			BQ884389	EIF4G3	0.507			
			CB756236		0.507			
			BC082429	DMD	0.508			
			DQ096958		0.508			
			BC077936	RIOK3	0.509			
			CD301585	HMG2L1	0.509			
			BJ089988		0.509			
			BC072042	DNAJB2	0.509			
			CK806198		0.509			
			BC076746		0.510			
			BJ631189	TM2D2	0.510			
			BQ900459	BASP1	0.510			
			CK800619		0.510			
			BC045028	SCARB2	0.510			
			BC078065		0.510			
			BC082908	MYOZ2	0.510			
			BC060485	PKM2	0.511			
			BC081147		0.511			
			CB943372		0.511			
			BJ068590		0.512			
			BC045019	MTHFD1	0.512			
			BC081192	MRPL2	0.512			
			BC082391		0.512			
			CF286172	PAFAH1B1	0.512			
			BC073458		0.513			
			BC073581	PTDSR	0.513			
			BC088936	PBK	0.513			
			BC043896	ADSS	0.514			
			CX132068		0.514			
			BX852353		0.514			
			BC085047		0.514			
			BC070726	SLC39A9	0.515			
			BC077536	MRPL15	0.515			
			BP715593		0.515			
			BG020766	COL18A1	0.515			
			BC077405	DTWD1	0.516			
			BC084366	NDUFA10	0.516			
			BQ723944		0.516			
			CF520694		0.516			
			BC044052	AP1G1	0.517			
			CD253356		0.517			
			BG021054	SLC38A6	0.518			
			BC043879	ZNF622	0.519			
			BC053821	FBXL15	0.519			
			BC074256		0.519			
			BJ028936	TMEM69	0.519			
			BQ736392		0.519			
			BC058770	RBM5	0.519			
			AW164964		0.520			
			BG021649	CLPB	0.520			
			BC082885		0.520			
			BC084430	NUDT12	0.520			
			BQ737312		0.521			
			CB561733		0.521			
			BJ643315	CRBN	0.521			
			CA980878	ZC3H11A	0.521			
			BC084983	PPP1R15B	0.522			
			BQ884377	EIF2S2	0.523			
			BC094210		0.523			

			BC084851		0.523				
			AW200171	ATP5L	0.524				
			BC077943	PI4K2B	0.524				
			BX847553	C20orf108	0.524				
			BJ091734		0.524				
			CK804869		0.524				
			BC054146	CDC2	0.525				
			BC078095	MMS19L	0.525				
			BC089120	DSC2	0.525				
			CB565870		0.525				
			BC043754	AARSD1	0.525				
			BJ094105	AOX1	0.526				
			BX854027	LBR	0.526				
			BC043633	TBL2	0.527				
			BC077838		0.527				
			BM191937		0.527				
			BI449890	NDUFA2	0.527				
			BC078591		0.527				
			BC074288	BCAP31	0.527				
			BC077224	KIF20A	0.528				
			CA973724		0.528				
			AW632936		0.529				
			BC073110	PTPN3	0.529				
			BC074361	RNF185	0.529				
			BG022668		0.529				
			CA971580		0.529				
			BC070597	SPBC24	0.529				
			BC074219	IDH3G	0.529				
			BC077236	GYG1	0.529				
			BQ734970	PDCD8	0.530				
			CA981840		0.530				
			BC059787	ETFB	0.530				
			BC042304	ELOVL1	0.530				
			BC074193		0.530				
			AY644401		0.531				
			BJ618672	PEX12	0.531				
			AW147428	NDUFS6	0.531				
			BC071043	SEC31L2	0.531				
			BG811942		0.531				
			BC074122		0.531				
			BJ642858	PTDSR	0.531				
			AW199813	NDUFB11	0.532				
			BC073591		0.532				
			BC073433	MAL2	0.532				
			BC074314	SCCPDH	0.532				
			BC088708	NUBP1	0.532				
			BX843474		0.532				
			BC043765		0.533				
			BG407870		0.533				
			BC046842	MYO1E	0.533				
			BC045130	HSPA9B	0.534				
			BG160139	HBE1	0.534				
			BQ897365	TBL3	0.534				
			AW147250		0.534				
			CK798527		0.534				
			BC088701		0.534				
			CB941448		0.534				
			AW200445	NDUFC2	0.535				
			BC089273	MTHFS	0.535				

			BI447707		0.535			
			BQ733221		0.535			
			BC045232	WDR23	0.535			
			BP741589		0.536			
			CK798929	C10orf57	0.536			
			BC059963	BACE2	0.536			
			BC084949	TIMM22	0.536			
			BX845543		0.536			
			AW641159	BAX	0.537			
			BC084860	ME3	0.537			
			CA980798	EIF3S2	0.537			
			CF284199		0.537			
			CO382612		0.538			
			BF047364	ATP5J2	0.538			
			BC084265	HMBS	0.539			
			AW782692		0.539			
			CK799019	TIMM13	0.539			
			BC068912		0.539			
			BC072308	SLC25A11	0.539			
			BC077901	FHL1	0.539			
			BC074463	FCN2	0.541			
			BJ090813		0.541			
			AW641831		0.541			
			AW764652		0.541			
			BC074474	ALAD	0.541			
			BC084776	ACLY	0.541			
			BP695826		0.542			
			CD099041		0.543			
			CD256980	CRYBA1	0.543			
			BC084789	ZRANB1	0.543			
			BG885214		0.543			
			BC056855	FABP7	0.544			
			BC079921	PKM2	0.544			
			BC046849	SLC25A3	0.545			
			BF615644		0.545			
			BC074442		0.546			
			BC087534		0.546			
			BX849722	C10orf35	0.546			
			CK800377	RER1	0.546			
			BC046372	JMJD2B	0.547			
			AW646338		0.547			
			BJ032297	DDEFL1	0.547			
			CA972911		0.548			
			BC056845	ACAD8	0.548			
			CB564209		0.548			
			AF064810	FOXB1	0.549			
			BC078126		0.549			
			BC081053	TXNRD1	0.549			
			BJ642503	HTATIP2	0.549			
			J03167	CCNB2	0.550			
			AW200559	MRPL27	0.550			
			BC073069	PEX3	0.550			
			BC073691	TMEM38B	0.551			
			BC077396	TPX2	0.551			
			BC073466		0.551			
			BC077791		0.551			
			BF025307	MRPL30	0.551			
			U88065	ADAR	0.551			
			BC073000		0.552			

			BC082405		0.552			
			BE505878	DNAJB1	0.552			
			BG812312		0.552			
			BC075233	EBAG9	0.552			
			CD301806	PRKCA	0.552			
			X82012	KIF4A	0.552			
			CK800731		0.552			
			BC060755	DDR1	0.552			
			AW645308	POLR2K	0.553			
			BX853528		0.553			
			AF430841	TUBE1	0.553			
			BG018014	FA2H	0.553			
			BC085046		0.553			
			BC070655	HIGD1A	0.554			
			BC054203	DHCR7	0.554			
			BC082352	SEC24C	0.554			
			BG579281	ANK1	0.554			
			BC072860	CHCHD3	0.554			
			BQ732150	CLTC	0.554			
			BC056000	PRSS27	0.555			
			CK797414		0.555			
			BC077739	HMMR	0.555			
			BJ069360	ADHFE1	0.556			
			CB562051	MUC2	0.556			
			CB564148	PSMA1	0.556			
			BC073330		0.556			
			BC077831	PITPNB	0.557			
			BE132199	ARID1A	0.557			
			BQ731671		0.557			
			CF287253		0.557			
			BG552585	FIP1L1	0.557			
			BC080022	HGD	0.558			
			BC081161	DHRS10	0.558			
			BJ048181	USP13	0.558			
			BC076792		0.559			
			CK796680	FNTA	0.559			
			BC072857		0.559			
			BJ035521		0.559			
			CA982088	MRPS9	0.560			
			AW199660	IFNGR2	0.561			
			CD360486		0.561			
			BX853537	KIAA0319L	0.561			
			BG438757		0.562			
			CN327872		0.562			
			BC071119	C20orf24	0.562			
			BC074233	RAB18	0.562			
			BX845533		0.563			
			CK804953	MRPS22	0.563			
			BC084371	KBTBD10	0.563			
			BC041285	LRRC59	0.564			
			CD255843	PIR	0.564			
			CD361292		0.564			
			CX130594		0.564			
			CV111152		0.564			
			BJ067204		0.565			
			BQ385861	METTL4	0.565			
			BC084816	RNUXA	0.565			
			CD101556		0.565			
			CD253818		0.565			

			CO384340		0.565			
			CX132118	DTX3	0.565			
			BF047624	FNDC3A	0.566			
			BX850233		0.566			
			BC054289	IFITM2	0.566			
			CK800116		0.566			
			CK796739		0.567			
			CK797282	ATP5O	0.567			
			X85096	EEF1D	0.567			
			BC088796		0.567			
			BG017514	NDUFB2	0.567			
			BG022890		0.567			
			CX131298		0.567			
			BC072247	HMGCL	0.568			
			BG731777		0.568			
			BC073058	SMYD5	0.568			
			BX843145		0.568			
			CF549152	GRK5	0.568			
			BJ100432		0.569			
			CD326004		0.569			
			AW633016		0.569			
			BC083043		0.569			
			CB756739	PHYHIPL	0.569			
			BC091637		0.569			
			BX849030	CRBN	0.569			
			BC068822	SURF4	0.570			
			BJ092798		0.570			
			BC079729		0.570			
			BJ051767		0.570			
			BC054310	RAB3IP	0.570			
			BG810931		0.570			
			CA983383		0.570			
			BX851706		0.571			
			BC045016	DLST	0.571			
			CK740430		0.571			
			BC070771		0.572			
			BC068968	MASTL	0.572			
			BC099015	IBRDC1	0.572			
			CB942817		0.572			
			BC068928	SLC33A1	0.572			
			BC077516	RAB3IP	0.572			
			AF465788		0.573			
			CB756146	PLEKHF1	0.573			
			BC082484	ZMPSTE24	0.573			
			BE490862	NDUFB10	0.573			
			BG345959		0.573			
			BU152767	COPE	0.573			
			CF548021		0.573			
			BC077768	CBWD1	0.574			
			CD328019	MRPL4	0.574			
			CK797275		0.574			
			BF048527		0.574			
			BG020035		0.574			
			AW200200	ACSS2	0.574			
			CF546987		0.575			
			BC044976	KIF2C	0.575			
			BG018651		0.575			
			BF427978		0.576			
			BX849650		0.577			

			CA983623		0.577			
			BF231622		0.578			
			BC044007	PKM2	0.578			
			BC071009	GALNT4	0.578			
			CK800385	NDUFB9	0.579			
			BC043793	AP2B1	0.579			
			CA973725	RAB15	0.579			
			CK800512		0.579			
			BG161935		0.579			
			BC087450		0.580			
			BX855559		0.580			
			CD327733		0.580			
			BC077311	UQCRC2	0.580			
			BP708884		0.580			
			BI446779		0.581			
			BC090198		0.582			
			BC060421	UCK1	0.583			
			BJ043529		0.585			
			DQ096963	NDUFAB1	0.585			
			BQ398490		0.587			
			BJ073868	PDCD6	0.587			
			BX854807		0.587			
			BC082397	ETFDH	0.588			
			BC075229		0.588			
			BJ638356		0.588			
			BU152748	TNS1	0.589			
			BC077310	C20orf24	0.589			
			BC061655	PLS3	0.589			
			BJ618670	NGEF	0.589			
			BC057708	MVP	0.590			
			BQ733181		0.590			
			BI443191		0.591			
			BC043826	FAM3A	0.596			
			CD301916		0.596			
			AB021705	MAP4	0.597			
			BC084826	GMFB	0.597			
			BJ074182		0.599			
			BE508799	ANXA7	0.599			
			BJ629687		0.600			
			BE679121		0.600			
			CD253472		0.603			
			BC070643	SPTLC1	0.606			
			CD253362		0.606			
			CB564398		0.607			
			CB592369	RPL36	0.610			
			CB565187		0.611			

Supplemental Table S4: Genes validated by RT-qPCR and their associated PCA cluster

	GenBank	Cluster	Gene Name
1	AF351126	1	Enhancer of zeste
2	BC044030	1	Tubulin beta-2 chain
3	BC070743	1	REV1-like isoform 2
4	BC073348	1	regulator of G-protein signalling 18
5	BC074249	1	transmembrane 4 superfamily member 9
6	BC075151	1	annexin A1
7	BC076737	1	cyclin J
8	BC076743	1	Annexin A6
9	BC077649	1	Keratin 16
10	BC077905	1	ATPase, Ca++ transportin
11	BC081076	1	solute carrier family 2
12	BC085060	1	*Transcribed locus*
13	BX851035	1	*Transcribed locus*
14	BX851861	1	*Transcribed locus*
15	L29495	1	Pim-1 oncogene
16	L39213	1	Morphogen
17	M18350	1	68 kDa serum albumin
18	M35362	1	X.laevis thyroid hormone receptor beta B2
19	U08407	1	Arginase
20	U37375	1	Leucine zipper gene 8
21	U37376	1	MAM domain protein
22	U41824	1	Collagenase-3
23	U41855	1	Gene 12-1b mRNA
24	Z27093	1	Stromelysin-3
25	AB046536	2	Mitochondrial carrier homolog 2
26	AB086829	2	Adult keratin XAK-C
27	AF061833	2	Aldehyde dehydrogenase class I
28	BC043731	2	UDP-glucose dehydrogenase
29	BC060011	2	Glucokinase regulator
30	BC073341	2	dehydrogenase/reductase
31	BC084269	2	threonine synthase-like 2
32	BC084618	2	Cytochrome P450 XL-304
33	BC084752	2	short-chain dehydrogenase/reductase
34	BC084855	2	arylacetamide deacetylase-like 4
35	BC084936	2	Adaptor protein BLNK
36	BC086290	2	Neurula-specific ferredoxin reductase-like protein
37	BX848224	2	ribosomal protein S8
38	BX853035	2	*Transcribed locus*
39	U76636	2	Calbindin D28k
40	X93494	2	Glucokinase
41	Z49827	2	Hepatocyte nuclear factor 4 beta (HNF4 beta)
42	AB003358	3	V-yes-1 Yamaguchi sarcoma viral related oncogene
43	AY164461	3	Secreted frizzled-related protein 5
44	BC041213	3	carbonic anhydrase II
45	BC043624	3	Phosphorylase phosphatase
46	BC043819	3	interleukin-1 receptor-associated kinase 2
47	BC043879	3	zinc finger-like protein 9
48	BC044007	3	Pyruvate kinase, muscle

49	BC046378	3	Lysosomal hyaluronidase (Hyal2)
50	BC047247	3	Potassium channel, subfamily K, member 6
51	BC056128	3	C. elegans WNT family member precursor (40.4 kD)
52	BC056840	3	Serotransferrin B
53	BC072842	3	tripartite motif-containing 2
54	BC077065	3	melanoregulin
55	BC090160	3	*Transcribed locus*
56	BJ057663	3	cytochrome c oxidase
57	BX844453	3	Deiodinase, iodothyronine, type I
58	BC045083	4	Elongation factor 1 alpha, oocyte form
59	BC054202	4	*Transcribed locus*
60	BC060751	4	L amino acid transporter-1 LAT-1
61	BC073316	4	G-protein-coupled receptor induced protein
62	BC078061	4	Elastase 3B, pancreatic
63	BC081152	4	Xenopus NFI-X2 transcription factor
64	BC081272	4	calbindin 1
65	BC082684	4	*Transcribed locus*
66	BC084848	4	transgelin
67	BQ735436	4	*Transcribed locus*
68	BX845609	4	trypsin 10
69	CB559907	4	*Transcribed locus*
70	D13688	4	MHC class II antigen isoform 1
71	L28111	4	Deiodinase, iodothyronine, type III
72	X90838	4	Sodium-calcium exchanger
73	AB022088	5	Cytochrome P450
74	AF358869	5	Wee1-like kinase
75	BC054153	5	Glutamate-ammonia ligase
76	BC070314	5	Matrix Gla protein
77	BC077417	5	Kallikrein B
78	AF062387	6	TAP2 protein
79	AY204551	6	Transporter associated with antigen processing 1
80	AY204554	6	Transporter associated with antigen processing 2
81	BC044008	6	Similar to macrophage stimulating 1
82	BC060360	6	pancreatic lipase-related protein 2
83	BC073555	6	chymotrypsin-like
84	BC077896	6	Interleukin 10 receptor, beta

Supplemental Table S5: Regulated genes in different PCA clusters

GenBank	HUGO	Cluster
AB001073	ACVR1	1
AB006906	VLDLR	1
AB014611	FOXD3	1
AB015205	CNTN1	1
AB025112	NPR2	1
AB026192		1
AB037688	PTCH2	1
AB042255	CRY1	1
AB049354	FSTL1	1
AB052692	SOX17	1
AB060971		1
AB061521	CAPN1	1
AB072005	SYNGR1	1
AB083001	CAMKK2	1
AB085173	HRMT1L2	1
AB085631	ANKRD28	1
AB086020	SOX4	1
AB087137	ROR2	1
AB091339	FCN1	1
AB091342	FCN1	1
AB093560	IQGAP1	1
AB096099	EIF4G2	1
AB111446		1
AB117614	FGFRL1	1
AB191720	TSC22D1	1
AF001596	HOXC8	1
AF002983	CDH11	1
AF032382	ADAM9	1
AF032383	ADAM22	1
AF040993		1
AF043643	PCDH7	1
AF044080	NCOA3	1
AF051784	SMC1L1	1
AF053935	NOS1	1
AF055980	IGF1R	1
AF059570	SFRP2	1
AF072455	MMP2	1
AF081803	HCK	1
AF107662	ETV1	1
AF109923	GLI2	1
AF131890	NGFR	1
AF139165	FZD2	1
AF154112	SIN3A	1
AF157558	NR2F1	1
AF170337		1
AF170339	C18orf26	1

AF172399	NGFR	1
AF187864	MARCKSL1	1
AF197944	PTPRD	1
AF212298	SEPT2	1
AF212299	TERT	1
AF224746	BICC1	1
AF232672	FKBP10	1
AF244359	RGS19	1
AF283562	LEFTY1	1
AF286645	HAND2	1
AF287147	LEF1	1
AF302423	DDX21	1
AF302765	PTCH	1
AF302766	SMO	1
AF310007	SALL1	1
AF310008	YRDC	1
AF317656	AKT1	1
AF321229	INVS	1
AF346838	TRPS1	1
AF351126	EZH2	1
AF368043	SNAI2	1
AF374473	LMO2	1
AF387815	PRICKLE1	1
AF388035	CDON	1
AF393242	TLL1	1
AF401352	SH2D1A	1
AF411389	BIVM	1
AF461119	ROBO1	1
AF466017	ARMC1	1
AF482757	GTF2IRD1	1
AF499689	FCER1G	1
AF545659	FLOT1	1
AF546707	TUBD1	1
AF549892		1
AF549913		1
AJ001754	FZD3	1
AJ009284	HES6	1
AJ010497	IL1B	1
AJ242680	FOXF1	1
AJ278067		1
AJ311059	SMAD3	1
AJ311602	KIF3A	1
AJ311840	NOL5A	1
AJ345114	GRM1	1
AJ507633	SLC35A3	1
AJ549811	HTR2B	1
AJ605777		1
AJ635202	GPR68	1
AW148044		1

AW148133	C22orf3	1
AW148246	C21orf33	1
AW199542	ARL3	1
AW199681	ME2	1
AW199724		1
AW199751	ENY2	1
AW199824		1
AW199861		1
AW199868		1
AW199874		1
AW200038	RFWD3	1
AW200112		1
AW200140	BTN3A1	1
AW200150		1
AW200252		1
AW200266	SLAMF8	1
AW200275		1
AW200278	FTH1	1
AW200477		1
AW200487		1
AW200562	LOC147991	1
AW200608		1
AW460799		1
AW633477		1
AW635234		1
AW636221		1
AW636919		1
AW637943		1
AW638861		1
AW639424		1
AW639557		1
AW640053	PIN4	1
AW640119	TPK1	1
AW640423		1
AW640455		1
AW640576		1
AW640794		1
AW642902	EIF2C4	1
AW643154	ALLC	1
AW643311		1
AW644128		1
AW645391		1
AW645397		1
AW645526	MDM1	1
AW646020	LRCH2	1
AW646642		1
AW765812		1
AW766084		1
AW766222	PDCD6	1

AW766880		1
AW766889		1
AW766900		1
AW767839		1
AW768051		1
AY008299	ILF3	1
AY009369	ARL4A	1
AY030051	SEMA3A	1
AY043258	PPP2R3A	1
AY046070	SLIT2	1
AY052629	IGFBP5	1
AY057997	GJB3	1
AY069942	MPO	1
AY182251	RTN1	1
AY216793	ENC1	1
AY260733	PPP1R1A	1
AY262358	CD99	1
AY289193	RNPC1	1
AY310397		1
AY312062	PTPN12	1
AY318877	HUNK	1
AY318878	HUNK	1
AY344472	LDLRAP1	1
AY344472	LDLRAP1	1
AY363162	TECTA	1
AY458020	TNFRSF12A	1
AY491055	EDIL3	1
AY495962		1
AY566230	NIN	1
AY573848	CDKN1A	1
AY616034	MYO10	1
AY644453	TREX1	1
AY660871		1
AY686699	EVL	1
AY714077		1
AY762376	SMARCA2	1
AY885243		1
BC041207	MARCKS	1
BC041233	CAPZB	1
BC041248	FKBP1A	1
BC041265	TUBB6	1
BC041726	PACSIN3	1
BC041727	PMP22	1
BC041731	HOXA3	1
BC041733	SH2D3C	1
BC041758	H1FX	1
BC042235	SLC37A2	1
BC042263	SHOC2	1
BC042267	CEPT1	1

BC042274	GNS	1
BC042290	H3F3A	1
BC042309	H3F3A	1
BC042315	IMPDH2	1
BC042348	FAM49B	1
BC042351	GRASP	1
BC043635	ARG1	1
BC043735	ELAVL4	1
BC043741	FKBP9	1
BC043747	MYCN	1
BC043749	FYN	1
BC043751	RPE65	1
BC043762	RASSF6	1
BC043774	GTF2E1	1
BC043779	SLC16A1	1
BC043808	CNN3	1
BC043825	PSMD6	1
BC043838		1
BC043887	PRPSAP1	1
BC043893	PLOD3	1
BC043948	PDGFRA	1
BC043961	CNTFR	1
BC043963	CLK2	1
BC043968	TSPAN31	1
BC043981	ILF2	1
BC044009	HMGB3	1
BC044016	TGIF	1
BC044024	MEIS2	1
BC044027	SFXN1	1
BC044030	TUBB2B	1
BC044038	UBE2H	1
BC044039	ID3	1
BC044076		1
BC044096	SS18	1
BC044107	GCLM	1
BC044117	PELI1	1
BC044121	DOK4	1
BC044122	IMPDH2	1
BC044123	GNAI3	1
BC044261	PRDM1	1
BC044267	EGFL7	1
BC044269	CALCRL	1
BC044270	GJA7	1
BC044315	LYPLA2	1
BC044316	EDNRA	1
BC044323	RASGRP2	1
BC044329	SERPINH1	1
BC044330	SLC16A3	1
BC044671	MAPRE1	1

BC044672	PTK9L	1
BC044678	AKR1C3	1
BC044686	PCMTD2	1
BC044687	SFRP2	1
BC044697	TSPAN7	1
BC044956	RNPC1	1
BC044963	CXCR4	1
BC044977	GALK2	1
BC045011	PARVB	1
BC045017	MLSTD2	1
BC045027	TES	1
BC045035	CUGBP2	1
BC045043	ARPC1B	1
BC045044	DSTN	1
BC045050	MYO5A	1
BC045059	ARL5B	1
BC045092	SSBP3	1
BC045118	PCDHGA12	1
BC045120	GAL3ST2	1
BC045134	SRC	1
BC045209	PRPH	1
BC045226	CRLF3	1
BC045234	SLC22A15	1
BC045244	COL9A1	1
BC045253		1
BC045258	CTNNB1	1
BC045260	HNRPA1	1
BC045262	HOMER3	1
BC045271	SUMO2	1
BC046253	INDO	1
BC046257	CNN2	1
BC046259	HAPLN3	1
BC046572	ZNF238	1
BC046657	PTAFR	1
BC046658	MIDN	1
BC046667	CTSB	1
BC046668	SFRS6	1
BC046669	SLC38A6	1
BC046671	SPIB	1
BC046678	CLU	1
BC046679	SFRS1	1
BC046703	EMP3	1
BC046716	ATP1B3	1
BC046719	PFN2	1
BC046731	NOVA1	1
BC046742	NT5DC2	1
BC046852	BIN1	1
BC046954	DNAJA2	1
BC047245	PYGB	1

BC047249	GNG7	1
BC047254	CUEDC1	1
BC047258	GCAT	1
BC048020	LMO4	1
BC048222	THBS3	1
BC048771	CLDN7	1
BC049004	TUBB	1
BC049389	EDG2	1
BC053786	ANXA1	1
BC053814	INMT	1
BC054150	SPARC	1
BC054155		1
BC054158	STMN4	1
BC054161	VIM	1
BC054166	ID3	1
BC054185	FGG	1
BC054225	HSPB8	1
BC054257	LDHB	1
BC054258	ATP6V0C	1
BC054285	CDK2AP1	1
BC054306	MOSPD1	1
BC054947	MMP2	1
BC054953	PCOLCE	1
BC054962	CBX5	1
BC054981	TEF	1
BC055959	BMP7	1
BC055972	TFPI2	1
BC055991	EMID1	1
BC055994	CASC4	1
BC055998	AGTRL1	1
BC056007	HM13	1
BC056020	PRPH	1
BC056023	FLT1	1
BC056040	MMP13	1
BC056041	PLA2G4A	1
BC056051	ELK3	1
BC056055	LCP1	1
BC056059	CTSS	1
BC056076	CDC2L6	1
BC056079	ARHGDIB	1
BC056080	MMP13	1
BC056081	MXRA8	1
BC056085	RGS19	1
BC056109	CTSC	1
BC056112	CHN1	1
BC056117	CHST11	1
BC056126	SH3BGRL2	1
BC056842	LGMN	1
BC056857	SNAI2	1

BC057696	CTDSP2	1
BC057714	RGS6	1
BC057719	FKBP9	1
BC057721	INA	1
BC057731	SEPHS1	1
BC057732	SEPT9	1
BC057739	RUNX1	1
BC057742	ZYG11BL	1
BC057746	SMAD1	1
BC058326	SDS	1
BC059285	TBX2	1
BC059286	SMAD7	1
BC059290	OPHN1	1
BC059292	LRRN1	1
BC059296	SOX4	1
BC059297	PPT2	1
BC059299	CPLX2	1
BC059300	RHOG	1
BC059319	ITGB2	1
BC059320	HHEX	1
BC059324	G6PD	1
BC059329		1
BC059789	MAL	1
BC059973	C16orf35	1
BC059981	PLD3	1
BC059982	DPYSL3	1
BC059983	SIAE	1
BC059995	CPN1	1
BC059999	DAB2	1
BC060027	DAB2	1
BC060327	EAF2	1
BC060330	F10	1
BC060353	CFH	1
BC060396	ELMO2	1
BC060427	FMO2	1
BC060430	PPAT	1
BC060434	CTTN	1
BC060435	HMOX1	1
BC060458	POLR2B	1
BC060476	C9orf7	1
BC060480	GPRC5A	1
BC060494	SP4	1
BC060754	CORO1A	1
BC061651	MGRN1	1
BC061653	ACY1	1
BC061654	PLAT	1
BC061657	TNFAIP8L3	1
BC061674	GPR146	1
BC061685	CTSD	1

BC061934	STOX2	1
BC061941	CCDC63	1
BC061947	KIT	1
BC063273	INPP5D	1
BC063274	GPR124	1
BC063732	FAM79B	1
BC063737	MLSTD2	1
BC063738		1
BC066119	C7orf19	1
BC068634	TWSG1	1
BC068642	SMAD6	1
BC068655		1
BC068658	ZNF423	1
BC068659	REEP5	1
BC068688		1
BC068723	TRIM36	1
BC068726	IER5	1
BC068733	HS2ST1	1
BC068743	C22orf5	1
BC068750	SH3BP4	1
BC068758	ZNF507	1
BC068760		1
BC068770	STAG1	1
BC068778	NEK4	1
BC068780		1
BC068782		1
BC068797	HEBP1	1
BC068802		1
BC068806	FUT10	1
BC068810	LDLRAP1	1
BC068831	PLCG1	1
BC068835	SLC6A14	1
BC068847		1
BC068900	TPP1	1
BC068920	RND3	1
BC068924		1
BC068926	MAP2K5	1
BC068930	PCGF2	1
BC068931	PRKAR2B	1
BC068947		1
BC068952	EML2	1
BC068967	TRIP12	1
BC068972		1
BC070538	RAP2B	1
BC070543	AQP3	1
BC070547	HES1	1
BC070565	RCOR2	1
BC070569	GTF2F1	1
BC070590		1

BC070607	ZDHHC20	1
BC070610	ZNF499	1
BC070614	UBE2E3	1
BC070641		1
BC070645	FAM109A	1
BC070648	SEPT11	1
BC070650	SNX1	1
BC070666	EPB41L4A	1
BC070668	APP	1
BC070670		1
BC070701		1
BC070710	MAX	1
BC070723	TSPAN17	1
BC070743	REV1L	1
BC070747	PIAS2	1
BC070758	RAB11FIP5	1
BC070784	SASS6	1
BC070788	LLGL1	1
BC070797	RND3	1
BC070801	QKI	1
BC070803	KLF11	1
BC070837	PLCG1	1
BC070838		1
BC070842		1
BC070976	DCTN6	1
BC070978	BCKDK	1
BC071000	AGPAT4	1
BC071005		1
BC071011	KBTBD2	1
BC071012	PDK1	1
BC071015		1
BC071042	CYP2C18	1
BC071046		1
BC071048	PBX2	1
BC071059	ALDH1A2	1
BC071067	HNRPA0	1
BC071068	RAB2B	1
BC071089	NAGA	1
BC071097	ANXA5	1
BC071159	GPNMB	1
BC072035		1
BC072049	C9orf28	1
BC072056	APEX1	1
BC072059	SLC44A2	1
BC072062	ACP5	1
BC072082	MTHFD2	1
BC072084	FREQ	1
BC072119	VANGL2	1
BC072123	SOX17	1

BC072148		1
BC072174		1
BC072191	ZNF217	1
BC072204	LIMS1	1
BC072219	NPHP1	1
BC072231	ARL8B	1
BC072252	CTSD	1
BC072257		1
BC072259		1
BC072271	GUCY1B3	1
BC072272	CD81	1
BC072277		1
BC072304	TGM2	1
BC072345	FAM113A	1
BC072348	LOH11CR2A	1
BC072356	ZDHHC2	1
BC072362	TTC21B	1
BC072364	AMPH	1
BC072732	CBLB	1
BC072744	DUSP22	1
BC072752	FREQ	1
BC072769	RGS4	1
BC072786	SURF4	1
BC072787	CLIC4	1
BC072800	CD9	1
BC072806	MAB21L1	1
BC072810	RNF146	1
BC072814	CYFIP2	1
BC072824	FAM3C	1
BC072841	FN1	1
BC072852	SURF5	1
BC072875	HOOK3	1
BC072910	KAZALD1	1
BC072914		1
BC072934	PPM1D	1
BC072937		1
BC072945	GPR157	1
BC072951		1
BC072956	SNRPE	1
BC072963	TARBP2	1
BC072967		1
BC072969		1
BC072971		1
BC072991	SIRT6	1
BC072995		1
BC073053	CRP	1
BC073112	TYK2	1
BC073180		1
BC073185		1

BC073205		1
BC073210	MAD1L1	1
BC073229		1
BC073233		1
BC073236		1
BC073237	PARD6G	1
BC073243		1
BC073254	FHL3	1
BC073257	TSPAN4	1
BC073263		1
BC073266		1
BC073267	ENTPD1	1
BC073272	H2AFY2	1
BC073276	CHIT1	1
BC073279		1
BC073296		1
BC073301	TSPAN18	1
BC073303		1
BC073307	VGLL4	1
BC073311		1
BC073318	LCP2	1
BC073319		1
BC073321		1
BC073324	RAPGEF1	1
BC073337		1
BC073347		1
BC073348		1
BC073349	ADRB2	1
BC073367		1
BC073390	CLDN5	1
BC073392		1
BC073396	FNBP1	1
BC073407	GJA1	1
BC073409	C1QB	1
BC073412	GIT2	1
BC073413	C2orf4	1
BC073428	FGFR3	1
BC073429	BPGM	1
BC073465	SERINC3	1
BC073471		1
BC073475	SESTD1	1
BC073479	PITX2	1
BC073488	BCAM	1
BC073491		1
BC073494	SOX4	1
BC073495	PPP1R14B	1
BC073498		1
BC073530	BIN1	1
BC073545	CYBB	1

BC073550	ACP5	1
BC073559	XYLT1	1
BC073570	ACTR10	1
BC073577		1
BC073590	KIAA1166	1
BC073600		1
BC073603	CXCR4	1
BC073605	SPRY2	1
BC073606		1
BC073628		1
BC073642	AASS	1
BC073652	CCT4	1
BC073659		1
BC073675		1
BC073677	GTF2F2	1
BC073678		1
BC073726	KIFAP3	1
BC073738	C20orf55	1
BC074109	SEC22L2	1
BC074116		1
BC074121	GGCX	1
BC074123		1
BC074142		1
BC074168	NEU1	1
BC074173	SLC16A3	1
BC074239		1
BC074243	SCAMP5	1
BC074249	TSPAN5	1
BC074252		1
BC074257	FMNL1	1
BC074268	C18orf43	1
BC074275	NXN	1
BC074298		1
BC074307	PRNP	1
BC074309	KRT17	1
BC074313	ALCAM	1
BC074326		1
BC074342		1
BC074346	CCDC50	1
BC074347	TRPC3	1
BC074353	PHACTR1	1
BC074356	EDG1	1
BC074369	GGT1	1
BC074374	PAICS	1
BC074379	RASGEF1A	1
BC074383	CCDC28B	1
BC074390	CADPS	1
BC074392		1
BC074403		1

BC074404	NBL1	1
BC074410		1
BC074418	CBX1	1
BC074421	MAB21L1	1
BC074424		1
BC074435		1
BC074476	RABL2B	1
BC074477	EMP1	1
BC074481		1
BC074483	CCDC53	1
BC074489	CNFN	1
BC075132	FKBP14	1
BC075148		1
BC075151	ANXA1	1
BC075153	GLT8D1	1
BC075160	SULT1C1	1
BC075161	ETS1	1
BC075168		1
BC075182	RHPN2	1
BC075188		1
BC075209		1
BC075217	NIPSNAP3A	1
BC075223	RGS10	1
BC075228		1
BC075230		1
BC075234		1
BC075244		1
BC075248	STMN2	1
BC075249	NDRG4	1
BC076715	SMARCA5	1
BC076721	FGF13	1
BC076724	FAM44B	1
BC076737	CCNJ	1
BC076739	KIAA1826	1
BC076742	F2	1
BC076776	PLEKHA1	1
BC076779	MAPKBP1	1
BC076801	FAM26B	1
BC076802		1
BC076827	NT5DC2	1
BC076830		1
BC076836		1
BC076844		1
BC076851		1
BC076858		1
BC077169	TIA1	1
BC077174		1
BC077221	ACTC	1
BC077226	YPEL5	1

BC077253	GPM6B	1
BC077264	ETS2	1
BC077278	SYK	1
BC077279		1
BC077285	CTSS	1
BC077286		1
BC077287	HMHA1	1
BC077289		1
BC077300	PAG1	1
BC077302		1
BC077312	CLTB	1
BC077355	SNN	1
BC077380	DACT1	1
BC077400	UBXD4	1
BC077431	TMC7	1
BC077450	ZNF161	1
BC077476	INA	1
BC077477		1
BC077489	YPEL1	1
BC077498		1
BC077503	SNAG1	1
BC077505	GPSM1	1
BC077539	PTP4A1	1
BC077559	PDGFB	1
BC077570	JAK1	1
BC077577	AQP3	1
BC077587	SAP130	1
BC077597	SH3GLB2	1
BC077601	B4GALT3	1
BC077611		1
BC077620	WDR37	1
BC077629		1
BC077648	CLEC10A	1
BC077649	KRT12	1
BC077701	FCHO1	1
BC077738	PURA	1
BC077740	MBD3	1
BC077767	CHRD	1
BC077770	HNRPH1	1
BC077778	GNG7	1
BC077792	CSF3R	1
BC077798	HPCAL1	1
BC077848		1
BC077852		1
BC077854		1
BC077862	KCTD1	1
BC077864	SERPINE2	1
BC077870	MMP14	1
BC077871	GPR137B	1

BC077873	C9orf19	1
BC077878		1
BC077893		1
BC077902	CYBA	1
BC077905	ATP2B2	1
BC077906		1
BC077913	RDH10	1
BC077915		1
BC077919		1
BC077925	PFC	1
BC077938	CBFB	1
BC077960	PRTFDC1	1
BC077961	CD63	1
BC077967	FNDC3A	1
BC078007	HIRA	1
BC078012	RASGRP2	1
BC078013	YTHDF3	1
BC078057		1
BC078069		1
BC078071	GUK1	1
BC078089		1
BC078114	CCDC69	1
BC078116	SEMA4C	1
BC078133		1
BC078136	MMP17	1
BC078138		1
BC078467	GMFB	1
BC078517	RGS1	1
BC078523	SERPINA1	1
BC078525	IXL	1
BC078527	IGSF4C	1
BC078557		1
BC078579	PSKH1	1
BC078612	NME3	1
BC079718	ARRDC2	1
BC079733		1
BC079742		1
BC079762		1
BC079763	UAP1L1	1
BC079775	RAP1GDS1	1
BC079779	MAPT	1
BC079814	PIK3C2A	1
BC079817	RCSD1	1
BC079826	MR1	1
BC079829	DNAJB1	1
BC079830	PIN4	1
BC079918	FAM107B	1
BC079971	SLC15A4	1
BC079972	TCF7L2	1

BC079978	FUT8	1
BC079984		1
BC079985		1
BC079989		1
BC079998	SIRT5	1
BC079999	SNRPD2	1
BC080006	GALNT11	1
BC080012		1
BC080018	TRAF4	1
BC080021	ASAH1	1
BC080034		1
BC080038	TMEM26	1
BC080040		1
BC080041	GRK6	1
BC080043	MAP4K5	1
BC080054	LDHB	1
BC080062		1
BC080085	EDIL3	1
BC080090	OGDHL	1
BC080093		1
BC080097		1
BC080099	CD276	1
BC080111		1
BC080168	NEGR1	1
BC080377	APCDD1	1
BC080382	BMP1	1
BC080388	EMILIN2	1
BC080392	SEMA3D	1
BC080399		1
BC080402	ZNRF1	1
BC080419	IFT52	1
BC080997	PSAP	1
BC081002		1
BC081003	SDCBP	1
BC081037	TGIF2	1
BC081038	TRIM28	1
BC081047	SEPT11	1
BC081051	PIP5K2B	1
BC081059	ATP6V0D1	1
BC081076	SLC2A6	1
BC081080	CD37	1
BC081114	P4HA2	1
BC081134	SAV1	1
BC081135	CCNI	1
BC081140	BASP1	1
BC081144		1
BC081151	RAB20	1
BC081154	PSMD13	1
BC081160	ATP6V0B	1

BC081167	TIMP2	1
BC081168	DDAH1	1
BC081172	GDI1	1
BC081176	LHFPL3	1
BC081189	PLTP	1
BC081193		1
BC081227	TMEM111	1
BC081240		1
BC081241		1
BC081243		1
BC081245	COPS8	1
BC081255	SMARCD2	1
BC081256		1
BC081276	NEFH	1
BC081281		1
BC081284		1
BC082350		1
BC082364	AXIN2	1
BC082395	PPP1R14A	1
BC082399		1
BC082413	IGLL1	1
BC082414		1
BC082432	TCF12	1
BC082440	LRRC14	1
BC082446		1
BC082447	SRGAP2	1
BC082472	GPBAR1	1
BC082475	SNAP25	1
BC082476	COMT	1
BC082477	PTK9L	1
BC082529	RENBP	1
BC082531		1
BC082661	CYSLTR1	1
BC082673		1
BC082674	CLDN19	1
BC082678	C2orf32	1
BC082682	RABAC1	1
BC082687		1
BC082694		1
BC082695		1
BC082699		1
BC082703		1
BC082704		1
BC082709	CCR2	1
BC082837	SLC44A1	1
BC082842	PDK1	1
BC082892		1
BC082928		1
BC082931	CTSZ	1

BC082935	PPP1R1A	1
BC082941	DBN1	1
BC082950	PPGB	1
BC083012	TSPAN4	1
BC083015	SIGLEC12	1
BC083017		1
BC083029		1
BC083033		1
BC083034		1
BC083048	MFAP4	1
BC084060	ELF2	1
BC084067		1
BC084082	CTBP1	1
BC084083	SHKBP1	1
BC084089	NID2	1
BC084099	HISPPD1	1
BC084111	KIAA1826	1
BC084112		1
BC084114	PAQR4	1
BC084120	HPD	1
BC084122		1
BC084198	NRP1	1
BC084216	ORMDL1	1
BC084217		1
BC084219	CCT6A	1
BC084227	PHACTR2	1
BC084231		1
BC084233	TMEM110	1
BC084243	EOMES	1
BC084246		1
BC084257	GCNT1	1
BC084281	SNAG1	1
BC084298	TEX10	1
BC084307		1
BC084310	CITED2	1
BC084314	CCT4	1
BC084339	WDR37	1
BC084356		1
BC084358		1
BC084360	CD53	1
BC084362		1
BC084363		1
BC084379	IGLL1	1
BC084386	SCAP2	1
BC084391		1
BC084395	NINJ2	1
BC084408		1
BC084409		1
BC084414	BHMT	1

BC084419	NPL	1
BC084432		1
BC084599	BTC	1
BC084611	CD44	1
BC084615		1
BC084662		1
BC084665	P2RY12	1
BC084736	SULT1C1	1
BC084746		1
BC084768	KCTD12	1
BC084775		1
BC084782	PLXNA1	1
BC084787		1
BC084788	TMBIM1	1
BC084792	OLFM2	1
BC084795	SH3GL3	1
BC084797	GNB4	1
BC084801		1
BC084803		1
BC084806	SCG2	1
BC084811	DUSP23	1
BC084814	C9orf58	1
BC084819	FGL2	1
BC084875	IGLL1	1
BC084882	OPN1SW	1
BC084885	NIPA2	1
BC084886	RGS4	1
BC084912		1
BC084923	GNAI2	1
BC084924	C9orf19	1
BC084941	GLIS2	1
BC084957		1
BC084960	ADARB1	1
BC084972	ZFHX1B	1
BC085022	NHS	1
BC085024		1
BC085056	PYCARD	1
BC085060		1
BC085063	CSF2RB	1
BC085074	PLOD3	1
BC085220		1
BC085223	VIM	1
BC085226	GLT25D1	1
BC086263		1
BC086268	UBAP2	1
BC086271	CPT1B	1
BC086287		1
BC086289		1
BC086305		1

BC086461	TNIP1	1
BC086466		1
BC086468	C10orf38	1
BC086473		1
BC086475	TCF4	1
BC086634	JARID2	1
BC087295	CMTM3	1
BC087299		1
BC087307	EXOSC4	1
BC087320	NME4	1
BC087334		1
BC087347	TGFBI	1
BC087372		1
BC087383		1
BC087391	SCPEP1	1
BC087400	ANKRD45	1
BC087402		1
BC087415		1
BC087423		1
BC087460	WNT4	1
BC087464		1
BC087471		1
BC087481		1
BC087516		1
BC087519	SS18L2	1
BC088667	M6PR	1
BC088671	WDR71	1
BC088682		1
BC088693		1
BC088707		1
BC088715	SAP130	1
BC088790	GPX7	1
BC088804	C8orf42	1
BC088805		1
BC088827	MED8	1
BC088909	SH3GL2	1
BC088915	RXRG	1
BC088942		1
BC088945	CYYR1	1
BC088947	ACVR1	1
BC088952	TRAM2	1
BC088957	CHST12	1
BC088967	B3GNT5	1
BC089131	BAMBI	1
BC089137		1
BC089138	ENPP2	1
BC089169	AK7	1
BC089172	SLC18A1	1
BC089173	KLHL17	1

BC089181	COL6A2	1
BC089182		1
BC089198		1
BC089247	WDR69	1
BC089259		1
BC089264		1
BC089265	C1orf52	1
BC089272	PTTG1IP	1
BC089283	SOX3	1
BC089285	C6orf134	1
BC090055		1
BC090161		1
BC090217	C1orf102	1
BC090223		1
BC090231	GPC4	1
BC090252	GNAO1	1
BC090253	PCCA	1
BC091719	MNT	1
BC092034	ASB7	1
BC092118	RNASEH1	1
BC092120	ZNF585A	1
BC092158		1
BC092316		1
BC092325		1
BC092329		1
BC093534		1
BC093541		1
BC093570	PIGW	1
BC094085		1
BC094114		1
BC094136	MTAP	1
BC097583	UAP1L1	1
BC097600	FSCN1	1
BC097728	AGTRAP	1
BC097748	SPIN	1
BC097784	SNX30	1
BC097855	HNRPD	1
BC097925	C11orf46	1
BC098974	CMKOR1	1
BC099278	OTC	1
BC099326	HES5	1
BC099353	SLC36A4	1
BE026027		1
BE026109		1
BE026509		1
BE131768		1
BE189070		1
BE189714		1
BE192088		1

BE505174		1
BE505678		1
BE506346		1
BE506594	STIM2	1
BE507181		1
BE507589	HAL	1
BE508825	VSIG4	1
BE509179		1
BE509350	CD58	1
BE575410		1
BE575901	ANP32E	1
BE576135	COMP	1
BE576624		1
BE576657	RASA2	1
BE669221	PCGF3	1
BE679390		1
BE679528		1
BE679557		1
BE679591		1
BE680031		1
BE680067		1
BF048004		1
BF072310		1
BF231668	RAB34	1
BF231930		1
BF232099	SEMA3A	1
BF427048		1
BF427443	STK11	1
BF610645		1
BF610679		1
BF611398	UGCGL1	1
BF611979		1
BF612190		1
BF612406		1
BF612440		1
BF612692		1
BF613269		1
BF613768		1
BF614085		1
BF614679		1
BF615077	FUT8	1
BF615400		1
BF615463		1
BF615469	SH3BP4	1
BF615536		1
BF615812	HBE1	1
BF615988		1
BG016741	IMPDH2	1
BG016865		1

BG017699	AHI1	1
BG017718	TMSL3	1
BG017842		1
BG018663		1
BG018724		1
BG018753		1
BG018769	LFNG	1
BG018842		1
BG022174	PPP1R9B	1
BG022306		1
BG022421		1
BG022428		1
BG022440		1
BG022582	IGJ	1
BG037915		1
BG037975		1
BG038016		1
BG038061		1
BG038211	VAT1	1
BG038337		1
BG159797	GPR23	1
BG159957	ZNF484	1
BG160137		1
BG160237		1
BG160459	HBE1	1
BG160506		1
BG160708		1
BG161387		1
BG161391		1
BG161397		1
BG162094	SSBP3	1
BG162303	TNS3	1
BG162394		1
BG162413		1
BG162643		1
BG162675		1
BG163156	KCNE3	1
BG233593	TPR	1
BG234127	LTBP3	1
BG234149		1
BG234611		1
BG264196	HBA2	1
BG264233		1
BG264321		1
BG264344	GLRX	1
BG345217		1
BG364947	PRSS35	1
BG407525		1
BG410073	WDR41	1

BG413880		1
BG439425		1
BG439844		1
BG513848		1
BG553375		1
BG553396		1
BG555764		1
BG578993		1
BG657077	GPR17	1
BG729801		1
BG730106		1
BG730432		1
BG730586		1
BG730630		1
BG730933	NKD1	1
BG814570	APBA1	1
BG893369		1
BI068185		1
BI313803		1
BI313886	KLHL7	1
BI314838		1
BI315029		1
BI347717	HBE1	1
BI441612		1
BI445881		1
BI446334		1
BI448805		1
BI478163		1
BJ029022	ANKRD46	1
BJ030478		1
BJ030698		1
BJ030923	C1orf78	1
BJ030986		1
BJ032419	ARL5B	1
BJ034583		1
BJ034898		1
BJ035117		1
BJ037405		1
BJ038426	SAMD6	1
BJ038635		1
BJ039688		1
BJ042570	RNF24	1
BJ046282		1
BJ047296	LRRC54	1
BJ050974		1
BJ052099		1
BJ055135		1
BJ057306	C14orf1	1
BJ057644	DNAJB6	1

BJ058524		1
BJ059247		1
BJ060214		1
BJ060417		1
BJ061386		1
BJ061488		1
BJ061785		1
BJ063104		1
BJ063258		1
BJ063903	FAM107B	1
BJ064568		1
BJ065916		1
BJ066067		1
BJ066411	XPC	1
BJ066554		1
BJ067538	PDE2A	1
BJ068253	C3orf38	1
BJ068535		1
BJ070228		1
BJ071394	HSF2	1
BJ072242		1
BJ072373	SARDH	1
BJ073152		1
BJ073273		1
BJ073801		1
BJ074424		1
BJ077113	ZNF420	1
BJ078216		1
BJ078526		1
BJ081049		1
BJ084667		1
BJ084787		1
BJ085145	ORMDL2	1
BJ085893		1
BJ088082	RNF130	1
BJ088669	RAB20	1
BJ096279		1
BJ612287		1
BJ612498		1
BJ612923	ZNF569	1
BJ616349		1
BJ617381		1
BJ619086		1
BJ627001	PRX	1
BJ628972		1
BJ630546		1
BJ630946	ME2	1
BJ631095		1
BJ636788		1

BJ638827	RAB20	1
BJ638911		1
BJ640299	RHOB	1
BM180020		1
BM180861		1
BM191279		1
BM191696	ICAM5	1
BM261189		1
BM928912		1
BM928954	SLAMF8	1
BM928981	PVRL3	1
BM928982		1
BM928998		1
BP677676		1
BP682601		1
BP684279		1
BP685122		1
BP685880		1
BP686017		1
BP686071	DENND2C	1
BP687487		1
BP687629		1
BP688782		1
BP689354	C5	1
BP689420		1
BP689799		1
BP690724		1
BP691913		1
BP692816		1
BP692999		1
BP693239		1
BP694403		1
BP694998		1
BP696539		1
BP696833	RIC8A	1
BP697143		1
BP697571	TPBG	1
BP697688		1
BP698662	HBXAP	1
BP698716		1
BP699151		1
BP699378		1
BP699483		1
BP699596		1
BP701161		1
BP701259		1
BP701946		1
BP702886		1
BP703429		1

BP703709		1
BP704246	C9orf40	1
BP704537		1
BP704574	CDC42SE2	1
BP705893		1
BP706611		1
BP706688	ANKRD26	1
BP708254		1
BP708255		1
BP709295		1
BP709723		1
BP710184		1
BP715062		1
BP716170		1
BP720937		1
BP721556		1
BP722273		1
BP730276		1
BP731713		1
BP740062		1
BP740064		1
BP740251		1
BP741831		1
BQ383675		1
BQ386276		1
BQ388100		1
BQ397842		1
BQ398810		1
BQ399739		1
BQ724121		1
BQ725833		1
BQ727319		1
BQ728269		1
BQ729865		1
BQ730542		1
BQ730716	NXN	1
BQ730753	KIF26A	1
BQ730854	HINT3	1
BQ730962		1
BQ731009		1
BQ731442		1
BQ731780		1
BQ732561	DGKD	1
BQ732572	RP1L1	1
BQ732897		1
BQ732917		1
BQ733202		1
BQ733226		1
BQ733689		1

BQ733975		1
BQ734359		1
BQ734561	KCNE1L	1
BQ735488	EP400	1
BQ735962	COL8A1	1
BQ736298		1
BQ736650	TXNDC	1
BQ737351		1
BU152721		1
BX842722	C1orf149	1
BX842808	ASAH3L	1
BX842824	NAALAD2	1
BX842912		1
BX843406		1
BX843561	WDR41	1
BX843583	CRYAB	1
BX843661		1
BX843873		1
BX844003		1
BX844131		1
BX844253		1
BX844295		1
BX844446	PPP1R14A	1
BX844450	C16orf48	1
BX844526		1
BX844554	OVCH1	1
BX844628	EBI3	1
BX844666		1
BX844667	KLF13	1
BX844877	EPPK1	1
BX845344	ALDH1A2	1
BX845355	DOCK10	1
BX845568	BAX	1
BX845693	PDGFRA	1
BX845823		1
BX845870		1
BX845902		1
BX846187	C8orf35	1
BX846232		1
BX846234		1
BX846329	AGPAT1	1
BX846572		1
BX846575		1
BX846603		1
BX846631		1
BX846825		1
BX847105	PLTP	1
BX847357		1
BX847406		1

BX847588	MARCH3	1
BX847771		1
BX847776		1
BX847839		1
BX847871		1
BX847966		1
BX848061		1
BX848095		1
BX848111	RSU1	1
BX848180	TCTEX1D1	1
BX848215		1
BX848237	PHC1	1
BX848258		1
BX848320		1
BX848330		1
BX848354	SYN3	1
BX848422		1
BX848455		1
BX848475		1
BX848480		1
BX848644	CD38	1
BX848749	GPR23	1
BX848766	TSC1	1
BX848880	ARSB	1
BX848893	PGLYRP1	1
BX848927		1
BX849077		1
BX849085		1
BX849223	ATCAY	1
BX849309		1
BX849398		1
BX849418	GPR137C	1
BX849488		1
BX849724	ZNF436	1
BX849960		1
BX850038		1
BX850117		1
BX850169	A4GNT	1
BX850179		1
BX850302		1
BX850461	ATHL1	1
BX850630	COX15	1
BX850674	C20orf19	1
BX850685		1
BX850702	C10orf38	1
BX850931		1
BX850954		1
BX851025	NUBPL	1
BX851035	C17orf62	1

BX851075	PRSS27	1
BX851139		1
BX851196		1
BX851320		1
BX851375	PPFIBP2	1
BX851406	FBXO22	1
BX851536		1
BX851570	IL8	1
BX851623		1
BX851652	TRIM16	1
BX851828		1
BX851854	ING1	1
BX851861	SLC38A6	1
BX852154		1
BX852342		1
BX852352		1
BX852372	LLGL1	1
BX852440		1
BX852823		1
BX852957		1
BX853189	HOXC5	1
BX853225		1
BX853254		1
BX853276		1
BX853414	MYO18A	1
BX853433		1
BX853485		1
BX853527	WDR52	1
BX853578	PIN4	1
BX853616		1
BX853754	CKAP4	1
BX853773	LY9	1
BX853841		1
BX853985		1
BX854030		1
BX854048	RARRES2	1
BX854059		1
BX854457		1
BX854638		1
CA971610		1
CA971861	CSRP2	1
CA972148		1
CA973023	MAPK7	1
CA973076	PAPSS1	1
CA973750		1
CA974036		1
CA974443	TH1L	1
CA974505	THRAP6	1
CA980900		1

CA981378	KIAA1429	1
CA981854		1
CA982052		1
CA982120	ZNF300	1
CA982525		1
CA982783	PEO1	1
CA982820		1
CA983203		1
CA983391	SEPT9	1
CA983547	MED19	1
CB558803	CAT	1
CB560268		1
CB562980	TTC8	1
CB563548	TMSL3	1
CB592993		1
CB755900	CAPN7	1
CB941351	PALM2	1
CB941493	MCAM	1
CB942680		1
CB943089	C20orf172	1
CB943170		1
CB943171		1
CB943529	GIT2	1
CB943716		1
CB944397		1
CB944409		1
CB944643		1
CB944946		1
CB945344		1
CB983966		1
CD099389		1
CD100831		1
CD252706		1
CD252842		1
CD253566		1
CD253823		1
CD253832		1
CD253944		1
CD253973	TLN2	1
CD255960	VPREB3	1
CD301233		1
CD301757		1
CD302319		1
CD302391		1
CD302825		1
CD303201		1
CD303355		1
CD303400		1
CD324647		1

CD325847	FAM62A	1
CD325861		1
CD326328		1
CD326663		1
CD327121		1
CD327154		1
CD327260		1
CD328137		1
CD328493		1
CD329717		1
CD329863		1
CD361577		1
CD811084		1
CD811314		1
CF270408		1
CF270488		1
CF270683		1
CF271689		1
CF271792	C14orf49	1
CF271887		1
CF283142		1
CF283196		1
CF283551		1
CF283947		1
CF284349		1
CF284516		1
CF285070	VMO1	1
CF285539		1
CF285540		1
CF285606		1
CF285650		1
CF286277		1
CF286572		1
CF286639		1
CF286648		1
CF286684		1
CF286923		1
CF287096		1
CF287379		1
CF287507		1
CF287532		1
CF288442		1
CF289954	MXRA7	1
CF290001		1
CF290385		1
CF290479		1
CF290740		1
CF342082		1
CF342159		1

CF342308		1
CF520724	SERINC2	1
CF521258	PLAC8	1
CF521264	METTL1	1
CF521551		1
CF521858		1
CF522012		1
CF522156	C1orf149	1
CF522601		1
CF546720		1
CF547202		1
CF547458		1
CF547820		1
CF547880		1
CF548267		1
CF548599	GNGT1	1
CF548836		1
CF548877		1
CF548888		1
CF548959		1
CK742889		1
CK796526		1
CK796688		1
CK796938	ADNP	1
CK796998	SNAPC5	1
CK797035		1
CK797064	PSMB2	1
CK797066		1
CK797205		1
CK797468		1
CK797783	S100A11	1
CK797847		1
CK797904	C3orf38	1
CK797913		1
CK797924	NPC2	1
CK798017	SURB7	1
CK798028		1
CK798046		1
CK798049		1
CK798118	SH3BGRL3	1
CK798340		1
CK798368		1
CK798371		1
CK798428		1
CK798446		1
CK798479		1
CK798484		1
CK798533	TMEM107	1
CK798599	PRDX2	1

CK798617	COX4I2	1
CK798694		1
CK799129		1
CK799314		1
CK799643	ERICH1	1
CK799983		1
CK800034	C14orf93	1
CK800037	CASP6	1
CK800055		1
CK800382	IFITM2	1
CK800686	HELZ	1
CK803867	C1orf121	1
CK804551		1
CK804838		1
CK805128	FKBP1A	1
CK805172		1
CK805269	FTH1	1
CK805510	KCNE1L	1
CK807007		1
CK807439	IHPK1	1
CK815995		1
CN325429		1
CO385591		1
CV072922		1
CV072994		1
CV077928		1
CV079029		1
CV079892	EMR3	1
CV080315	PDLIM4	1
CX130081	FBXW7	1
CX132045		1
CX134065		1
CX134775		1
CX134846		1
CX135377		1
D14400	ITPR1	1
D14667	PARP1	1
D29796	CFB	1
D38175	PLXNA1	1
D49373	CFB	1
D78003		1
D83476	BMP1	1
D83649	SOX7	1
D87209	SOX11	1
DQ096881		1
DQ096892		1
DR719140		1
DR720479		1
J05180	TGFB1	1

L15586		1
L19881	APCS	1
L20728	MR1	1
L25856	HOXA1	1
L29495	PIM3	1
L38675	NKX2-3	1
L39213	SHH	1
M18350		1
M22834	H1F0	1
M24752	HOXA7	1
M25866	ZNF84	1
M27502	FYN	1
M35362	THRB	1
M58019	MR1	1
M76410	SERPINA1	1
M76566	GATA1	1
M76710	NCAM1	1
M81683	ETS2	1
M84162	PPARD	1
M94969	ABI2	1
M98807	NOG	1
S61773	INHBB	1
S80988	RNF36	1
U04707	CDH1	1
U08407	ARG2	1
U12683	ITGA5	1
U14169	CPEB1	1
U18775	TLE1	1
U19618	FGB	1
U23535	SCNN1A	1
U24433		1
U35408	KLF9	1
U37375		1
U37376	MAMDC2	1
U39929	CBX4	1
U41761	SDC3	1
U41824	MMP13	1
U41855	C5orf13	1
U41856	DPP4	1
U41858	CRHBP	1
U42461	GLI3	1
U54497	ITGA4	1
U60093	RBPSUH	1
U60424	IGLL1	1
U64442	APC	1
U66288	WNT2B	1
U68059	FRZB	1
U68387	MEIS1	1
U68724	MPO	1

U75996	EOMES	1
U77639	SMAD1	1
U77640	LFNG	1
U81958	BMPR2	1
U89265	KCND3	1
U95104	CYYR1	1
X12499	HOXC6	1
X14920		1
X52692	ETS1	1
X56870	MYC	1
X57675	CDH2	1
X61338		1
X61349	LOC90925	1
X65943	FGFR2	1
X66959	TCF3	1
X66979	FLI1	1
X68024	ZFX	1
X72957	NCL	1
X83962	TMEFF1	1
Y13069	DPYSL3	1
Z14997		1
Z27093	MMP11	1
AB008225	SLC5A11	2
AB022787	TYRO3	2
AB024524	ALDOC	2
AB046536	MTCH2	2
AB086829	KRT10	2
AB092377		2
AB093561	IQGAP1	2
AB114053		2
AB176536	LRRC54	2
AB176537	LRRC54	2
AF027728	CENPE	2
AF061833	ALDH1A1	2
AF100165	STK10	2
AF133036	TBX5	2
AF143489	ACAT2	2
AF146087	HES5	2
AF182522	PKP3	2
AF184090	ADFP	2
AF308152	P2RX4	2
AF338157	IRX4	2
AF421880	CACNA1S	2
AF465788		2
AJ007835	AHCY	2
AJ304990	CCNB3	2
AJ557446	KPNA2	2
AJ575267	ADH5	2
AJ575933	FUT2	2

AJ585762	ST3GAL2	2
AW147250		2
AW147428	NDUFS6	2
AW148041	COX7C	2
AW164955		2
AW199573	BRP44	2
AW199610	RNF152	2
AW199660	IFNGR2	2
AW199678		2
AW199690		2
AW199813	NDUFB11	2
AW199835		2
AW199896		2
AW200087	NDUFA1	2
AW200122	REEP6	2
AW200171	ATP5L	2
AW200202	CASP7	2
AW200217	MRPL37	2
AW200247	MIXL1	2
AW200411		2
AW200424	COX7C	2
AW200549		2
AW200552		2
AW200556	COX6C	2
AW200559	MRPL27	2
AW200613		2
AW200620		2
AW460440		2
AW632936		2
AW634109	UQCRQ	2
AW634424	ATP5J	2
AW634474	COX7A2	2
AW634635		2
AW635120		2
AW637956		2
AW641041		2
AW641159	BAX	2
AW645247		2
AW645440		2
AW646661		2
AW764580	KRT8	2
AW765260	MRPL46	2
AW766394		2
AW767401	DBI	2
AW767623	CRISP3	2
AW871903	PRRT1	2
AY114144	TNNT3	2
AY197550		2
AY271302		2

AY354455	NCK2	2
AY362041	SOD2	2
AY509892		2
AY534752	CDC14A	2
AY644401		2
BC041187	NR1I2	2
BC041194	HSD17B12	2
BC041231	TEX261	2
BC041234	PISD	2
BC041289	CAV3	2
BC041303	SLC25A1	2
BC041735	ADAMTS13	2
BC041742	B4GALT2	2
BC041754	FGA	2
BC042221	KCNJ16	2
BC042224	HNF4A	2
BC042245	GLDC	2
BC042262	KCNK1	2
BC042270	GMDS	2
BC042276	SHMT1	2
BC042294	SLC3A2	2
BC042304	ELOVL1	2
BC042344	TOM1L1	2
BC042356	STOM	2
BC042929	HMGCS1	2
BC042931	UQCRC2	2
BC043633	TBL2	2
BC043754	AARSD1	2
BC043756	ACSL1	2
BC043826	FAM3A	2
BC043834	SLC25A25	2
BC043836	GUP1	2
BC043859	SDHB	2
BC043868	PCYT1A	2
BC043896	ADSS	2
BC043974	TUBB2C	2
BC043991	ACO1	2
BC043999	SPR	2
BC044025	ATP6V1A	2
BC044037	DNAJC3	2
BC044053	ZNF622	2
BC044073		2
BC044274	ESRRA	2
BC044280	TMEM32	2
BC044291	DNM1L	2
BC044681	LASP1	2
BC044971	SLC7A8	2
BC044981		2
BC045001	GAMT	2

BC045003	IRF6	2
BC045019	MTHFD1	2
BC045028	SCARB2	2
BC045036	CPT2	2
BC045088	SERHL2	2
BC045119	ACAA2	2
BC045123	CKMT1B	2
BC045127	CYC1	2
BC045130	HSPA9B	2
BC045222	HADHB	2
BC045232	WDR23	2
BC045259	HSPA9B	2
BC045267	PALMD	2
BC045269	GOT1	2
BC046372	JMJD2B	2
BC046577	VDAC1	2
BC046656	RHOA	2
BC046660	DNAJA4	2
BC046683	RAB6A	2
BC046688	SLC7A8	2
BC046694	RDH16	2
BC046710	ABHD3	2
BC046711	SDFR2	2
BC046724	M6PRBP1	2
BC046739	ACTC	2
BC046740	ACSL1	2
BC046842	MYO1E	2
BC046843	RFK	2
BC046849	SLC25A3	2
BC046947	CASQ1	2
BC047257	MPP1	2
BC047973	GATM	2
BC048018	SDCBP	2
BC048227	PCK1	2
BC049292	COMT	2
BC049394	NDUFS1	2
BC053785	SLC31A1	2
BC053792	SULT1C2	2
BC053821	FBXL15	2
BC054146	CDC2	2
BC054147	MYL7	2
BC054203	DHCR7	2
BC054204	METAP1	2
BC054207	DEGS2	2
BC054229	APOB	2
BC054239	TM4SF4	2
BC054278	PRDX6	2
BC054281	HNMT	2
BC054290	GSTK1	2

BC054291	HSD11B1	2
BC054292	TXNDC9	2
BC054299	ACAA1	2
BC054307	STOM	2
BC054309	PRDX6	2
BC054310	RAB3IP	2
BC054315	MYBPH	2
BC054960	VIL1	2
BC054964	CAT	2
BC054984	ABHD6	2
BC054987	NAALADL1	2
BC054994	PEPD	2
BC055967	EBP	2
BC055995	OXNAD1	2
BC056018	GPSN2	2
BC056024	TCF2	2
BC056036	CLIC5	2
BC056042	TJP2	2
BC056096	XRCC6BP1	2
BC056098	NDUFS5	2
BC056102	C1orf21	2
BC056108	HADHSC	2
BC056120	PTER	2
BC056131	TRIM16	2
BC056136	TXNRD2	2
BC056853	SSR1	2
BC056855	FABP7	2
BC057298	DPP4	2
BC057708	MVP	2
BC057712	FLJ21106	2
BC057747	RAB35	2
BC057748	SPATS2	2
BC057751		2
BC058770	RBM5	2
BC059301	KCNJ15	2
BC059318	POR	2
BC059330	PEX11B	2
BC059787	ETFB	2
BC059961	HABP4	2
BC059972	PDK2	2
BC059978		2
BC059979	RHBG	2
BC059990	CYP2E1	2
BC059991	DGAT2	2
BC059994	TOMM70A	2
BC059996	HSDL2	2
BC059997		2
BC060011	GCKR	2
BC060329	TOB1	2

BC060346	SDSL	2
BC060356	CASP10	2
BC060365	STXBP6	2
BC060383	AKR1B1	2
BC060398	RIOK3	2
BC060401	RAB7	2
BC060415	PAPSS2	2
BC060446	SDHA	2
BC060449	EPB41L3	2
BC060455	CYP2C19	2
BC060470	SAMM50	2
BC060485	PKM2	2
BC060486	SLC37A4	2
BC060496	CYP3A4	2
BC060755	DDR1	2
BC061648	TSPAN3	2
BC061652	HSD11B1	2
BC061656	SAR1B	2
BC061667	APOA4	2
BC061676	BAIAP2L1	2
BC061682	ADH1C	2
BC061932	AKR1D1	2
BC061936	SYK	2
BC063272	SLC25A22	2
BC063726	FADS2	2
BC063727		2
BC063733	CAPN2	2
BC068626		2
BC068661		2
BC068666		2
BC068670		2
BC068686	TMEM51	2
BC068707	LAP3	2
BC068745	SESN2	2
BC068755	ACAT1	2
BC068798	TDRD7	2
BC068809		2
BC068856		2
BC068891	ACOX2	2
BC068897		2
BC068899	XPNPEP1	2
BC068932		2
BC068954		2
BC070522	SEC23B	2
BC070544	TMEM45B	2
BC070546	ANKH	2
BC070561		2
BC070564		2
BC070602	MFN2	2

BC070619	PPA2	2
BC070627	AP1M2	2
BC070630	PGAM1	2
BC070635	ARL5A	2
BC070643	SPTLC1	2
BC070655	HIGD1A	2
BC070657	ABCD3	2
BC070665	SLC25A10	2
BC070671	SLC34A2	2
BC070726	SLC39A9	2
BC070741		2
BC070762	APITD1	2
BC070782	MYO1B	2
BC070852	HOOK2	2
BC070868	PBP	2
BC070870	LRPPRC	2
BC070973		2
BC071002	AADAT	2
BC071009	GALNT4	2
BC071024		2
BC071043	SEC31L2	2
BC071073		2
BC071092		2
BC071106	ALDH3A2	2
BC071111	IRF6	2
BC071113		2
BC071122	GATA4	2
BC071136	DECR2	2
BC071154	TAX1BP3	2
BC072036		2
BC072040	SLC7A7	2
BC072042	DNAJB2	2
BC072124	ARVCF	2
BC072157		2
BC072175	SDFR2	2
BC072225	BCAR3	2
BC072247	HMGCL	2
BC072248		2
BC072281	TSPAN13	2
BC072287	PCK2	2
BC072314	FLAD1	2
BC072330		2
BC072340		2
BC072727	PGRMC1	2
BC072731	NDRG3	2
BC072751		2
BC072788		2
BC072803	SMYD1	2
BC072818	SLC41A1	2

BC072823	SESN3	2
BC072826		2
BC072909		2
BC072961	PRKAB1	2
BC072964	SNX12	2
BC072977	PAFAH2	2
BC073008	DNASE1L3	2
BC073043	PBP	2
BC073059		2
BC073110	PTPN3	2
BC073116	B3GNT7	2
BC073130	NIF3L1	2
BC073197		2
BC073213	OGDH	2
BC073217	NDUFB7	2
BC073239	CHCHD2	2
BC073240	MYO1D	2
BC073246		2
BC073248		2
BC073341	DHRS7	2
BC073377		2
BC073379	OSBPL2	2
BC073388		2
BC073389		2
BC073400	AHCY	2
BC073416	DHRS3	2
BC073433	MAL2	2
BC073458		2
BC073466		2
BC073496		2
BC073503	CIDEB	2
BC073518	CYP26A1	2
BC073572	NR1D1	2
BC073574	SCCPDH	2
BC073591		2
BC073643	UROD	2
BC073653	SLC29A2	2
BC073664	ALAD	2
BC073692	ARL6IP2	2
BC073693	TMEM15	2
BC073699		2
BC073703		2
BC073718	PDIK1L	2
BC073719		2
BC073722		2
BC073733	RTCD1	2
BC074120		2
BC074127		2
BC074131	CYP4V2	2

BC074134	CYB561	2
BC074139	GMPR2	2
BC074145	HSD17B4	2
BC074152		2
BC074162	HSD17B12	2
BC074181		2
BC074197	HMGCR	2
BC074198	ASRGL1	2
BC074201	DHDH	2
BC074206		2
BC074210	ADH1B	2
BC074256		2
BC074274		2
BC074303	GRTP1	2
BC074314	SCCPDH	2
BC074319		2
BC074322	TMEM56	2
BC074325		2
BC074341		2
BC074387	PCSK2	2
BC074429		2
BC074442		2
BC074446	NT5C2	2
BC074447	CCDC19	2
BC074465		2
BC075131	TPD52	2
BC075133	ECH1	2
BC075135		2
BC075136	DHRS4	2
BC075155	ADK	2
BC075171	CNDP2	2
BC075173		2
BC075178	SLC31A1	2
BC075202	SORD	2
BC075226		2
BC076642	PSPH	2
BC076646	PHYH	2
BC076718	GATA6	2
BC076719		2
BC076743		2
BC076745		2
BC076746		2
BC076747	POU3F4	2
BC076752	GLO1	2
BC076754	MPST	2
BC076770	MGAT1	2
BC076826		2
BC076840	PGM2	2
BC076843	NPPC	2

BC076848	PKP3	2
BC076857	PANK3	2
BC076860		2
BC076864		2
BC076865	GPHN	2
BC077194	GCDH	2
BC077199	SDHD	2
BC077210	ACADM	2
BC077224	KIF20A	2
BC077236	GYG1	2
BC077247		2
BC077260	CCNA2	2
BC077270	PAFAH1B1	2
BC077325		2
BC077334	CYB5A	2
BC077335		2
BC077371	FLRT3	2
BC077419	MYH4	2
BC077422	TAX1BP3	2
BC077436		2
BC077437		2
BC077457	QPRT	2
BC077479	CYP4B1	2
BC077484	PGAM1	2
BC077486	TMEM16E	2
BC077507	PHYH2	2
BC077516	RAB3IP	2
BC077532	PECR	2
BC077536	MRPL15	2
BC077556	FADS2	2
BC077572		2
BC077578	AP1M2	2
BC077580	B4GALT5	2
BC077618	ACAD9	2
BC077756	PFKM	2
BC077769		2
BC077788	SDF2L1	2
BC077793	OXSR1	2
BC077838		2
BC077877	ATF5	2
BC077894	OPRS1	2
BC077901	FHL1	2
BC077908	ALDH2	2
BC077917	LTB4DH	2
BC077922	DES	2
BC077936	RIOK3	2
BC077943	PI4K2B	2
BC077963		2
BC077977	HADH2	2

BC077984		2
BC078029		2
BC078055	SC5DL	2
BC078065		2
BC078066	EPHX2	2
BC078068	RHOU	2
BC078076	HCCS	2
BC078118	STRBP	2
BC078126		2
BC078134		2
BC078518	CRYM	2
BC078522		2
BC078524		2
BC078529		2
BC078533		2
BC078536		2
BC078569	NDUFS8	2
BC078570	PEMT	2
BC078591		2
BC078604		2
BC078619		2
BC078625	LENG9	2
BC079689	FOS	2
BC079694	HMGCS1	2
BC079708		2
BC079712		2
BC079714	NDFIP2	2
BC079717	ENPP4	2
BC079720	DNAJC5	2
BC079727		2
BC079736		2
BC079744		2
BC079789		2
BC079791		2
BC079976	TOR1A	2
BC079981	OACT5	2
BC079994	PTK9	2
BC080003	DPYD	2
BC080008		2
BC080019		2
BC080033	SUCLA2	2
BC080059	GMPPB	2
BC080065	CDR2	2
BC080102	TMEM93	2
BC080108	ELOVL2	2
BC080113	C14orf100	2
BC080116	C1QTNF4	2
BC080129	GLO1	2
BC081010	NDE1	2

BC081014	GTSE1	2
BC081043	AZIN1	2
BC081049		2
BC081056	ACADM	2
BC081057		2
BC081062	GCSH	2
BC081075	SLC6A19	2
BC081090	TIMM44	2
BC081113	PAK1	2
BC081124	IPMK	2
BC081141	RDH5	2
BC081159		2
BC081201		2
BC081210		2
BC081219	DOM3Z	2
BC081224		2
BC081269	AHCYL1	2
BC082349	GATA6	2
BC082351	ADFP	2
BC082357	NUDT4	2
BC082361	RHOU	2
BC082372	CIDEC	2
BC082381		2
BC082387	C10orf58	2
BC082388	C20orf112	2
BC082391		2
BC082394		2
BC082410	RNPEP	2
BC082429	DMD	2
BC082471		2
BC082626		2
BC082628	SYTL2	2
BC082648	IDI1	2
BC082651	IDH1	2
BC082653	CTH	2
BC082655	SCP2	2
BC082662		2
BC082684	APOA4	2
BC082698		2
BC082705	DDT	2
BC082712		2
BC082836	PCBD1	2
BC082861		2
BC082885		2
BC082902		2
BC082908	MYOZ2	2
BC082919	NUCB1	2
BC082922	SUCLG1	2
BC082939		2

BC082954		2
BC083009	C18orf55	2
BC083016	SREBF1	2
BC084078	C9orf52	2
BC084124	C1GALT1C1	2
BC084157		2
BC084237		2
BC084255		2
BC084256	ATP6AP1	2
BC084264	DHX32	2
BC084266		2
BC084267		2
BC084269		2
BC084277		2
BC084322		2
BC084325		2
BC084329	HIBADH	2
BC084341	FDXR	2
BC084366	NDUFA10	2
BC084371	KBTBD10	2
BC084384	FA2H	2
BC084392	PHCA	2
BC084430	NUDT12	2
BC084618	CYP4B1	2
BC084627		2
BC084660		2
BC084667		2
BC084752		2
BC084761	SLC35F2	2
BC084776	ACLY	2
BC084799		2
BC084826	GMFB	2
BC084841	DHFR	2
BC084850		2
BC084855		2
BC084928	KIF23	2
BC084931	KCNK5	2
BC084934	ENTPD4	2
BC084936	BLNK	2
BC084943	RHCG	2
BC084958		2
BC084966	UGCG	2
BC084981		2
BC085020	SLC25A20	2
BC085046		2
BC085047		2
BC085048		2
BC085059	TMEM41B	2
BC085065	NUAK2	2

BC085199	TMEM62	2
BC085208		2
BC085219	IL22RA2	2
BC086290		2
BC086297		2
BC086314		2
BC086464	SLC9A3R1	2
BC087294		2
BC087330		2
BC087349		2
BC087364		2
BC087377		2
BC087379		2
BC087380		2
BC087381		2
BC087387	LTB4DH	2
BC087397	STK19	2
BC087401		2
BC087421		2
BC087453		2
BC087494		2
BC087501		2
BC087621	DERA	2
BC087622	PPARA	2
BC088665	C9orf98	2
BC088685		2
BC088690	C18orf19	2
BC088699		2
BC088702		2
BC088706		2
BC088716		2
BC088717		2
BC088820	NDUFS7	2
BC088905	ALDH3A2	2
BC088922		2
BC088936	PBK	2
BC089120	DSC2	2
BC089129		2
BC089134		2
BC089165		2
BC089190		2
BC089273	MTHFS	2
BC090224	SULT1E1	2
BC091638	AP2A2	2
BC092017	CYB5B	2
BC092028	PPP1R3B	2
BC092100	TXNDC14	2
BC092312	KIAA1919	2
BC092341		2

BC092390		2
BC094074	PCCB	2
BC094201	NPEPL1	2
BC094210		2
BC094271	QDPR	2
BC097581		2
BC097634		2
BC097726		2
BE026392		2
BE026823	NMT1	2
BE131864		2
BE132052		2
BE188971		2
BE192407	HSD17B6	2
BE491189	MRPL41	2
BE491598		2
BE505878	DNAJB1	2
BE506967	NDUFA6	2
BE506980		2
BE507722		2
BE507901		2
BE507967		2
BE508558		2
BE508799	ANXA7	2
BE575428		2
BE575499		2
BE576109		2
BE576565		2
BE576620		2
BE680083		2
BE680570		2
BF024886		2
BF025190	DPP4	2
BF047259		2
BF047364	ATP5J2	2
BF047624	FNDC3A	2
BF072340		2
BF231809		2
BF611101	UQCR	2
BF611103		2
BF611222	COX7A2L	2
BF611511		2
BF612654		2
BF613503		2
BF614568		2
BF614590	DNAJA3	2
BF614639	ATPIF1	2
BG016658		2
BG017514	NDUFB2	2

BG017560		2
BG017589		2
BG017853		2
BG018014	FA2H	2
BG019049		2
BG020219		2
BG020414	ABHD14B	2
BG022668		2
BG022822		2
BG023031		2
BG037969	SNX3	2
BG160257		2
BG161411		2
BG163154		2
BG264524		2
BG264800	ACTB	2
BG346626		2
BG407870		2
BG409617		2
BG409814		2
BG438810		2
BG438927	RPL13A	2
BG486671		2
BG513473		2
BG514494		2
BG515438	SDHC	2
BG552014		2
BG552585	FIP1L1	2
BG552903		2
BG553561	KRT18	2
BG578422		2
BG579281	ANK1	2
BG730488		2
BG731178	CA4	2
BG810899		2
BG810957		2
BG811521		2
BG811799	DCI	2
BG811942		2
BG812314		2
BI446936		2
BI447707		2
BI448854		2
BI449890	NDUFA2	2
BJ028936	TMEM69	2
BJ031373		2
BJ031684	HK1	2
BJ035521		2
BJ038707		2

BJ039830		2
BJ041689	CLEC10A	2
BJ045456		2
BJ048218		2
BJ052687		2
BJ055556	RAB5B	2
BJ058051		2
BJ062152		2
BJ065503	GJB1	2
BJ068565	NAALAD2	2
BJ068590		2
BJ068686		2
BJ068771		2
BJ076956	MYL4	2
BJ078276	WDR39	2
BJ083148		2
BJ086892		2
BJ089483		2
BJ089988		2
BJ091734		2
BJ094084		2
BJ094105	AOX1	2
BJ094248		2
BJ094257		2
BJ096701		2
BJ613168	EIF2AK2	2
BJ613998	ATP5G3	2
BJ616136	B3GALT2	2
BJ617395		2
BJ618005		2
BJ624120		2
BJ624263		2
BJ631189	TM2D2	2
BJ631732		2
BJ633615	PLDN	2
BJ636812	SYCP2	2
BJ644027		2
BM179769		2
BM181057	FGA	2
BM191511		2
BM192882		2
BP674987	POR	2
BP682407		2
BP683015		2
BP683068		2
BP685925		2
BP687505		2
BP691865		2
BP692333		2

BP693983	MVK	2
BP698904		2
BP699428	EPHA7	2
BP700378		2
BP701163		2
BP703418		2
BP703629		2
BP703928		2
BP704434		2
BP706037	COX6C	2
BP706872		2
BP707097		2
BP707243		2
BP709638		2
BP719407		2
BP725976		2
BP727769		2
BP732492	MAK10	2
BP741589		2
BP741787		2
BQ384634	UBB	2
BQ385209	CKB	2
BQ386777		2
BQ388051	RAB43	2
BQ388273		2
BQ398256		2
BQ398490		2
BQ723944		2
BQ725085		2
BQ726613	SLC39A5	2
BQ727333		2
BQ731671		2
BQ733291		2
BQ733454		2
BQ734970	PDCD8	2
BQ735035		2
BQ735326		2
BQ735737		2
BQ736392		2
BQ736745		2
BQ737008	FAAH	2
BQ884366		2
BQ884377	EIF2S2	2
BQ884389	EIF4G3	2
BQ884420	ARHGAP17	2
BQ897327		2
BQ900459	BASP1	2
BU152749		2
BX842761	PRSS15	2

BX843339	EIF2AK2	2
BX843474		2
BX843538	WBSCR17	2
BX843871	STYK1	2
BX844087		2
BX844434		2
BX844523	ZP3	2
BX845098	MRPS31	2
BX845224		2
BX845249		2
BX845543		2
BX846284	MUC5B	2
BX846352	FDX1	2
BX846940	TOR1B	2
BX847137		2
BX847553	C20orf108	2
BX847608	MRPL19	2
BX847738		2
BX848034		2
BX848092		2
BX848535		2
BX848543		2
BX848856	INHBB	2
BX849020	STARD4	2
BX849263	COX5B	2
BX849277		2
BX849973		2
BX850024	HSD17B6	2
BX850131	MCCC2	2
BX850181	C3orf59	2
BX850234	BET1	2
BX850336		2
BX850459	C20orf22	2
BX850563	CLDN2	2
BX850733		2
BX850790	NIPSNAP1	2
BX851135	TMEM16J	2
BX851573	MGAT4B	2
BX852007		2
BX852046	GSTA1	2
BX852055	GRTP1	2
BX852056		2
BX852353		2
BX852379	C18orf55	2
BX852437	MFSD2	2
BX853035		2
BX853097		2
BX853488		2
BX853528		2

BX854027	LBR	2
BX854511		2
BX854775	CKAP2	2
BX855392		2
BX855428		2
CA971257		2
CA972414		2
CA972497	SSR4	2
CA972632		2
CA972715		2
CA973430	BAIAP2L2	2
CA973487	SPAG1	2
CA973541	OCIAD2	2
CA973597	SCP2	2
CA974238	ATP5G3	2
CA974265		2
CA974467	STARD10	2
CA980798	EIF3S2	2
CA980878	ZC3H11A	2
CA981199	HSD17B8	2
CA981310	OVOL2	2
CA981435	FNBP1L	2
CA981670		2
CA982252		2
CA982667		2
CA983003		2
CA983342		2
CA983600	ALDH6A1	2
CA983623		2
CB560198	MUC2	2
CB560308		2
CB561733		2
CB561882	TBC1D14	2
CB562051	MUC2	2
CB562055	MGAM	2
CB562834	VIL1	2
CB563002		2
CB564765	DNAJB1	2
CB565128		2
CB756001	LCT	2
CB756236		2
CB756731		2
CB941448		2
CB942817		2
CB943246		2
CB943372		2
CB944053		2
CD099668	VNN1	2
CD099938		2

CD100048		2
CD100577		2
CD100774		2
CD101209		2
CD101250	OCM	2
CD253356		2
CD254188		2
CD255843	PIR	2
CD256652		2
CD256759		2
CD301370	ELOVL5	2
CD301585	HMG2L1	2
CD301859		2
CD302254		2
CD302379		2
CD325272		2
CD325445		2
CD326370	BCORL1	2
CD326519		2
CD326805		2
CD328086		2
CD360704		2
CD361579	CRYGB	2
CD362829		2
CF271280		2
CF283568	CAMK2N1	2
CF286131		2
CF286172	PAFAH1B1	2
CF287253		2
CF288031		2
CF289962		2
CF520187		2
CF520476	FAM3B	2
CF520694		2
CF521010		2
CF521207		2
CF522107		2
CF522635	CLPB	2
CF547537	CHDH	2
CF547931		2
CF548021		2
CK796536	MGST1	2
CK796641		2
CK796707	PIGF	2
CK796739		2
CK796828	AKR7A2	2
CK796896		2
CK797282	ATP5O	2
CK797287		2

CK797441		2
CK797494		2
CK797592		2
CK797657		2
CK797701		2
CK797953		2
CK797961		2
CK798124		2
CK798516	STAMBP	2
CK798527		2
CK798748	C6orf125	2
CK798929	C10orf57	2
CK798956		2
CK799015		2
CK799019	TIMM13	2
CK799201		2
CK799249	GSTZ1	2
CK799329		2
CK799532	USP21	2
CK799837		2
CK799885	MGST3	2
CK799932	GRPEL1	2
CK800243	BZRP	2
CK800377	RER1	2
CK800385	NDUFB9	2
CK800436	COX5B	2
CK800472		2
CK800517		2
CK800585	TIMM8B	2
CK800619		2
CK800673		2
CK803819		2
CK803937		2
CK804869		2
CK804949		2
CK805158	PMVK	2
CK805329	JAGN1	2
CK805431		2
CK806759	TMEM4	2
CK807364		2
CN323707	ATP5H	2
CO385090		2
CO387976		2
CO388111		2
CV075669		2
CV081134	NDUFB9	2
CV121162		2
CV523251		2
CV523254	GNA14	2

CV523300	HOXA9	2
CX130901		2
CX131603	RSHL2	2
CX132068		2
D49543	INHBB	2
D49837	GUCY2C	2
DQ066918	GRIN1	2
DQ096958		2
DQ097000		2
DR716465	MOGAT2	2
L13702	GATA5	2
M59454	YBX2	2
M60680	CDC2	2
M80257	YBX2	2
M96858	CDC25C	2
U05001	NR5A2	2
U17608	ABCB1	2
U35728	TFAM	2
U41839	UMOD	2
U76636	CALB1	2
U91846	VDR	2
X56451	SRF	2
X82012	KIF4A	2
X93494	GCK	2
Z49827	HNF4A	2
AB003358	LYN	3
AF005083	ZNF346	3
AF042353		3
AF270484	TKTL2	3
AF272361	EBNA1BP2	3
AF343894	SMPX	3
AF417472	MYBPC3	3
AW200034	RNU3IP2	3
AW200398		3
AW637088		3
AW641055	PCTK2	3
AW646316		3
AW764914		3
AW782935		3
AY164461	SFRP5	3
AY648303		3
AY731504		3
BC041213	CA2	3
BC041222	RP2	3
BC041315	CPA3	3
BC041753	CFI	3
BC043624	PPP2R1A	3
BC043781	PGK1	3
BC043819	IRAK2	3

BC043879	ZNF622	3
BC043994		3
BC044007	PKM2	3
BC044093	PRPF19	3
BC044326	NEK6	3
BC044982	SLIT1	3
BC045131	B4GALNT1	3
BC046378	HYAL2	3
BC047247	KCNK6	3
BC047972	WDR36	3
BC048226	C17orf40	3
BC054177	NPM1	3
BC054222	CYP46A1	3
BC054253	FAM82B	3
BC054957	CD63	3
BC055988	SRFBP1	3
BC056029	TEKT3	3
BC056128	WNT5B	3
BC056840	LTF	3
BC056847	CYP2C8	3
BC059306	SLC45A3	3
BC060473	SLCO2A1	3
BC061650	TAGLN	3
BC061655	PLS3	3
BC068692	C14orf111	3
BC068695	WARS	3
BC068748		3
BC068757	GLTP	3
BC068845	NOLA2	3
BC068940	CDH1	3
BC070652	PQLC1	3
BC070683		3
BC070698	PUS7L	3
BC070738	CENTG2	3
BC072067	PMPCB	3
BC072199	SRPK1	3
BC072214	DDX47	3
BC072223	BYSL	3
BC072808	ARID3A	3
BC072842	TRIM2	3
BC072927	PIPOX	3
BC072976		3
BC072989		3
BC073227	TSEN2	3
BC073384	ASMTL	3
BC073430	NMD3	3
BC073463	DNAI1	3
BC073536	NFATC1	3
BC073578		3

BC073612	PPP2R1A	3
BC073712		3
BC074222	SLC16A12	3
BC074251	GPX3	3
BC074463	FCN2	3
BC074496	PRAF1	3
BC075175	SCRIB	3
BC076784		3
BC076838	CRY1	3
BC077065		3
BC077206	PDCL3	3
BC077415	EXOSC7	3
BC077582	TLE1	3
BC077606	PPOX	3
BC077638	BXDC5	3
BC077799	MED8	3
BC077836	UAP1	3
BC077990	SLC38A2	3
BC078028	C1orf93	3
BC078106	ZFYVE28	3
BC079784	TMEM48	3
BC079982		3
BC080095	C3orf26	3
BC081042		3
BC081074		3
BC081085	AGPAT1	3
BC081130	C1orf139	3
BC081246	LRRC22	3
BC082380	PPID	3
BC082444		3
BC082530		3
BC082624	NCDN	3
BC082640		3
BC082652		3
BC082875	RBM19	3
BC082912	CFD	3
BC082934	CYP3A4	3
BC083003	ADH1B	3
BC083035		3
BC084066	CDC2L1	3
BC084278	NOC3L	3
BC084657		3
BC084838	GSTO1	3
BC084984	CEBPZ	3
BC085021	PDLIM2	3
BC085055	ANPEP	3
BC086270		3
BC086273		3
BC087309		3

BC088708	NUBP1	3
BC089147	B3GNT3	3
BC090160	C14orf32	3
BC090198		3
BC092020		3
BC097539	CDC42SE2	3
BC097649	WDR51B	3
BC098959	PREP	3
BC099298		3
BE679568		3
BF610767		3
BF615462	RABL5	3
BG037998		3
BG162399		3
BG162682		3
BG234364		3
BG811067		3
BJ029555		3
BJ057663	AMID	3
BJ067458		3
BJ068336	NEK6	3
BJ070078		3
BJ089329	MACF1	3
BJ616292		3
BJ633113		3
BJ635358		3
BM179237	SYN3	3
BP685770		3
BP686897		3
BP701638		3
BP707684	CAPN2	3
BP734641		3
BP742091		3
BQ398814	ZNF365	3
BQ725721		3
BQ726537	MUC5B	3
BQ726603		3
BQ733925		3
BX843674	ABO	3
BX843801	SMTN	3
BX844453	DIO1	3
BX845905	DOM3Z	3
BX846124		3
BX847425		3
BX849962	PTPRR	3
BX852494	C2orf17	3
BX855102	C5orf15	3
BX855204		3
CA972804	E2F5	3

CB561838		3
CB756263	PARP14	3
CD302763	MLH1	3
CD328604		3
CD363147	USP8	3
CF284278		3
CF288050	C6orf85	3
CK742103		3
CK797414		3
CK798523		3
CK798738		3
CK799950		3
CK800173		3
CK800700		3
CK805115		3
CK805195		3
CK815991	FLJ20397	3
CV113365		3
CX130840		3
D86492	IRF6	3
DQ096860		3
DQ096919		3
L20816	PLCB3	3
U45453	GATA4	3
U65897	ZNF585A	3
X88927	NOLC1	3
Z14253	ETF1	3
AB030502	UBQLN4	4
AB034701	COL1A1	4
AB036753	PORCN	4
AB070723		4
AB080020	LGALS3	4
AF033110	FZD5	4
AF139914	HES1	4
AF185580	HLA-B	4
AF197945	PTPRF	4
AF207901	CGN	4
AF274053	KCNMA1	4
AF314056	AGR2	4
AF318178	GCGR	4
AF319538	EXT1	4
AF353715	KLF4	4
AF364821	MYL4	4
AF427862	PECAM1	4
AF432354	P2RY1	4
AF508961	LRP6	4
AF513854	DIRAS1	4
AF529219		4
AF533513	KHSRP	4

AJ243591	PCBP2	4
AJ278065	AGXT	4
AJ344435	FOXD2	4
AJ555187		4
AW199587		4
AW199686		4
AW200120		4
AW200470	EMP2	4
AW200505	VAPB	4
AW200509		4
AW633756		4
AW634233	ATF1	4
AW635861		4
AW635923		4
AW644252		4
AW645021		4
AW765923		4
AW782309		4
AW871732		4
AY152672	SLC26A6	4
AY260728	SLC5A8	4
AY278679	FUT3	4
AY340103	PER2	4
AY352638	MTUS1	4
AY573378	MMP15	4
AY689185		4
AY714078	RUNX1T1	4
AY901984	FURIN	4
BC041316	ENTPD2	4
BC041751	C15orf44	4
BC042252		4
BC042275	EGFL6	4
BC042357	LCAT	4
BC042927	KRT12	4
BC043737		4
BC043782	INA	4
BC043786	SLC37A1	4
BC043787	PHF12	4
BC043788	OGN	4
BC043850	ACSBG2	4
BC043876	PGM1	4
BC044080	ALDH9A1	4
BC044086	ZFP36L2	4
BC044088	CNOT4	4
BC044682	UCP2	4
BC044968	EMP2	4
BC045013	SPARC	4
BC045022	ID4	4
BC045030	DYNC1LI1	4

BC045083	EEF1A1	4
BC045132	LMBR1L	4
BC045218	ALDOC	4
BC045220	MATN2	4
BC045265	CYB5R3	4
BC046653	ECE1	4
BC046670	PTRF	4
BC046701	FAM62B	4
BC046726	SAMM50	4
BC047259	C22orf9	4
BC047982	UBQLN4	4
BC048021	GCNT1	4
BC048224	GRN	4
BC049008	WASF2	4
BC049177	ANKRD25	4
BC051009	BAT2	4
BC054202	CYP2C8	4
BC054247	CYP8B1	4
BC054248	FTCD	4
BC054260	ADH1B	4
BC054282	LUM	4
BC054284	HSD11B1	4
BC054997	EFNA3	4
BC055970		4
BC055989	BACE2	4
BC056005	UBE2R2	4
BC056012	FAM3D	4
BC056063	FNBP4	4
BC056091	GK	4
BC056125	PDCD4	4
BC056838	CYR61	4
BC056849	CTRB1	4
BC056856	CPA1	4
BC057715	PCK1	4
BC057717	SEMA3F	4
BC057722	KIAA1914	4
BC057735	LOH11CR2A	4
BC057753	RNF121	4
BC059294	A2M	4
BC059295	TFCP2	4
BC059305	DCTN1	4
BC059786	APOA1	4
BC059963	BACE2	4
BC059986	SNX22	4
BC060015	RAB10	4
BC060328	CYP2C8	4
BC060361	TKT	4
BC060364	ABAT	4
BC060367	EXT2	4

BC060417	ATP6V0A4	4
BC060465	HSD17B6	4
BC060497	HSD17B6	4
BC060499	SLC30A1	4
BC060745	EPHA2	4
BC060751	SLC7A5	4
BC061663	HAAO	4
BC061680	RDH16	4
BC063725	CRSP3	4
BC063731	HS3ST1	4
BC063735	GNAS	4
BC066120		4
BC068660	TSG101	4
BC068683		4
BC068772		4
BC068779	RFX5	4
BC068817	ZCCHC9	4
BC068927		4
BC068964	RAG1AP1	4
BC070531	UCP2	4
BC070558	ITGB1	4
BC070603	RNF111	4
BC070620	CDC42EP4	4
BC070669	ADH1B	4
BC070672	CAV1	4
BC070682	KRT19	4
BC070735		4
BC070750	DDEF2	4
BC070759		4
BC070774	TMCC1	4
BC070794		4
BC070813	BCL9	4
BC070826	ANKRD13C	4
BC071004	SULT2B1	4
BC071021	CYR61	4
BC071033	EPHX1	4
BC071039	NIT2	4
BC071040		4
BC071055	PDLIM7	4
BC071114		4
BC072092	AQP1	4
BC072097	ACTC	4
BC072100		4
BC072154		4
BC072158	RAB27B	4
BC072163	PYGB	4
BC072168	CEBPA	4
BC072179	PDLIM7	4
BC072269	PDGFRL	4

BC072274		4
BC072308	SLC25A11	4
BC072765	GPM6B	4
BC072821		4
BC072890	ANXA3	4
BC072901		4
BC072912	SSTR2	4
BC072970	ELA1	4
BC072978	PRSS3	4
BC072986	HSD3B7	4
BC073030	HADHA	4
BC073056	EDF1	4
BC073092		4
BC073249		4
BC073282	L1CAM	4
BC073285	RARRES1	4
BC073295		4
BC073298	OGDH	4
BC073316	TRIB1	4
BC073343	ITGB3	4
BC073362	TMEM39A	4
BC073444		4
BC073576	RSU1	4
BC073583	EAF1	4
BC073622		4
BC073624	NT5DC3	4
BC073634		4
BC073649	ILK	4
BC073695	KLF11	4
BC073706	PIK4CB	4
BC073716		4
BC074103	DVL1	4
BC074113		4
BC074135		4
BC074144		4
BC074146	HAL	4
BC074149	CYP2J2	4
BC074179	ABAT	4
BC074196	ACOT12	4
BC074204	CYP2A13	4
BC074253	STAU1	4
BC074283		4
BC074300		4
BC074306	HINT3	4
BC074340		4
BC074344	RAB25	4
BC074358	PDE6H	4
BC074399		4
BC074415		4

BC075176		4
BC076643		4
BC076777		4
BC076786	BRD4	4
BC076806	HSPB7	4
BC076834	COQ10B	4
BC077183	SNX9	4
BC077219	AGXT2L1	4
BC077245	RAB27B	4
BC077255	MAFB	4
BC077275	RGS5	4
BC077281	PRKACA	4
BC077308		4
BC077330		4
BC077332	B3GNT5	4
BC077372		4
BC077430	FBXL14	4
BC077501	ARRDC1	4
BC077535		4
BC077551	ARNTL	4
BC077568		4
BC077573		4
BC077584	SUOX	4
BC077639		4
BC077745	PPFIBP1	4
BC077785	XPR1	4
BC077819		4
BC077820	FBXW5	4
BC077821		4
BC077850	CYP2A13	4
BC077859		4
BC077880	PMP22	4
BC077881	APBB2	4
BC077886		4
BC077891	ITGA2B	4
BC077900	MYL9	4
BC077910	GLUD1	4
BC077911		4
BC077965	GPD1	4
BC077991	RAD21	4
BC078019	PRKCD	4
BC078021		4
BC078056	SELENBP1	4
BC078061		4
BC078079	RHBG	4
BC078559		4
BC078565		4
BC078615		4
BC079703		4

BC079728	NECAP2	4
BC079769		4
BC079798	QSCN6L1	4
BC079799	TLE4	4
BC079803	ELL	4
BC079804	PNPLA2	4
BC079815		4
BC079822	NUFIP2	4
BC079920		4
BC080009		4
BC080025	ADSSL1	4
BC080096	SULT1A1	4
BC080118	ADIPOR2	4
BC080167	CAMK2D	4
BC080378	SLC25A29	4
BC080428		4
BC081023	EDG4	4
BC081121	INPP5A	4
BC081123		4
BC081126	GNAQ	4
BC081152	NFIX	4
BC081153	RBPMS2	4
BC081162	SERPINB4	4
BC081170		4
BC081179		4
BC081228		4
BC081239	CSRP1	4
BC081272		4
BC081275	PLS3	4
BC082396	CYP2C8	4
BC082401	DPP4	4
BC082452		4
BC082460	CPLX1	4
BC082462	CAPNS1	4
BC082467		4
BC082612	SLC37A3	4
BC082614	POPDC2	4
BC082642	RAB14	4
BC082647		4
BC082680		4
BC082713	CDC25C	4
BC082817	MYADM	4
BC082856	AXUD1	4
BC082896		4
BC082904		4
BC082923	TNNI3	4
BC082952	PYGL	4
BC083037	GRHPR	4
BC083041		4

BC084074		4
BC084215		4
BC084221	ZFP36L2	4
BC084263	GNB1	4
BC084270		4
BC084294		4
BC084318	ATP6V1G3	4
BC084347	PDE4B	4
BC084388		4
BC084398	GPR172A	4
BC084404	CSRP3	4
BC084428		4
BC084644	HLA-DQA2	4
BC084743	KRAS	4
BC084778		4
BC084848	TAGLN	4
BC084860	ME3	4
BC084887		4
BC084889		4
BC084930	CAMK1	4
BC084953	JAG1	4
BC084962	ATP2A1	4
BC084969	SSPN	4
BC085026		4
BC085039	CRIP1	4
BC085069	TPM3	4
BC085209	FAM55A	4
BC086282	CREBBP	4
BC087382		4
BC087410		4
BC087417		4
BC087465	SNTA1	4
BC087549		4
BC087736		4
BC088668	OACT5	4
BC088670	LOXL1	4
BC088676		4
BC088904		4
BC088932	PPFIA1	4
BC088939	TPM3	4
BC089117	PHACTR4	4
BC090197	RFC1	4
BC091628		4
BC092027		4
BC092031		4
BC092148	CASKIN2	4
BC092317	CREB3	4
BC094169	SHANK2	4
BC097856	FBS1	4

BC098967	ADD3	4
BE132270		4
BE505183		4
BE505195		4
BE505753		4
BE507632		4
BE509211		4
BE509325		4
BE576052		4
BE679575		4
BF024854		4
BF025071	ALOX15B	4
BF025586		4
BF048582		4
BF071806		4
BF072240		4
BF231908		4
BF232175	ZNF21	4
BF232367		4
BF428025		4
BF611992		4
BF612748		4
BF615296		4
BF615503		4
BG016213	CDX2	4
BG018949		4
BG019755	PYCR2	4
BG022166		4
BG023235		4
BG264249	CHD3	4
BG346671		4
BG346716		4
BG409953		4
BG486800		4
BG513502	ME2	4
BG513927		4
BG553365		4
BG811389		4
BG811769		4
BI312922		4
BJ033261		4
BJ036670		4
BJ050558		4
BJ056228		4
BJ069449	MSRB2	4
BJ070981		4
BJ079142	FKBP10	4
BJ080002		4
BJ081305		4

BJ083221		4
BJ092482	RHBDL7	4
BJ612247		4
BJ623141		4
BJ625984	ABCB4	4
BJ632440		4
BM180994	RAB15	4
BM191646	SLC2A9	4
BP686117		4
BP687284		4
BP691429		4
BP694519	LARGE	4
BP695201		4
BP695549		4
BP696309		4
BP704578		4
BP705076		4
BP705466		4
BP708037		4
BP708540		4
BP713293		4
BP713744		4
BP732418		4
BQ387543		4
BQ388127		4
BQ723797	B4GALT1	4
BQ729848	UCHL1	4
BQ731271		4
BQ732824	TAF4	4
BQ732928	AUP1	4
BQ734261		4
BQ735063		4
BQ735436		4
BQ735985		4
BQ737487		4
BX843298	PAFAH1B1	4
BX843353	RALGDS	4
BX844492		4
BX844777	CASR	4
BX845304		4
BX845609	PRSS2	4
BX846064	PGLYRP1	4
BX846071	CA12	4
BX846116	MUC3B	4
BX846205		4
BX846582	TNXB	4
BX847250		4
BX847353	S100A14	4
BX847376		4

BX847508	COX17	4
BX848085		4
BX848168		4
BX848252	RASEF	4
BX849057		4
BX849222		4
BX849304	ELA1	4
BX849457		4
BX849887	CRP	4
BX850233		4
BX851355		4
BX851706		4
BX851993		4
BX852516	FAM55A	4
BX852552		4
BX853192		4
BX853259		4
BX853459	HAGH	4
BX853583		4
BX853798		4
BX854001	L2HGDH	4
CA973085		4
CB559907		4
CB942111		4
CB942633		4
CB943692	CRISP3	4
CB944297		4
CD099362	LMAN1	4
CD099720		4
CD099943		4
CD253705		4
CD255791		4
CD256700		4
CD300904		4
CD301636		4
CD302358	COL6A3	4
CD303416		4
CD326941	PTGS2	4
CD327200		4
CD328101		4
CD330023		4
CD330078	NUBPL	4
CF271248	LGALS1	4
CF271359	SON	4
CF271543	SPINK1	4
CF284196		4
CF284526		4
CF285062		4
CF285110		4

CF285386	AKAP11	4
CF285661		4
CF285662		4
CF286636	C6orf108	4
CF519906		4
CF521081		4
CF521166		4
CF548303		4
CF548399		4
CK796877		4
CK797077	PRDX5	4
CK797112		4
CK797196		4
CK797424		4
CK797591		4
CK798105		4
CK798161	SOD1	4
CK798619		4
CK798638		4
CK799264	CST3	4
CK800512		4
CK805290	TRUB2	4
CO382862		4
CO384445	C9orf10	4
CV077028		4
CV077651		4
CV079516	SPINK2	4
CX131041		4
CX131946		4
D13688	HLA-DQB1	4
DR717081		4
L11446	RXRA	4
L28111	DIO3	4
M23237	PDGFA	4
M29857	IGF1	4
M33874	NOTCH1	4
M88105	LGALS1	4
U09135	PTPRA	4
U29448	NODAL	4
U47622	FGF9	4
U63817	TRIM14	4
U67129	AR	4
U88065	ADAR	4
X87365	RARA	4
X90838	SLC8A1	4
Z19541	VCL	4
Z47557	CCK	4
AB022088	CYP1A1	5
AB037936	RGN	5

AB075925	OLFM4	5
AF036617	FECH	5
AF193799	GHR	5
AF358869	WEE1	5
AF388036	BOC	5
AF527799	BVES	5
AW200257	PEMT	5
AW200624		5
AY949838	PELP1	5
BC042228	FZD7	5
BC042288	CDC20	5
BC043748		5
BC044048	UTP15	5
BC044112	PDK2	5
BC044995	DHCR7	5
BC046856	EIF4A2	5
BC046945	BXDC1	5
BC054152	SQRDL	5
BC054153	GLUL	5
BC054181	KDELR3	5
BC054283	FAH	5
BC056038	DSCR1	5
BC056121	FARSLB	5
BC056845	ACAD8	5
BC057757	MARS	5
BC060331	G6PC	5
BC068714	MAD2L1	5
BC068729	RBM7	5
BC068878		5
BC068895	KIAA0859	5
BC068907	DDX18	5
BC068968	MASTL	5
BC070595	C15orf41	5
BC070608		5
BC070692	SRM	5
BC071096	METT10D	5
BC071128	CBR1	5
BC071140	MTX1	5
BC072192	IKBKB	5
BC072276	KPTN	5
BC072331		5
BC072360	RAB30	5
BC072380	POLR3D	5
BC073000		5
BC073014	BXDC2	5
BC073032	EEFSEC	5
BC073058	SMYD5	5
BC073297		5
BC073507		5

BC073561	SEH1L	5
BC073613	F7	5
BC073632		5
BC074191	GCHFR	5
BC074234		5
BC074377	JUN	5
BC074488		5
BC076771	FRAT1	5
BC076789	CHRNA5	5
BC077216	PHB2	5
BC077417	F11	5
BC077458	SF3B4	5
BC077469	FAM98B	5
BC077523		5
BC077826		5
BC077929	AOC3	5
BC078464	RBM13	5
BC079680	SHMT2	5
BC079713	LARS	5
BC079735		5
BC080437		5
BC081034	ABCF1	5
BC081192	MRPL2	5
BC082400	USP14	5
BC082480	TATDN1	5
BC082481		5
BC082696		5
BC082839		5
BC082884	C10orf22	5
BC082900		5
BC084071	LAMB2	5
BC084265	HMBS	5
BC084783	CIDEA	5
BC084828	MRPL12	5
BC084949	TIMM22	5
BC084973		5
BC086477	IRS2	5
BC087343	PSMD10	5
BC087399	KCTD18	5
BC087450		5
BC092018		5
BF614713		5
BG016864		5
BI314985		5
BI940841		5
BJ040605		5
BJ055092		5
BJ055719		5
BJ063969	PDLIM3	5

BJ068483		5
BJ075553	NOL6	5
BJ624645		5
BJ643315	CRBN	5
BM190859		5
BM192842		5
BP684433		5
BP690860		5
BP691809		5
BP698942	MRPS23	5
BP705037		5
BP706536		5
BP709301		5
BQ724783		5
BQ736389	SMN1	5
BX844221		5
BX844946		5
BX852510		5
BX852927	SEC11L3	5
CA982484		5
CB756739	PHYHIPL	5
CD253113		5
CD255149		5
CD256625		5
CF286689		5
CF519799		5
CF549269		5
CK798087		5
CK799531		5
CK800041		5
CV073503		5
CV076731		5
DR728320		5
X53962	CDK7	5
X69662	GSS	5
X74315	FOXI1	5
Z95080	HAND1	5
AF061727	SERPIND1	6
AF062387	ABCB9	6
AF440822	PTPNS1	6
AF465789	TLL1	6
AW164985		6
AW199362	CLK2	6
AW200023	PLK3	6
AW200581		6
AY035397	SOX9	6
AY204551	TAP1	6
AY363297		6
AY504996	C20orf32	6

BC041721	CAMK1	6
BC043783	YES1	6
BC044008	MST1	6
BC044717	STAT3	6
BC046837	P2RY4	6
BC047965	KARS	6
BC048225	PDCD4	6
BC049174	SLC2A3	6
BC054188	PPP1CC	6
BC054206	OGFR	6
BC054214	SOCS3	6
BC054227	CEL	6
BC054232	SERPINB6	6
BC054271	CEL	6
BC054295	TSPAN8	6
BC056025	NR4A1	6
BC056033	SLC25A24	6
BC056035	SLA2	6
BC056039	PSMB7	6
BC056111	CMTM7	6
BC056841	AMY2A	6
BC059315	GBP7	6
BC059335	PSMB8	6
BC059337	FAM49A	6
BC059976	CD74	6
BC059984	IRF1	6
BC060023	NDFIP2	6
BC060360	PNLIPRP2	6
BC060433	DEF6	6
BC061649	SNX5	6
BC061672	STEAP2	6
BC061681	HLA-DMA	6
BC068616	RAB24	6
BC068656	RCBTB1	6
BC068752	SOCS3	6
BC068787	JUP	6
BC068851	WBP11	6
BC070713	TRAF2	6
BC070719	VPS24	6
BC070796	TMEM30B	6
BC070857	SLC36A1	6
BC071132	CD9	6
BC072796	CASP6	6
BC072847	SOCS1	6
BC072916	RNPEP	6
BC072984	IRF1	6
BC073340	PSME1	6
BC073351		6
BC073363	PPT2	6

BC073402		6
BC073417	CENTB1	6
BC073419	STARD3	6
BC073478	FAM102A	6
BC073525		6
BC073546		6
BC073555	CTRB1	6
BC073582		6
BC073588	ZNFN1A3	6
BC073647	SERPINF1	6
BC073669		6
BC073694	GPD2	6
BC074200	HAO2	6
BC074357	PERP	6
BC075169		6
BC075237	C20orf100	6
BC077187	IRF2	6
BC077193	TXNIP	6
BC077231	STX11	6
BC077292	COL9A3	6
BC077420		6
BC077485	ATP1B3	6
BC077564		6
BC077840	RHOV	6
BC077868		6
BC077883		6
BC077896		6
BC077916	SOAT1	6
BC077941		6
BC077994		6
BC078585		6
BC079707		6
BC079773	C1R	6
BC079813		6
BC080035		6
BC080036		6
BC080063	RFFL	6
BC081091	GBP6	6
BC081221	PSTPIP1	6
BC082848		6
BC082930		6
BC082936		6
BC083025	PLEC1	6
BC083045		6
BC084073		6
BC084236	MARCH8	6
BC084374	BTN2A1	6
BC084606	RAB37	6
BC084607		6

BC084633	TRIM25	6
BC084666		6
BC084831	CPA1	6
BC084832		6
BC084840	ST3GAL6	6
BC084896		6
BC084974	C1S	6
BC085029		6
BC085042		6
BC085061		6
BC085064		6
BC085212	HLA-A	6
BC087297		6
BC088746		6
BC089148	PRSS16	6
BC089175	MR1	6
BC090219	TMBIM4	6
BC092033	GPA33	6
BC092343		6
BC097538	PTPN6	6
BC099017		6
BE192039		6
BE507817	BTN3A1	6
BE508034		6
BE508394		6
BE669296		6
BF047119		6
BF047391		6
BF072051		6
BF427961		6
BF428058	KARS	6
BG017658	PSD4	6
BG018164		6
BG019050	PYCARD	6
BG020384	C17orf37	6
BG020825		6
BG023176		6
BG037463		6
BG160621		6
BG161028		6
BG161758		6
BG232841	CRBN	6
BG233711		6
BG234189	ARHGAP27	6
BG346398	IREB2	6
BG360221		6
BG407150		6
BG438924	PTK2	6
BI312781	ADAMTS13	6

BI446136	ARHGEF16	6
BJ042255		6
BJ045539		6
BJ083741	NHSL1	6
BJ085940		6
BJ089284		6
BJ090358		6
BJ092595		6
BJ094364		6
BJ633168	SLC30A5	6
BJ634431		6
BM191386		6
BM261196	IL2RG	6
BM262453		6
BP683892		6
BP687254		6
BP687360		6
BP688269	SUB1	6
BP688919		6
BP691572		6
BP699528		6
BP700722		6
BP707937		6
BP741772		6
BQ384402		6
BQ385449		6
BQ723998		6
BQ733387	GRM2	6
BX842732		6
BX845117	P2RY5	6
BX845723		6
BX846343	SEPT4	6
BX847233		6
BX850413		6
BX850436		6
BX850727		6
BX851469		6
BX852027	CCL5	6
BX853374		6
BX854078	BCL11B	6
BX854219	DNAJB1	6
CA982195		6
CB560728		6
CB561644	NOMO2	6
CB563723		6
CB941299	CCL13	6
CB943125		6
CD301800		6
CD326347	LENG8	6

CD328147		6
CD328776		6
CD361010		6
CD361037		6
CF270416		6
CF271104	TOR1A	6
CF284976		6
CF285027		6
CF520127		6
CK797290		6
CK800094		6
CK800212		6
CX131348	PSMB9	6
CX135390		6
D13684	HLA-DQB1	6
D44540	PSMB8	6
L20725	MR1	6
L25857	HOXD1	6
M58576	ANXA2	6
Y12326	CD3G	6

Supplemental Table S6-1: Cluster 1 associated GO Categories. Statistically significant GO categories ($p < 0.05$ as determined by the GoMiner software).

GO ID	Total	Change	P-Value (Changed)	Term
65007	2672	433	0.00	biological regulation
50789	2408	393	0.00	regulation of biological process
16020	2401	391	0.00	membrane
50794	2235	369	0.00	regulation of cellular process
32501	1945	364	0.00	multicellular organismal process
32502	2012	344	0.00	developmental process
44425	1918	323	0.00	membrane part
7275	1590	302	0.00	multicellular organismal development
7154	1649	301	0.00	cell communication
48856	1497	274	0.00	anatomical structure development
7165	1482	271	0.00	signal transduction
31224	1519	261	0.00	intrinsic to membrane
48731	1316	256	0.00	system development
16021	1492	256	0.00	integral to membrane
5886	1120	233	0.00	plasma membrane
48869	1269	222	0.00	cellular developmental process
30154	1268	222	0.00	cell differentiation
50896	1346	222	0.01	response to stimulus
48513	1071	211	0.00	organ development
9653	923	184	0.00	anatomical structure morphogenesis
48519	914	183	0.00	negative regulation of biological process
5576	876	175	0.00	extracellular region
48523	864	171	0.00	negative regulation of cellular process
48518	843	168	0.00	positive regulation of biological process
48468	981	163	0.02	cell development
44459	757	159	0.00	plasma membrane part
48522	772	154	0.00	positive regulation of cellular process
44421	742	147	0.00	extracellular region part
7166	674	145	0.00	cell surface receptor linked signal transduction

60089	742	143	0.00	molecular transducer activity
4871	742	143	0.00	signal transducer activity
5615	684	142	0.00	extracellular space
7242	816	133	0.05	intracellular signaling cascade
65008	728	130	0.00	regulation of biological quality
7399	590	129	0.00	nervous system development
30528	766	129	0.02	transcription regulator activity
6950	772	127	0.05	response to stress
8283	551	122	0.00	cell proliferation
50793	677	119	0.01	regulation of developmental process
9605	453	111	0.00	response to external stimulus
9887	491	109	0.00	organ morphogenesis
3008	596	103	0.02	system process
4872	528	101	0.00	receptor activity
6366	494	96	0.00	transcription from RNA polymerase II promoter
31226	447	94	0.00	intrinsic to plasma membrane
42127	345	93	0.00	regulation of cell proliferation
2376	396	93	0.00	immune system process
5887	443	93	0.00	integral to plasma membrane
3700	490	92	0.00	transcription factor activity
22610	345	91	0.00	biological adhesion
7155	345	91	0.00	cell adhesion
9790	428	85	0.00	embryonic development
51674	385	84	0.00	localization of cell
6928	385	84	0.00	cell motility
6357	384	79	0.00	regulation of transcription from RNA polymerase II promoter
9611	252	73	0.00	response to wounding
5509	352	73	0.00	calcium ion binding
9966	394	72	0.02	regulation of signal transduction
16477	284	71	0.00	cell migration
32989	364	71	0.00	cellular structure morphogenesis
902	364	71	0.00	cell morphogenesis
9893	368	71	0.00	positive regulation of metabolic process
31325	360	70	0.00	positive regulation of cellular metabolic process
31324	374	70	0.01	negative regulation of cellular metabolic process
9892	380	70	0.01	negative regulation of metabolic process

22008	327	66	0.00	neurogenesis
65009	367	66	0.03	regulation of molecular function
9888	278	65	0.00	tissue development
4888	289	65	0.00	transmembrane receptor activity
7267	337	65	0.01	cell-cell signaling
51093	312	61	0.01	negative regulation of developmental process
42592	345	61	0.04	homeostatic process
48699	306	60	0.01	generation of neurons
42995	320	60	0.02	cell projection
7417	250	58	0.00	central nervous system development
6952	230	57	0.00	defense response
51094	300	57	0.01	positive regulation of developmental process
40007	291	56	0.01	growth
48646	202	55	0.00	anatomical structure formation
7389	237	55	0.00	pattern specification process
30182	270	55	0.00	neuron differentiation
7186	215	54	0.00	G-protein coupled receptor protein signaling pathway
6955	222	54	0.00	immune response
48858	261	53	0.00	cell projection morphogenesis
32990	261	53	0.00	cell part morphogenesis
30030	261	53	0.00	cell projection organization and biogenesis
45941	268	53	0.01	positive regulation of transcription
45935	278	53	0.02	positive regulation of nucleobase, nucleoside, nucleotide and nucleic acid metabolic process
45934	283	53	0.02	negative regulation of nucleobase, nucleoside, nucleotide and nucleic acid metabolic process
7167	227	51	0.00	enzyme linked receptor protein signaling pathway
16481	246	51	0.00	negative regulation of transcription
5667	260	51	0.01	transcription factor complex

Supplemental Table S6-2: Cluster 2 associated GO Categories. Statistically significant GO categories ($p < 0.05$ as determined by the GoMiner software).

GO ID	Total	Change	P-Value (Changed)	Term
44464	5453	518	0.00	cell part
5623	5454	518	0.00	cell
5622	4687	459	0.00	intracellular
44424	4576	450	0.00	intracellular part
5737	3384	404	0.00	cytoplasm
43229	3913	391	0.00	intracellular organelle
43226	3920	391	0.00	organelle
8152	3839	374	0.01	metabolic process
43231	3593	361	0.00	intracellular membrane-bounded organelle
43227	3596	361	0.00	membrane-bounded organelle
3824	2550	330	0.00	catalytic activity
44444	2324	325	0.00	cytoplasmic part
16020	2401	289	0.00	membrane
44422	2226	238	0.00	organelle part
44425	1918	235	0.00	membrane part
44446	2214	235	0.00	intracellular organelle part
51179	1889	204	0.00	localization
51234	1600	183	0.00	establishment of localization
6810	1526	179	0.00	transport
31224	1519	176	0.00	intrinsic to membrane
16021	1492	173	0.00	integral to membrane
43167	1593	167	0.02	ion binding
46872	1565	164	0.02	metal ion binding
31090	835	157	0.00	organelle membrane
5739	650	152	0.00	mitochondrion
9058	882	142	0.00	biosynthetic process
16491	397	117	0.00	oxidoreductase activity
16740	936	113	0.00	transferase activity
44429	396	104	0.00	mitochondrial part
44249	676	102	0.00	cellular biosynthetic process
31967	382	96	0.00	organelle envelope
31975	386	96	0.00	envelope
6629	394	96	0.00	lipid metabolic process

267	594	90	0.00	cell fraction
44255	338	83	0.00	cellular lipid metabolic process
6082	355	82	0.00	organic acid metabolic process
5740	266	81	0.00	mitochondrial envelope
19752	354	81	0.00	carboxylic acid metabolic process
31966	249	79	0.00	mitochondrial membrane
12505	564	79	0.00	endomembrane system
9056	506	78	0.00	catabolic process
5783	469	77	0.00	endoplasmic reticulum
42221	560	76	0.00	response to chemical stimulus
44248	431	74	0.00	cellular catabolic process
19866	217	72	0.00	organelle inner membrane
5215	594	72	0.01	transporter activity
5743	201	68	0.00	mitochondrial inner membrane
6796	586	65	0.05	phosphate metabolic process
6793	586	65	0.05	phosphorus metabolic process
22892	482	63	0.00	substrate-specific transporter activity
22857	414	62	0.00	transmembrane transporter activity
6811	508	62	0.01	ion transport
5624	422	61	0.00	membrane fraction
16310	504	59	0.02	phosphorylation
22891	373	55	0.00	substrate-specific transmembrane transporter activity
6091	189	54	0.00	generation of precursor metabolites and energy
6812	418	54	0.00	cation transport
6066	201	53	0.00	alcohol metabolic process
42175	256	50	0.00	nuclear envelope-endoplasmic reticulum network
44432	274	50	0.00	endoplasmic reticulum part

Supplemental Table S6-3: Cluster 3 associated GO Categories. Statistically significant GO categories ($p < 0.05$ as determined by the GoMiner software).

GO ID	Total	Change	P-Value (Changed)	Term
5737	2739	62	0.02	cytoplasm
3824	2092	49	0.02	catalytic activity
46872	1378	33	0.05	metal ion binding
43169	1252	31	0.04	cation binding
16740	701	19	0.05	transferase activity
6396	221	12	0.00	RNA processing
267	352	12	0.03	cell fraction
8233	215	9	0.02	peptidase activity
42254	49	8	0.00	ribosome biogenesis and assembly
22613	101	8	0.00	ribonucleoprotein complex biogenesis and assembly
5730	80	7	0.00	nucleolus
4175	147	7	0.02	endopeptidase activity
16072	33	5	0.00	rRNA metabolic process

Supplemental Table S6-4: Cluster 4 associated GO Categories. Statistically significant GO categories ($p < 0.05$ as determined by the GoMiner software).

GO ID	Total	Change	P-Value (Changed)	Term
16020	2018	126	0.00	membrane
44425	1616	96	0.02	membrane part
32501	1217	88	0.00	multicellular organismal process
31224	1308	82	0.01	intrinsic to membrane
16021	1285	81	0.01	integral to membrane
7154	1244	80	0.01	cell communication
5886	869	64	0.00	plasma membrane
7275	886	58	0.02	multicellular organismal development
48856	771	50	0.03	anatomical structure development
48731	664	46	0.01	system development
5576	477	44	0.00	extracellular region
44459	580	38	0.05	plasma membrane part
48513	483	35	0.01	organ development
48519	515	34	0.05	negative regulation of biological process
3008	374	32	0.00	system process
65008	383	31	0.00	regulation of biological quality
7166	463	31	0.05	cell surface receptor linked signal transduction
16491	318	30	0.00	oxidoreductase activity
5509	301	27	0.00	calcium ion binding
8283	323	27	0.01	cell proliferation
4872	360	27	0.02	receptor activity
5783	366	27	0.03	endoplasmic reticulum
6629	287	25	0.00	lipid metabolic process
44421	254	24	0.00	extracellular region part
19752	236	23	0.00	carboxylic acid metabolic process
6082	238	23	0.00	organic acid metabolic process
42221	213	22	0.00	response to chemical stimulus
5794	286	22	0.03	Golgi apparatus
42127	193	20	0.00	regulation of cell proliferation
9605	230	20	0.01	response to external stimulus
44255	237	20	0.01	cellular lipid metabolic process
22610	246	19	0.04	biological adhesion
7155	246	19	0.04	cell adhesion
7267	187	18	0.01	cell-cell signaling
32787	119	16	0.00	monocarboxylic acid metabolic process
46983	150	15	0.01	protein dimerization activity

19001	172	15	0.02	guanyl nucleotide binding
32561	172	15	0.02	guanyl ribonucleotide binding
6807	172	15	0.02	nitrogen compound metabolic process
6928	187	15	0.05	cell motility
51674	187	15	0.05	localization of cell
9308	160	14	0.03	amine metabolic process
44431	165	14	0.04	Golgi apparatus part
5525	167	14	0.04	GTP binding
7517	86	13	0.00	muscle development
31012	115	13	0.00	extracellular matrix
6066	133	13	0.01	alcohol metabolic process
46903	134	13	0.02	secretion
5615	155	13	0.05	extracellular space
48037	106	12	0.01	cofactor binding
6519	136	12	0.04	amino acid and derivative metabolic process
51239	83	11	0.00	regulation of multicellular organismal process
8284	90	11	0.00	positive regulation of cell proliferation
5578	113	11	0.02	proteinaceous extracellular matrix
8610	118	11	0.03	lipid biosynthetic process
40007	119	11	0.03	growth
16614	54	10	0.00	oxidoreductase activity, acting on CH-OH group of donors
5792	72	10	0.00	microsome
45595	72	10	0.00	regulation of cell differentiation
42598	74	10	0.00	vesicular fraction
6936	76	10	0.00	muscle contraction
3012	76	10	0.00	muscle system process
4857	98	10	0.02	enzyme inhibitor activity
1501	104	10	0.03	skeletal development
48878	105	10	0.04	chemical homeostasis
16477	109	10	0.04	cell migration
44449	41	9	0.00	contractile fiber part
43292	43	9	0.00	contractile fiber
16616	48	9	0.00	oxidoreductase activity, acting on the CH-OH group of donors, NAD or NADP as acceptor
48771	52	9	0.00	tissue remodeling
8015	66	9	0.01	blood circulation
3013	66	9	0.01	circulatory system process
8202	71	9	0.01	steroid metabolic process
16758	79	9	0.02	transferase activity, transferring hexosyl groups
40008	85	9	0.03	regulation of growth
42803	87	9	0.03	protein homodimerization activity
30016	40	8	0.00	myofibril
31214	47	8	0.00	biomineral formation
1503	47	8	0.00	ossification

46849	49	8	0.00	bone remodeling
45786	74	8	0.03	negative regulation of cell cycle
6631	83	8	0.05	fatty acid metabolic process
30017	38	7	0.00	sarcomere
8083	47	7	0.01	growth factor activity
19842	57	7	0.02	vitamin binding
42060	59	7	0.03	wound healing
16051	62	7	0.03	carbohydrate biosynthetic process
60047	22	6	0.00	heart contraction
3015	22	6	0.00	heart process
45596	30	6	0.00	negative regulation of cell differentiation
51270	37	6	0.01	regulation of cell motility
9725	41	6	0.02	response to hormone stimulus
44420	42	6	0.02	extracellular matrix part
14706	44	6	0.02	striated muscle development
7596	46	6	0.03	blood coagulation
50817	46	6	0.03	coagulation
4866	48	6	0.03	endopeptidase inhibitor activity
7599	48	6	0.03	hemostasis
30414	49	6	0.03	protease inhibitor activity
8194	50	6	0.04	UDP-glycosyltransferase activity
20037	51	6	0.04	heme binding
46906	51	6	0.04	tetrapyrrole binding
4252	52	6	0.04	serine-type endopeptidase activity
55080	53	6	0.05	cation homeostasis
50878	55	6	0.05	regulation of body fluid levels
6937	18	5	0.00	regulation of muscle contraction
48839	19	5	0.00	inner ear development
48545	20	5	0.00	response to steroid hormone stimulus
43583	21	5	0.00	ear development
51287	22	5	0.00	NAD binding
21700	22	5	0.00	developmental maturation
8375	23	5	0.00	acetylglucosaminyltransferase activity
42692	23	5	0.00	muscle cell differentiation
7584	23	5	0.00	response to nutrient
30170	26	5	0.01	pyridoxal phosphate binding
7188	29	5	0.01	G-protein signaling, coupled to cAMP nucleotide second messenger
19933	30	5	0.02	cAMP-mediated signaling
31667	30	5	0.02	response to nutrient levels
7519	31	5	0.02	skeletal muscle development
30334	32	5	0.02	regulation of cell migration
4867	32	5	0.02	serine-type endopeptidase inhibitor activity
9991	33	5	0.02	response to extracellular stimulus

7187	34	5	0.03	G-protein signaling, coupled to cyclic nucleotide second messenger
19935	36	5	0.03	cyclic-nucleotide-mediated signaling
42445	36	5	0.03	hormone metabolic process
7423	40	5	0.05	sensory organ development
30005	41	5	0.05	cellular di-, tri-valent inorganic cation homeostasis
55066	41	5	0.05	di-, tri-valent inorganic cation homeostasis

Supplemental Table S6-5: Cluster 5 associated GO Categories. Statistically significant GO categories (p < 0.05 as determined by the GoMiner software).

GO ID	Total	Change	P-Value (Changed)	Term
3824	2092	35	0.04	catalytic activity
9058	597	16	0.00	biosynthetic process
44249	439	13	0.00	cellular biosynthetic process
5739	456	13	0.01	mitochondrion
6807	172	11	0.00	nitrogen compound metabolic process
9308	160	9	0.00	amine metabolic process
44429	292	9	0.01	mitochondrial part
31975	292	9	0.01	envelope
31967	292	9	0.01	organelle envelope
3723	334	9	0.03	RNA binding
6519	136	8	0.00	amino acid and derivative metabolic process
6082	238	8	0.01	organic acid metabolic process
9059	317	8	0.05	macromolecule biosynthetic process
6520	110	7	0.00	amino acid metabolic process
31966	197	7	0.01	mitochondrial membrane
5740	205	7	0.02	mitochondrial envelope
6412	211	7	0.02	translation
9605	230	7	0.03	response to external stimulus
19752	236	7	0.03	carboxylic acid metabolic process
5743	156	6	0.02	mitochondrial inner membrane
19866	169	6	0.02	organelle inner membrane
51254	115	5	0.02	positive regulation of RNA metabolic process
45893	115	5	0.02	positive regulation of transcription, DNA-dependent
45941	140	5	0.04	positive regulation of transcription
45935	143	5	0.04	positive regulation of nucleobase, nucleoside, nucleotide and nucleic acid metabolic process
9611	158	5	0.05	response to wounding

Supplemental Table S6-6: Cluster 6 associated GO Categories. Statistically significant GO categories ($p < 0.05$ as determined by the GoMiner software).

GO ID	Total	Change	P-Value (Changed)	Term
5575	5217	104	0.05	cellular_component
5737	2739	62	0.03	cytoplasm
16020	2018	54	0.00	membrane
19538	1421	38	0.01	protein metabolic process
44260	1363	34	0.04	cellular macromolecule metabolic process
44267	1321	33	0.05	cellular protein metabolic process
16021	1285	32	0.05	integral to membrane
50896	771	30	0.00	response to stimulus
16787	842	30	0.00	hydrolase activity
51234	1051	27	0.05	establishment of localization
5886	869	24	0.03	plasma membrane
2376	223	19	0.00	immune system process
6955	146	18	0.00	immune response
60089	556	18	0.02	molecular transducer activity
4871	556	18	0.02	signal transducer activity
8233	215	16	0.00	peptidase activity
5576	477	16	0.02	extracellular region
6508	279	15	0.00	proteolysis
4175	147	13	0.00	endopeptidase activity
45184	347	13	0.01	establishment of protein localization
8104	366	13	0.02	protein localization
65008	383	13	0.03	regulation of biological quality
33036	387	13	0.03	macromolecule localization
30234	309	12	0.01	enzyme regulator activity
15031	333	12	0.02	protein transport
9605	230	11	0.00	response to external stimulus
6915	336	11	0.05	apoptosis
12501	338	11	0.05	programmed cell death
9611	158	10	0.00	response to wounding
31410	195	10	0.00	cytoplasmic vesicle
31982	201	10	0.00	vesicle
5829	281	10	0.04	cytosol
6629	287	10	0.04	lipid metabolic process
4888	220	9	0.02	transmembrane receptor activity
42221	213	8	0.05	response to chemical stimulus
6952	139	7	0.02	defense response
4252	52	6	0.00	serine-type endopeptidase activity
8236	58	6	0.00	serine-type peptidase activity
17171	59	6	0.00	serine hydrolase activity
1775	68	6	0.00	cell activation
5768	84	6	0.01	endosome
4857	98	6	0.01	enzyme inhibitor activity
22804	136	6	0.04	active transmembrane transporter activity
19882	10	5	0.00	antigen processing and presentation
2449	33	5	0.00	lymphocyte mediated immunity
2460	35	5	0.00	adaptive immune response based on somatic recombination of immune receptors built from immunoglobulin superfamily domains
2250	35	5	0.00	adaptive immune response
2443	37	5	0.00	leukocyte mediated immunity
44440	41	5	0.00	endosomal part
10008	41	5	0.00	endosome membrane
2252	50	5	0.00	immune effector process
50878	55	5	0.00	regulation of body fluid levels

2682	65	5	0.01	regulation of immune system process
6873	76	5	0.01	cellular ion homeostasis
55082	77	5	0.02	cellular chemical homeostasis
50801	83	5	0.02	ion homeostasis
5764	86	5	0.02	lysosome
323	86	5	0.02	lytic vacuole
6954	96	5	0.04	inflammatory response
5773	100	5	0.04	vacuole
48878	105	5	0.05	chemical homeostasis
