# Supplementary material for: Studies on Xenopus laevis intestine reveal biological pathways underlying vertebrate gut adaptation from embryo to adult
Source: Genome Biol. 2010 May 19;11(5):R55. doi: 10.1186/gb-2010-11-5-r55 (PMC2898076; doi:10.1186/gb-2010-11-5-r55)
Supplement: Additional file 2 — Figure S1. A PDF file containing a supplementary figure. [file gb-2010-11-5-r55-S2.PDF]

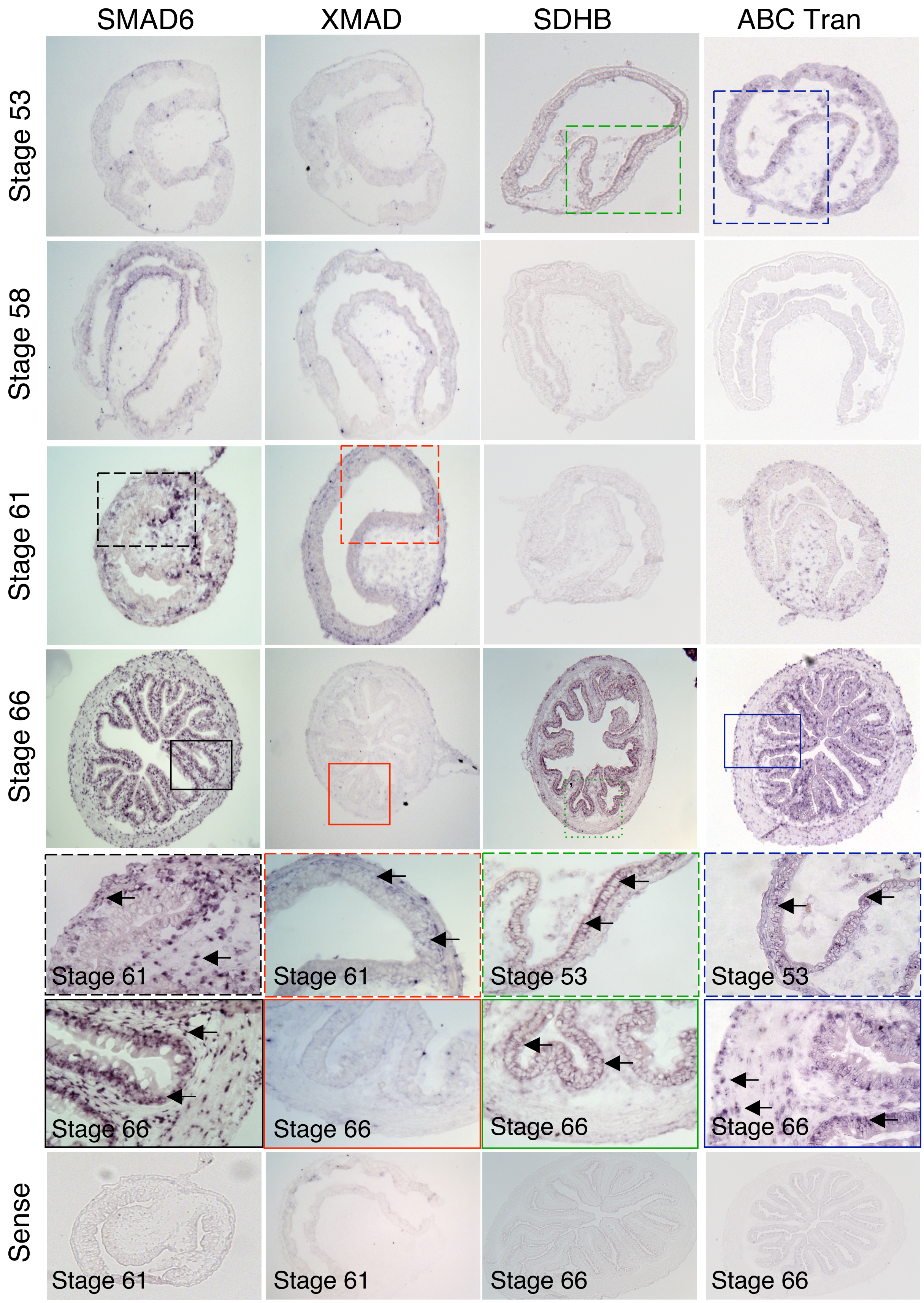


**Supplemental Fig 1**

- **Supplemental Fig 1S.** The change in mRNA expression of genes from cluster 1 (*SMAD6* and *XMAD*) and cluster 2 (*SDHB* and *ABC Tran)* in the intestine of during metamorphosis. The expression of *SMAD6 and XMAD* peaks at metamorphosis (stage 61), while the expression of *SDHB* and *ABC Tran* is high at stage 53 and 66, with little or no expression at stage 61. At metamorphic climax, *SMAD6* is expressed in the connective tissue and muscle, while *XMAD* is expressed exclusively in the connective tissue. *SDHB* is exclusively expressed in the epithelium, where its expression is high in the larval epithelium at stage 53. No expression is detected at stage 58 or 61, while the expression of SDHB returns in the adult epithelium at stage 66. *ABC Tran* is expressed in all tissue types at the beginning and end of metamorphosis. The bottom panel for each gene represents the negative control with a sense probe.
